# Identification of NpdA as the protein forming the surface layer in *Paracidovorax citrulli* and evidence of its occurrence as a surface layer protein in diverse genera of the Betaproteobacteria and Gammaproteobacteria

**DOI:** 10.1099/acmi.0.000685.v3

**Published:** 2023-12-11

**Authors:** Shabda Gajbhiye, Erin D. Gonzales, Daniel B. Toso, Natalie A. Kirk, William J. Hickey

**Affiliations:** ^1^​ Department of Bacteriology, University of Wisconsin, Madison, Wisconsin, USA; ^2^​ Department of Soil Science, University of Wisconsin, Madison, Wisconsin, USA; ^†^​ Present address: California Institute for Quantitative Biosciences, University of California, Berkeley, California, USA; ^‡^​ Present address: Department of Art and Art History, University of Utah, Salt Lake City, Utah, USA

**Keywords:** bacterial fruit blotch, biofilm, cryo-electron microscopy, nanopod, surface layer, surface layer protein

## Abstract

The phytopathogen *Paracidovorax citrulli* possesses an ortholog of a newly identified surface layer protein (SLP) termed NpdA but has not been reported to produce a surface layer (S-layer). This study had two objectives. First, to determine if *P. citrulli* formed an NpdA-based S-layer and, if so, assess the effects of S-layer formation on virulence, production of nanostructures termed nanopods, and other phenotypes. Second, to establish the distribution of *npdA* orthologs throughout the Pseudomonadota and examine selected candidate cultures for physical evidence of S-layer formation. Formation of an NpdA-based S-layer by *P. citrulli* AAC00-1 was confirmed by gene deletion mutagenesis (Δ*npdA*), proteomics, and cryo-electron microscopy. There were no significant differences between the wild-type and mutant in virulence assays with detached watermelon fruit. Nanopods contiguous with S-layers of multiple biofilm cells were visualized by transmission electron microscopy. Orthologs of *npdA* were identified in 62 Betaproteobacteria species and 49 Gammaproteobacteria species. In phylogenetic analyses, NpdA orthologs largely segregated into distinct groups. Cryo-electron microscopy imaging revealed an NpdA-like S-layer in all but one of the 16 additional cultures examined. We conclude that NpdA represents a new family of SLP, forming an S-layer in *P. citrulli* and other Pseudomonadota. While the S-layer did not contribute to virulence in watermelon fruit, a potential role of the *P. citrulli* S-layer in another dimension of pathogenesis cannot be ruled out. Lastly, formation of cell-bridging nanopods in biofilms is a new property of S-layers; it remains to be determined if nanopods can mediate intercellular movement of materials.

## Graphical abstract

Stylized cryo-electron image of two bacterial cells and an extracellular vesicle highlighting the presence of a surface layer (gold-colored structure).
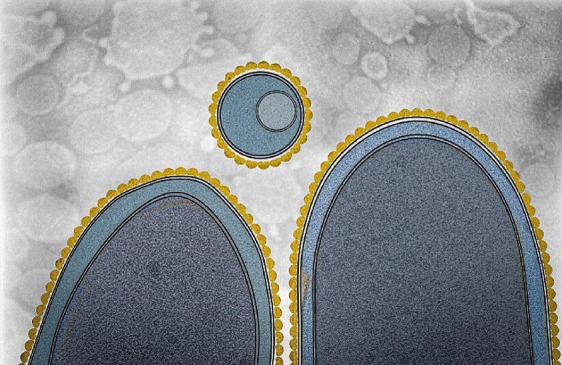

## Data summary

All supporting data and protocols have been provided within the article or through supplementary data files.

## Introduction

Surface layers (S-layers) are paracrystalline arrays, that are typically composed of a single surface layer protein (SLP) and cover the outer surfaces of prokaryotic cells. Hundreds of different archaeal and bacterial taxa are known to produce S-layers [1]. While the phylogenetic distribution of S-layers is relatively broad, the number of documented occurrences is small in comparison to the entire prokaryotic world consisting of tens of thousands of described species. A comprehensive search of genomic databases for SLP distribution (and potential S-layer formation) is constrained by the high sequence diversity of SLP [2] that can extend to species of the same genus [3]. Thus, empirical investigation is required to discover and validate new SLP.

The biological roles of S-layers have been explored in detail for only a small subset of S-layer producers [4, 5] and two general types of functions have emerged. Most often, the S-layer serves as an external coat, which can serve a variety of functions such as modulating translocation of materials into the cells (phage, *Bdellovibrio *, lytic enzymes, minerals), providing a scaffold for exoproteins or contributing to cell structure/stabilization [5]. Another general function is to mediate interactions with heterotypic biological surfaces (i.e. eukaryotic cells and tissues) that can contribute to pathogenesis or probiosis [5].

However, there is a third, largely overlooked S-layer function/property, which is mediating homotypic interactions with other S-layers comprised of the same SLP [6, 7]. For example, *in vitro* studies done with SLP extracted from *Deinococcus radiodurans* and *Sporosarcina ureae* demonstrated that S-layers of each of these organisms interacted specifically and resulted in formation of continuous pores through the outer surfaces [6]. These SLP junctions were termed ‘connexons’ after those known to connect eukaryotic cells, and were hypothesized to enable intercellular exchange of materials [6]. Connexons could potentially serve as a type of kin selection [8] and if so, the high sequence divergence of SLP could in part reflect a mechanism to define and tune the specificity of S-layer interactions [9].

Recently, NpdA was identified as the SLP creating an S-layer in *Delftia acidovorans* Cs1-4 [10]. This S-layer was essential for the formation of nanopods, which were S-layer protrusions containing outer membrane vesicles (OMV) [10, 11]. Imaging of *D. acidovorans* Cs1-4 biofilm by three-dimensional scanning electron microscopy (3D SEM) of thick (1250–1500 nm) sections revealed native structure of intact nanopods as cell-attached, polymorphic structures [12]. The 3-D SEM data established that nanopods were physically contiguous with S-layers of neighboring cells, and revealed openings into cells at the nanopod junctions. These characteristics of nanopods were consistent with the physical properties described for connexons [6]. It remains undetermined, however, whether or not nanopods mediate intercellular transfer of materials as hypothesized for connexons [6].

Shetty and coworkers identified several additional bacteria as potential producers of an NpdA-based S-layer; these candidate organisms were identified via possession of a genomic locus predicted to encode an NpdA ortholog and by visualization of detached, nanopod-like fragments in culture fluids [10]. One of these cultures was the phytopathogen *Paracidovorax citrulli*, previously classified as *Acidovorax citrulli* [13], the causal agent of bacterial fruit blotch disease of cucurbits [13–15] for which S-layer production had not been described. Since S-layers contribute to the virulence of some animal pathogens, establishing the existence of an S-layer and the identity of the constituent SLP is an important knowledge gap to fill for *P. citrulli*.

The goals of this study were two-fold. First, to determine if *P. citrulli* forms an NpdA-based S-layer and, if so, provide an initial characterization of the structure in terms of physicochemical impacts, a potential role in virulence, and nanopod production in biofilms. Second, to assess the broader distribution of *npdA* orthologs via bioinformatics and utilize that information to select candidates for physical examination of S-layer formation.

## Methods

### Cultures

Cultures were obtained from the Leibniz Institute DSMZ (DSMZ; Braunschawg, Germany), the American Type Culture Collection (ATCC; Manassas, VA) or the laboratories of individual investigators (Table 1). All Betaproteobacteria cultures were grown in Nutrient Broth (NB; BD-Difco; Vernon Hills, IL), except *Paracidovorax valerianellae* which was grown in Trypto-Casein-Soy medium (BD-Difco) and *Verminephrobacter eiseniae *, for which *Acidovorax* complex medium was used [16]. Cultures of Gammaproteobacteria were grown in either Marine Broth 2216 (BD-Difco, *Halia salexigens*), R2A medium (BD-Difco, *Rudaea cellulosilytica *) or *Congregibacter* medium (DSMZ medium 1115).

**Table 1.** Cultures examined in the S-layer studies

Genus and species	Strain	Source
----------------------------------- Betaproteobacteria -------------------------------		
* Acidovorax delafieldii *	2AN	Eric Roden
* Acidovorax radicis *	DSM-23535	DSMZ
*Paenacidovorax caeni*	DSM-19327	DSMZ
*Paracidovorax anthurii*	DSM-16745	DSMZ
*Paracidovorax avenae*	ATCC 19860	ATCC
*Paracidovorax cattleyae*	DSM-17101	DSMZ
*Paracidovorax citrulli*	AAC00-1	Ronald Walcott
*Paracidovorax konjaci*	DSM-7481	DSMZ
*Paracidovorax oryzae*	ATCC 19882	ATCC
*Paracidovorax valerianellae*	DSM-16619	DSMZ
*Delftia acidiovorans*	ATCC 15688	ATCC
* Ottowia thiooxydans *	DSM-14619	DSMZ
* Simplicispira psychrophila *	DSM-11588	DSMZ
* Verminephrobacter eiseniae *	EF01-2	David Stahl
----------------------------------- Gammaproteobacteria -------------------------------		
* Congregibacter litoralis *	KT71 (DSM-17192)	DSMZ
* Haliea salexigens *	DSM-19537	DSMZ
* Rudaea cellulosilytica *	DSM-22992	DSMZ

### Protein analyses by SDS-PAGE

Cultures of *P. citrulli* were grown in 25 ml of NB until late log phase. For whole cell protein profiles, a culture sample (100 µl) was washed twice with sterile phosphate buffer (10 mM, pH 7.5), and the final cell pellet mixed with100 µl Laemmli sample buffer (Bio-Rad Laboratories; Hercules, CA). Cell lysis was achieved by heating (5 min, 100°C) the samples followed by centrifugation (10 min, 6 000×*g*) to remove particulates. Supernatant (20 µl) from the extracts was placed in wells of a of 4–20 % Triton Min-Protean TGX PreCast gel (Bio-Rad). The ProPrecision Plus Protein Dual Colour Standard (Bio-Rad) was included in the gels for reference. Electrophoresis was done in Tris/Glycine/SDS buffer (Bio-Rad) at 100 V for 1.5 h. The gel was then washed for 1 h in 50 ml distilled deionized (dd) H_2_O with several changes, followed by *ca*. 30 min staining with Bio-Safe Coomassie stain (Bio-Rad) and then destaining 12 h in dd H_2_O.

For SLP extraction and analysis, cells were harvested from 25 ml of NB by centrifugation (20 min, 6 000×*g*, 4°C). Cell pellets were then re-suspended in 3 ml 5M LiCl [17] and the cell suspensions mixed (50 r.p.m.) on a Solaris orbital shaker (Thermo Fisher). Particulate material was then removed by centrifugation (60 min, 6 000×*g*, 4°C), and the extracts were loaded into a Slide-A-Lyzer (2 kDa MWCO, Thermo Fisher) cassette and dialysed against sterile dd H_2_O overnight. The extracts were freeze-dried, and then reconstituted in 100 µl of sterile dd H_2_O. Samples were mixed with 2X Laemmlis ample buffer (Bio-Rad) and then loaded on a 12 % Triton Min-Protean TGX Precast gel (Bio-Rad). Electrophoresis was done at 100 V for 25 min, in 25 mM Tris, 192 mM glycine, 0.1 % SDS (pH 8.3). Gels were washed for 1 h in dd H_2_O and then stained and destained as described above.

### Protein mass spectrometry

Methods outlined by Shetty *et al*. [10] were followed for protein identification by mass spectrometry. Briefly, the putative NpdA band recovered in the SLP extract was excised from the SDS-PAGE gel, the gel slice chopped into small pieces, and the pieces transferred to a LoBind tube (Eppendorf; Enfield, CT) for further processing and Trypsin digestion. Tryptic digests were analyzed by LTQ Orbitrap XL (ThermoScientific; Waltham, MA). Mass spectral data was searched against the *P. citrulli* AAC00-1 genome by using Mascot (Matrix Science, London, UK; version Mascot) and peptide assignments made with Mascot were validated with Scaffold (Proteome Software Inc., Portland, OR).

### Cryo-electron microscopy (Cryo-EM)

Samples of late log-phase liquid cultures (Table 1) were placed on glow-discharged Quantifoil R2/2 300 Mesh grids and were vitrified by plunge freezing in liquid ethane by using a Mark IV Vitribot (FEI, Hillsboro, OR). Samples were imaged with a Tecnai G2 F30 300 kV scanning TEM (FEI) fitted with a K2 Summit 14.2 Megapixel direct detection camera (Gatan, Pleasenton, CA) and a Gatan K2 direct electron detector. Images were processed with digital micrograph software (Gatan) as well as SerialEM (Univ. Colorado, Boulder). The Cryo-EM images presented in this study are deposited in the EMBL-EBI Biostudies database (https://www.ebi.ac.uk/biostudies) under accession S-BSST1223.

### Mutagenesis

Molecular manipulations were designed to target the *P. citrulli* AAC00-1 locus AAVE_RS20655 (Fig. S1, available in the online version of this article) for deletion by allelic exchange with the Cre-*lox* system following methods described by Denef *et al*. [18]. All strains, plasmids and PCR primers used in mutagenesis are listed in Table S2. Genomic DNA was prepared from *P. citrulli* strain AAC00-1 by using a Genomic DNA Purification Kit (ThermoFisher; Waltham, MA). Regions flanking *npdA* were amplified by PCR (Table S2) from the genomic DNA template by using One *Taq* Master Mix (New England BioLabs; Ipswich, MA; RRID: SCR 013517). The upstream flanking region amplified was a 521 bp fragment (‘npdA Up’, Fig. S1) and the flanking downstream region was a 506 bp fragment (‘npdA Down’, Fig. S1). The PCR products were subcloned by blunt-end ligation into pCR-Blunt (ThermoFisher; RRID: Addgene_112638) and then transformed into *E. coli* One Shot (ThermoFisher) chemically competent cells according to the manufactuer’s protcols. Vectors containing the subcloned fragments were extracted from *E. coil* by using the Qiagen Plasmid Midi kit (Qiagen, Germantown, MD). The ‘npdA Up’ and ‘npdA Down’ fragments were released from the vectors by double digestion with the appropriate pair of restriction enzymes (Table S2). The enzyme digests were separated in a 1 % agarose gel (1X TAE); fragments of interest were excised in gel slices and purified by using a Qiagen gel purification kit (Qiagen). The digested fragments were then inserted sequentially into the desired multiple cloning site of pJK100 (RRID: Addgene73264) by using T4 DNA ligase (New England Biolabs). For each of these ligations, the vector was prepared by double digestion with the pair of restriction enzymes appropriate to generate annealing sites complementary to those of the inserted fragment. First, the ‘npdA Up’ fragment was inserted and the resulting construct transformed into chemically-competent *E. coli* WM3064. The construct was then extracted and purified by using the NucleoBond Xtra Midi kit (Macherey-Nagel; Allentown, PA) and the process repeated for the ‘npdA Down’ fragment resulting in the mutagenesis vector pEG100. Methods described by Denef *et al*. [18] were employed for conjugal transfer of pEG100 from *E. coli* WM3064 to *P. citrulli* AAC00-1 and transconjugants obtained by antibiotic selection. Occurrence of the desired double cross-over resulting in deletion of *npdA* and introduction of *kan* confirmed by PCR. In this PCR assay, the primers (Table S2) kanR/npdA External F1and the kanF/npdA External R1 gave the expected products of *ca*. 1530 bp and 1610 bp, respectively, from the intermediates. Subsequently, pCM157 (RRID: Addgene45863) was transferred from *E. coli* WM3064 to *P. citrulli* AAC00-1 by conjugation. The recombinase (Cre) encoded by pCM157 catalyses the excision of the region between the *lox* sites, which in this case included the kanamycin marker and *npd*A. The Cre expression vector was cured by passage of the Δ*npdA* mutant through LB medium lacking antibiotic selection (Fig. S1).

### Batch culture growth measures

Aliquots (1 ml) of overnight (16 h) NB-grown cultures of *P. citrulli* AAC00-1 WT and Δ*npdA* mutant were spun down (6 000×*g *for 10 min), washed twice with 1 ml sterile NB, and then resuspended in sterile NB to OD590=0.25 (*ca*. 10^7^ c.f.u. ml^−1^) as measured by a BioTek Synergy 2, Multi-detection Microplate Reader (Agilent; Santa Clara, CA). Triplicate cultures (50 ml NB) of *P. citrulli* AAC00-1 WT and Δ*npdA* mutant were inoculated with 100 µl of the diluted cell suspension. The cultures were then incubated with mixing (180 r.p.m.) on an orbital shaker at 28°C. For growth measurement, an aliquot (100 µl) was taken from each replicate were measured for OD590 in the microplate reader. Cultures were sampled at 2 h intervals for the first 12 h, and then at 4 h intervals until the experiment was terminated after 24 h of incubation.

### Biofilm formation assay

Cells were harvested from cultures (20 ml NB) of *P. citrulli* AAC00-1 (WT and Δ*npdA*) by centrifugation at 6 000×*g *for 10 min. The cell pellets were then resuspended in sterile dd H_2_O to an OD590 of 1.0 as measured with the microplate reader. Cell suspensions (200 µl) were aliquoted in triplicate into wells of Primaria Microtest Microplates (96-well with mixed charge coating, Corning Inc; Corning, NY). The plate was incubated statically at 28°C. After 48 h, cell suspensions were removed by pipette, and the wells washed three times with 200 µl of dd H_2_O. The biofilms adhering to the wells were stained with 1 % (w/v) crystal violet (Sigma Aldrich, St. Louis, MO) for 45 min and then washed three times with sterile dd H_2_O. Crystal violet retained by the adherent cells was extracted by the addition of 3 ml 95 % ethanol. Triplicate non-inoculated wells were used as negative controls. After 2 h incubation, the ethanol solution was removed, and the absorbance (A590) of the extracted crystal violet was measured by the microplate reader. Readings were corrected by subtraction of the background levels of crystal violet retained in non-inoculated wells. The data was analyzed by a paired sample two-tailed T-test using the Graphpad (Graphpad Software; Boston, MA; RRID: SCR_000306) online T-test calculator (https://www.graphpad.com/quickcalcs/ttest1).

### Transmission electron microscopy (TEM) of *P. citrulli* AAC00-1 culture fluids and biofilms

Culture fluid samples were negatively stained by mixing with a solution of 2.0 % phosphotungstic acid and then imaged directly. The biofilm experiment followed a design described by Shetty *et al*. [10]. Glass coverslips (No. 1, 13 mm × 13 mm, CIDA; Overland Park, KS) were prepared by immersion in 30 % (v/v) HNO_3_, rinsing with sterile dd H_2_O and then sterilized by autoclaving (1 h, 121°C). A 20 ml aliquot of late-log phase culture of *P. citrulli* AAC00-1 WT grown on NB was transferred to a Petri dish, and sterile coverslips floated statically on the liquid surface for 18 h. The coverslips were then transferred to Petri dishes containing fresh NB and incubated statically in the dark at 28°C for 3 d. Biofilms were fixed by submerging coverslips in 2.5 % glutaraldehyde, then post-fixed in 0.1 % OsO_4_ followed by passage through an alcohol dehydration series terminating in 100% anhydrous ethanol (Electron Microscopy Science; Hatfield, PA). Molten, water-soluble Durcupan (Electron Microscopy Science) was placed over the biofilms, and following polymerization the resin block was removed with the adherent biofilm. Resin blocks were thin-sectioned (100 nm), and then stained by the two-step method, with uranyl acetate followed by lead citrate [19]. Sample imaging was done with a Philips CM120 scanning transmission electron microscope. Measurements of objects in TEM micrographs were done with Fiji (v. 2.14.0/1.54f; RRID: SCR_002285) software [20]. The TEM images presented in this study are deposited in the EMBL-EBI Biostudies database (https://www.ebi.ac.uk/biostudies) under accession S-BSST1223.

### Virulence assay with detached watermelon fruit

Cells of *P. citrulli* AAC00-1 (WT and Δ*npdA* mutant) were harvested from NB cultures by centrifugation at 6 000×*g* for 10 min, and the cell pellets resuspended in sterile dd H_2_O to OD590=0.25. The inoculant was then used in tests assaying virulence via differences in the size of external water-soaked lesions and internal tissue damage as indicated by decomposition and discoloration of interior rind tissues. For the latter test, three watermelon fruit (*Citrullus lanatus* cv. Melo-Glow) replicates were marked with three points one inch apart. The fruits were surface-sterilized with 70 % ethanol and each marked point was injected with 100 µl of inoculant or sterile dd H_2_O by using a sterile 1 ml tuberculin syringe and a sterile 18-gauge needle. The melons were then incubated at 28°C for 10 d. Disease was assessed by inspection of inoculation sites for surface blotch as well as for decomposition and discoloration of interior melon rind tissues. For examination of rind tissue, melons were cut along the three inoculation points perpendicular to the long axis of the fruit with a watermelon knife and the appearance of the melon cross sections was documented with a digital camera. The areas of discoloration were quantified by using Fiji software and the data was analyzed by a paired sample two-tailed T-test using the Graphpad online T-test calculator. For tests examining water-soaked lesions, two parallel rows of injection points were marked, each row containing five points, one inch apart. Melons were surface sterilized as above. For inoculation, a sterile graduated pipet tip was inserted to the 10 µl mark, creating an opening of *ca*. 1 mm diam. A 50 µl aliquot of inoculant was then added. The melons were then incubated at 28°C and after 10 d the surfaces were documented with a digital camera. The diameter of water-soaked lesions was measured at the widest point with Fiji software, and the data analyzed by a paired sample two-tailed T-test using the Graphpad Software online T-test calculator.

### Bioinformatics

The NpdA ortholog in *P. citrulli* AAC00-1 was retrieved under accession WP_011797163. Signal peptide prediction was done with SignalP 6 [21]. Orthologs of the *P. citrulli* AAC00-1 in other Pseudomonadota were detected by using the NCBI Blast-P tool with the query WP_011797163 (Supplementary data file S1). Phylogenetic analysis was done with mega 11 [22]. Evolutionary history was inferred by using the Maximum Likelihood method and JTT matrix-based model [23]. A bootstrap consensus tree was inferred from 500 replicates and the tree file exported to FigTree v. 1.4.4 [24] for illustration. Genomic neighborhoods of *npdA* orthologs were retrieved from the Integrated Microbial Genomes website [25].

## Results

### Protein analyses

The protein band recovered in the SLP extract (Fig. S2A) yielded a significant hit (sequence coverage=38 %, score=527, Expect=6.9e^−050^) to the predicted polypeptide of AAVE_RS20655 (Fig. S2B). The protein was annotated in Genbank as a ‘hypothetical protein’. The predicted polypeptide contains a 25 amino acid signal peptide diagnostic of a protein exported via a Type II Secretion System (T2SS) but otherwise lacks conserved domains (Fig. S2B). The expected molecular weight of the mature protein following signal peptide cleavage is 52 394 Da. In contrast, migration of the NpdA polypeptide in the SDS-PAGE gel at a position above the 150 kDa marker indicated the formation of an oligomer that was not disrupted by chaotropic salts such as 5 M LiCl that was used as the extractant or by denaturing agents used in SDS-PAGE.

### Confirmation of Δ*npdA* mutant

PCR amplification with primers external to *npdA* yielded the expected product of *ca*. 3.1 kb from the wild-type, which represented the 1.2 kb *npdA* gene and the two adjoining upstream or downstream regions (Fig. S3A, B). The expected PCR product was also amplified with the external primers from the mutant (Fig. 2b). At *ca*. 1.6 kb, the observed fragment was in accordance with the genome data as the flanking regions up- and down-stream distance minus *npdA*. Targeting of a region internal to *npdA* yielded the expected product of *ca*. 600 bp from the WT (Fig. S3B). In contrast, no PCR product was observed for the Δ*npdA* mutant (Fig. S2B). Whole cell protein analysis confirmed that the Δ*npdA* mutant lacked the band identified as NpdA (Fig. S3C).

### Cryo-EM of *P. citrulli* AAC00-1 WT and Δ*npdA* mutant

**Fig. 1.**
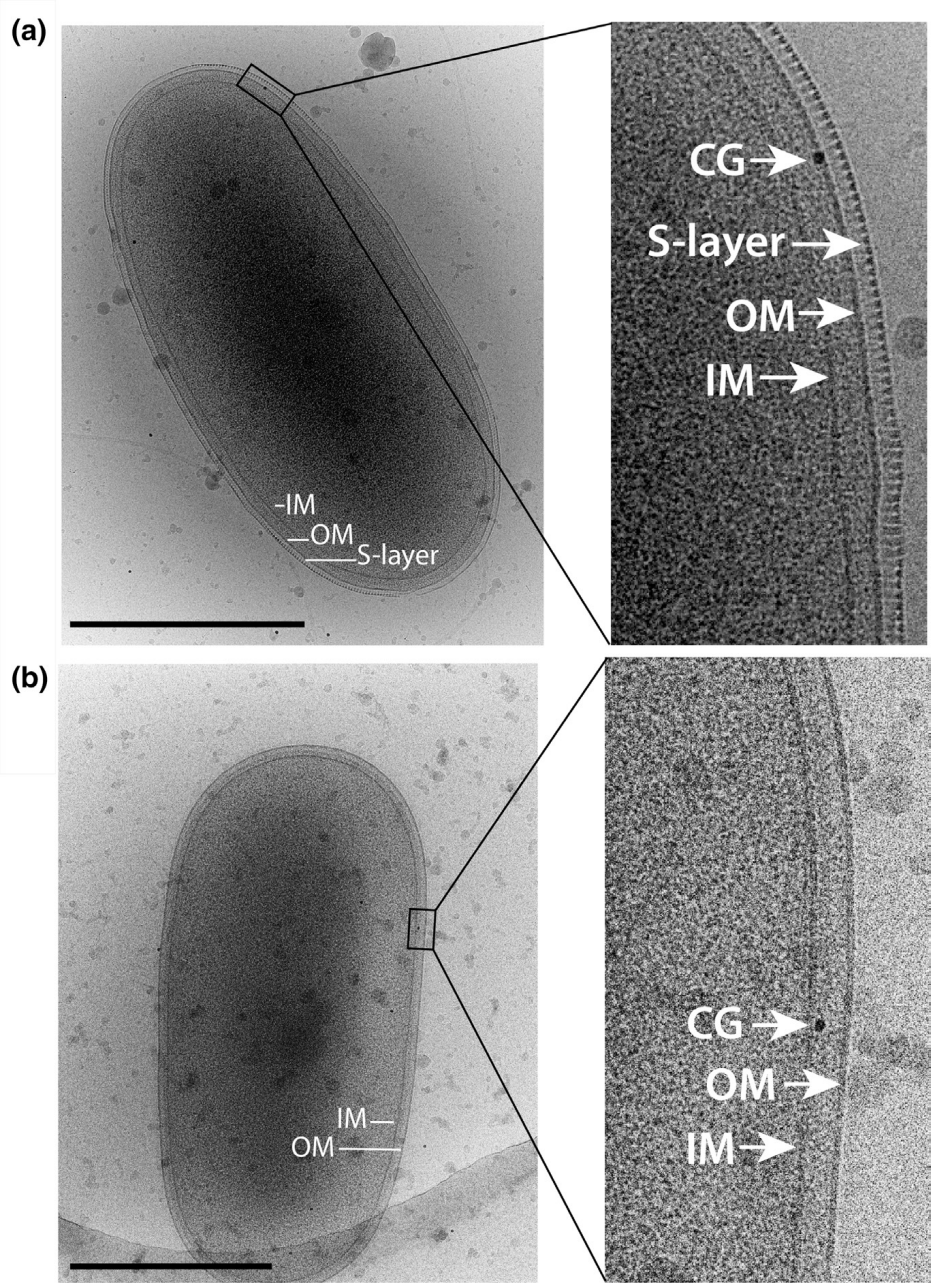
Cryo-electron microscopy imaging of *P. citrulli* AAC00-1 cells. Panel (a): wild-type, wide view of whole cell (left), and a detailed view of the cell membrane (right). Structures annotated are outer membrane (OM), inner membrane (IM) and surface layer (S-layer). A particle of 10 nm diam colloidal gold (CG) is also visible. Panel (b). Δ*npdA* mutant, wide view of whole cell (left), and a detailed view of the cell membrane (right) with a clear IM and OM but no S-layer. In both panels, the bar at lower left indicates 500 nm.

Imaging of cells by cryo-EM clearly revealed intact envelopes in their native state. In the WT cells, structures clearly discernible were the inner- and outer-membranes as well as an S-layer (Fig. 1a). The S-layer appeared as a bead-like structure on top of the outer-membrane (Fig. 1b). In contrast, cryo-EM imaging of the Δ*npdA* mutant showed intact inner- and outer-membranes but the bead-like structure was absent. While the Δ*npdA* mutant lacked the S-layer there was no other obvious difference between the WT and Δ*npdA* mutant in the cell envelope structure or integrity.

### Phenotypic characterization of *P. citrulli* AAC00-1 Δ*npdA* mutant vs. WT

**Fig. 2.**
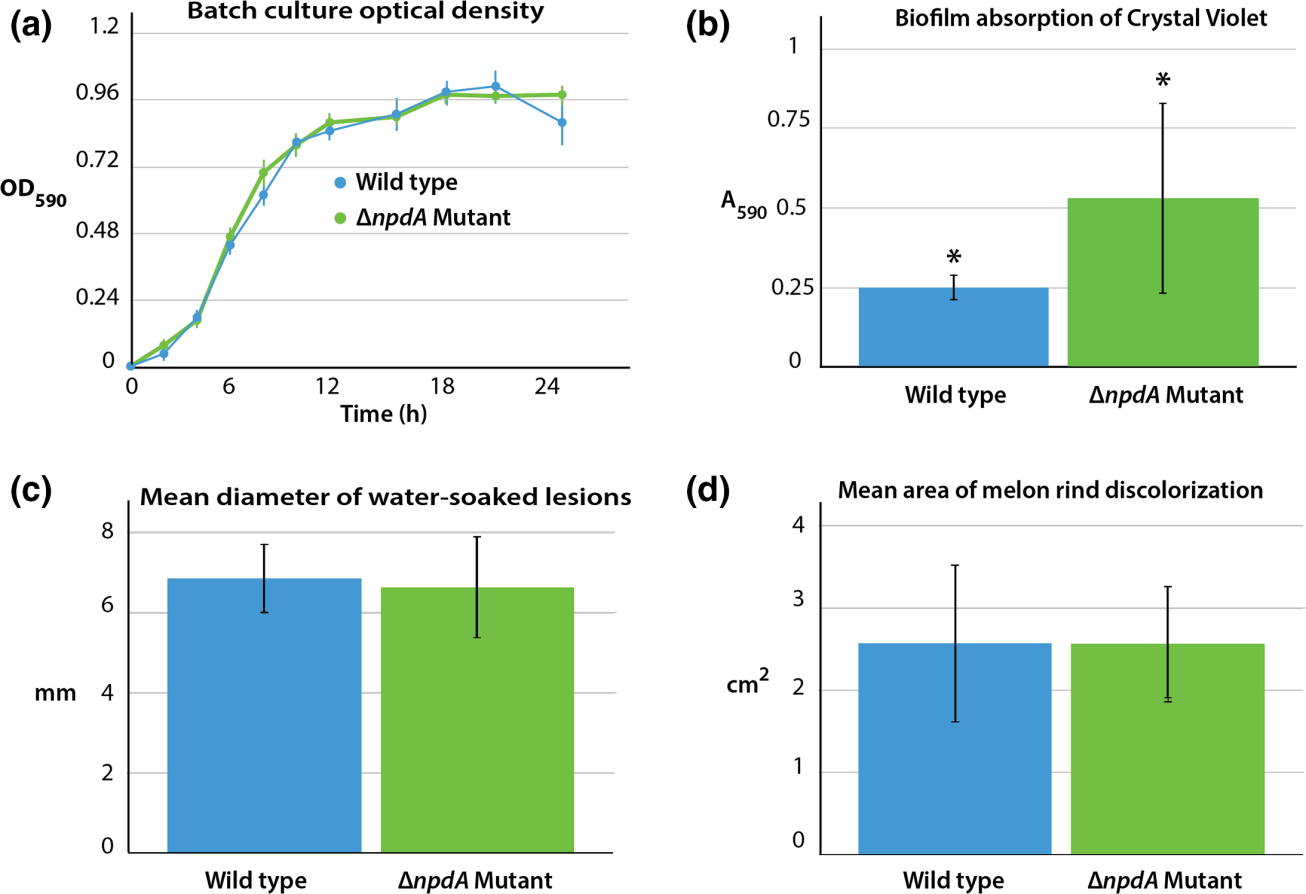
Phenotypic characterization of *P. citrulli* AAC00-1 wild-type vs. Δ*npdA* mutant. Panel (a): Growth curves in batch culture. Panel (b): Biofilm formation, *Indicates a significant difference between samples (*P*=0.0152). Panel (c): Water-soaked lesion formation on watermelon fruit. Panel (d): Discoloration of watermelon fruit rind.

There was no significant difference between the WT and Δ*npdA* mutant in growth characteristics measured in batch cultures, either in the growth rate or final culture density (Fig. 2a). In contrast, in the biofilm formation assay, crystal violet absorption by the Δ*npdA* mutant was significantly greater (*P*=0.0152) than that of the WT (Fig. 2b). The virulence tests with detached watermelon fruits showed no significant differences between the WT and Δ*npdA* mutant. The mean diameter of water-soaked lesions on WT-inoculated melons was not significantly different (*P*=0.2162) than that on melons inoculated with the Δ*npdA* mutant (Fig. 2c). Likewise, the mean area of melon rind discolorization in fruits inoculated with the WT was not significantly different (*P*=0.9870) from that of melons inoculated with the Δ*npdA* mutant (Fig. 2d).

### Examination of *P. citrulli* AAC00-1 for nanopods

**Fig. 3.**
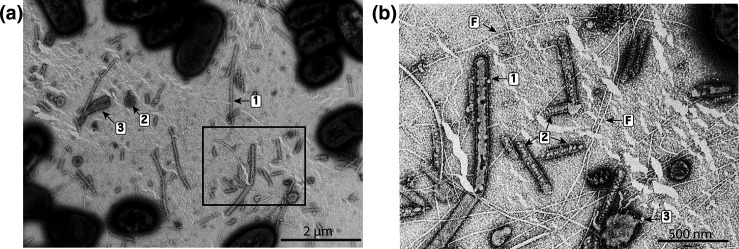
Culture fluids of *P. citrulli* AAC00-1 imaged by negative staining and TEM. Panel (a): Wide view showing cells and nanopod fragments of varying morphologies indicated by: 1) linear, 2) circular, 3) block-like. Panel (b). Detailed view of the area indicated by the rectangle in Panel (a) showing varying fragment morphologies and internal vesicle structure. Numbers indicate: 1) linear structure with a single, large vesicle, 2) linear structures with multiple, small vesicles, 3) circular structure containing a single, large vesicle. Fragments of flagella are indicated in Panel (b) by ‘F’.

**Fig. 4.**
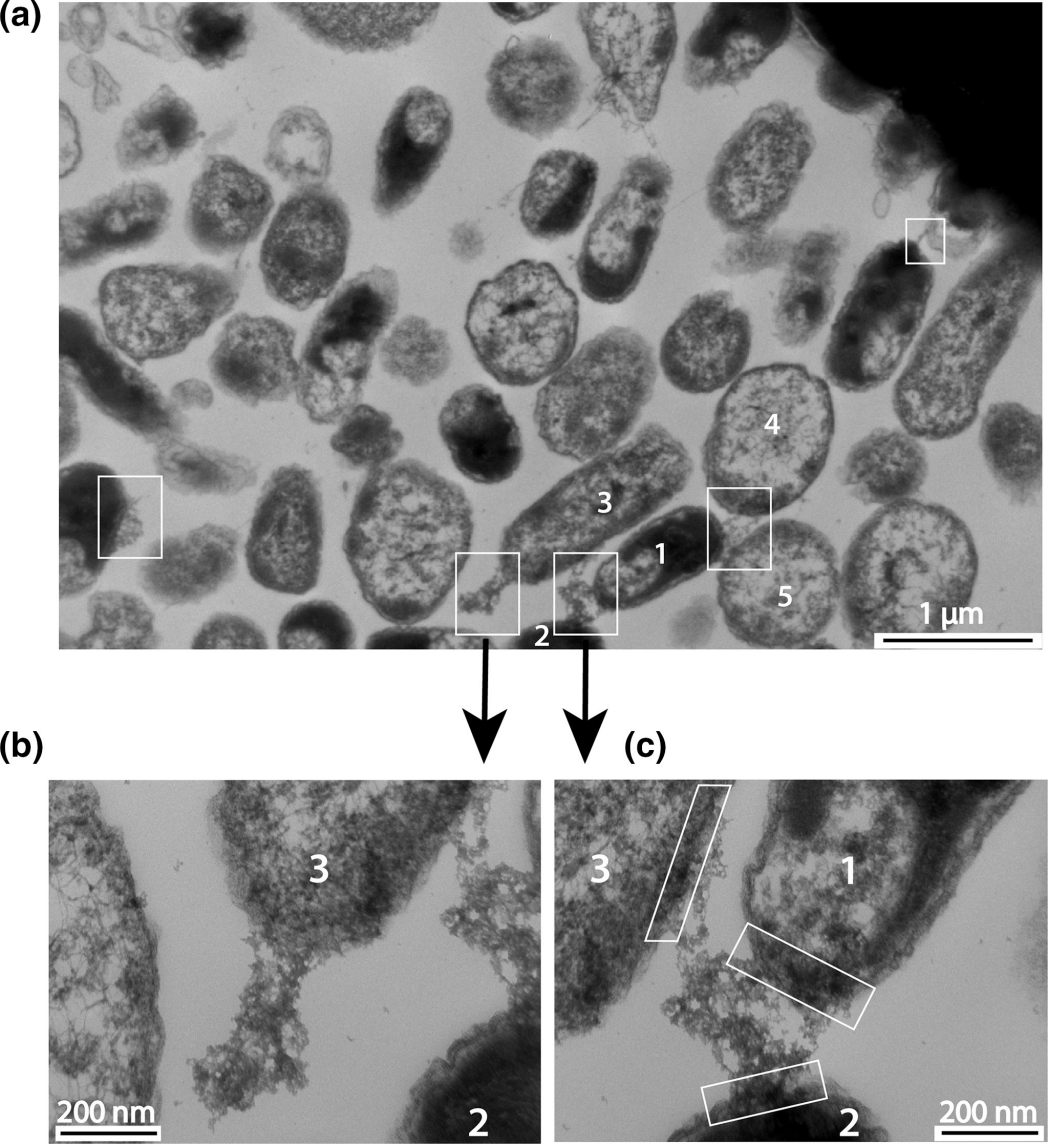
Biofilm of *P. citrulli* AAC00-1 WT imaged by TEM. Panel (a): Wide view of biofilm growth with examples of nanopods indicated by the white boxes. The numbering indicates an apparent network of cells joined via nanopods: Cell #1 is joined by a nanopod (lower left) to cells #2 and #3 and by another nanopod (upper right) to cells #4 and #5. Panels (b) and (c): Detailed views of nanopods and cells numbered in Panel (a). In Panel (c), the junctions of a single nanopod with three cells are indicated by the white boxes.

**Fig. 5.**
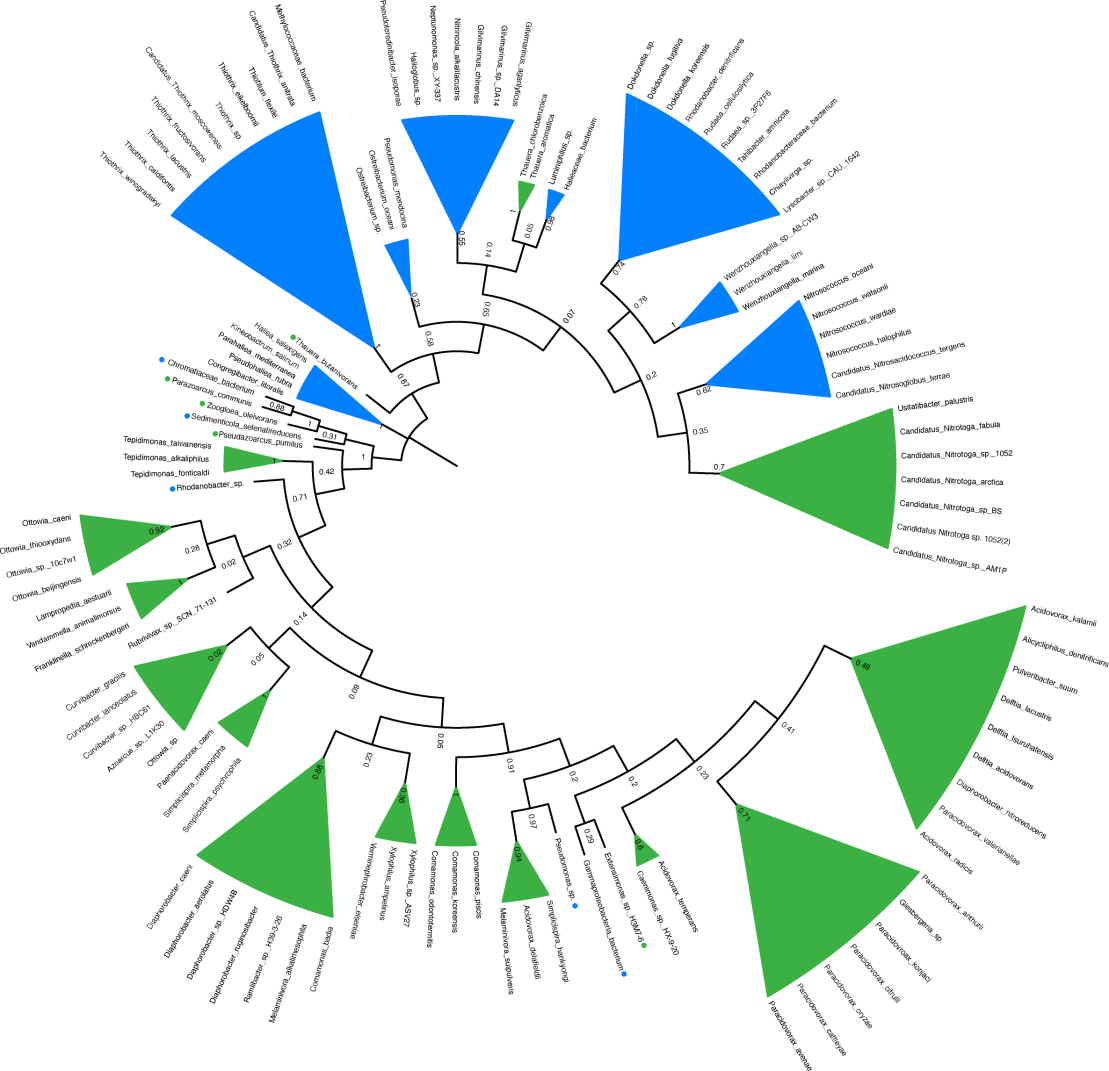
Maximum likelihood tree of NpdA orthologs in Betaproteobacteria (green leaves) and Gammaproteobacteria (blue leaves). Instances where a gammaproteobacterium occurs within a Betaproteobacteria leaf are indicated by a blue circle next to the organism's name. For leaves consisting of a single organism, a green- or blue-colored circle indicates its phylogenetic identity. Bootstrap values are given at the nodes. Sequences used to construct the tree are provided in Supplementary Data File S1 which is available with the online version of this paper.

Wet mounts of WT culture fluids contained an abundance of nanopod fragments that displayed three morphologies: linear, block-like and circular (Fig. 3a). Linear fragments were the most common morphology and ranged in length from <100 nm to *ca*. 1900 nm (Fig. 3a). Nanopods fragments contained vesicles, which appeared either as individual oval structures *ca*. 50 nm diam. or elongated and extended the length of the nanopod >500 nm (Fig. 3b). In contrast, wet mounts of the Δ*npdA* mutant culture fluids lacked nanopods (Fig. S4A–C). These fluids contained only fragments of flagella as well as spherical and globular extracellular vesicles (Fig. S4B, C). In biofilms of WT cells, nanopods were visualized primarily as block-like surficial protrusions (Fig. 4a–c). Nanopods often spanned the distance between neighbouring cells and formed broad junctions (>160 nm width) contiguous with cell surfaces. (Fig. 5c). An individual nanopod could form associations with two or more cells. Similarly, an individual cell could be associated with multiple nanopods (Fig. 4C).

### Occurrence of NpdA orthologs in other *P. citrulli* strains and in other Pseudomonadota

All strains of *P. citrulli* with currently available genomes possessed an *npdA* ortholog (Table S1). Orthologs of *npdA* were also identified in a wide range of Betaproteobacteria and Gammaproteobacteria (Tables 2 and 3). In the Betaproteobacteria, *npdA* was present in three orders: Burkholderiales**,** Nitrosomonadales and Rhodocyclales and five families (Table 2). Orthologs occurred most frequently in the *Comamondacaea* family of the Burkholderiales and were particularly common in the genus *Delftia* and the former genus *Acidovorax,* which is now divided into three genera: *Acidovorax, Paenacidovorax* and *Paracidovorax* [13]. There was a total of 19 genera and 45 species in the *Comamondacaea* that possessed *npdA*. All of the Betaproteobacteria species that harbored *npdA* were inhabitants of terrestrial/aquatic environments and were aerobic heterotrophs. One notable exception was the *Nitrotoga *, a recently described chemolithotrophic nitrite-oxidizer [26].

**Table 2.** Betaproteobacteria possessing an *npdA* ortholog*

Order	Family	Genus	Species†
Burkholderiales	Comamonadacea	* Acidovorax *	***delafieldii***
			*kalami*
			***radicis***
			*temperans*
		*Paenacidovorax*	***caeni***
		*Paracidovorax*	***anthurii***
			***avenae***
			***cattleyae***
			***citrulli***
			***konjaci***
			***oryzae***
			***valerianellae***
		* Alicycliphilus *	*denitrificans*
		* Comamonas *	*koreensis*
			*badia*
			*odontotermitis*
			*piscis*
		* Curvibacter *	*gracilis*
			*lanceolatus*
			species
		* Giesbergeria *	species
		* Delftia *	***acidovorans***
			*lacustris*
			*tsuruhatensis*
		* Diaphorobacter *	*aerolatus*
			*caeni*
			*ruginosibacter*
			*nitroreducens*
			species
		* Caenimonas *	species
		* Extensimonas *	species
		* Franklinella *	*schreckenbergeri*
		* Lampropedia *	*aestuarii*
		* Melaminivora *	*alkalimesophila*
			*suipulveris*
		* Ottowia *	*beijingensis*
			*caeni*
			***thiooxydans***
Burkholderiales	Comamonadacea	* Ottowia *	species
		*Puliveribacter*	*suum*
		* Simplicispira *	*hankyongi*
			*metamorpha*
			***psychrophila***
		* Verminephrobacter *	***eiseniae***
		* Vandammella *	*animalimorus*
	*S*phaerotilaceae	* Rubrivivax *	species
Nitrosomonadales	Gallionellaceae	*Candidatus* Nitrotoga	*fabula*
			species
			*arctica*
	Usitatibacteraceae	* Usitatibacter *	*palustris*
Rhodocyclales	Zoogloeaceae	* Azoarcus *	species
		* Parazoarcus *	*communis*
		*Psuedoazoarcus*	*pumilus*
		* Thauera *	*aromatica*
			*chlorobenzoica*
			*butanivorans*
		* Zoogloea *	*oleivorans*
Burkholderiales genera incertae sedis		* Tepidimonas *	*taiwanensis*
			*alkaliphilus*
			*fonticaldi*
		* Xylophilus *	species
			*ampelinus*

*Boldface type indicates organisms with cultures examined by cryo-electron microscopy.

†Organisms currently lacking taxonomic assignment to a unique species are identified as ‘species’.

**Table 3.** Gammaroteobacteria possessing *npdA* ortholog*

Order	Family	Genus	Species†
Cardiobacteriales	Cardiobacteriacea	* Ostreibacterium *	*oceani*
		* Congregibacter *	***litoralis***
		* Gilvimarinus *	*agarilyticus*
			*chinensis*
			species
Cellvibrionales	Halieaceae	Halieaceae bacterium	
		* Haliea *	***salexigens***
			species
		* Halioglobus *	species
		* Kineobactrum *	*salinum*
		* Luminiphilus *	species
		* Parahaliea *	*mediterranea*
		* Pseudohaliea *	*rubra*
	Cellivibrionaceae	* Pseudoteredinibacter *	*isoporae*
Chromatiales	Chromatiacaea	* Nitrosococcus *	*halophilus*
			*oceani*
			*wardiae*
			*watsonii*
	Wenzhouxiangellaea	* Wenzhouxiangella *	*limi*
			*marina*
			species
		*Candidatus* Nitrosoglobus	terrae
		*Candidatus* Nitrosacidococcus	tergens
	Chromatiacaea unclassified	Chromatiaceae bacterium	
Methylococcoales	Methylococoales unclassified	Methylococcaceae bacterium	
Oceansopiralaes	Oceanspiralacea	* Nitrincola *	*alkalilacustris*
		* Neptunomonas *	species
Pseudomonadales	Pseudomonadaceae	* Pseudomonas *	*mendocina*
			species
Thiotrochales	Thiotrichaceae	* Thiothrix *	*caldifontis*
			*eikelboomii*
			*lacustris*
			*fructosivorans*
			*winogradskyi*
			species
		*Candidatus* Thiothrix	*moscowensis*
			*anitrata*
Thiotrochales	Thiofilaceae	* Thiofilum *	*flexile*
Xanthomondales	Rhodanobacteracea	* Chiayiivirga *	species
		* Rhodanobacter *	*denitrificans*
		* Rudaea *	***cellulosilytica***
			species
		* Tahibacter *	*amnicola*
		Rhodanobacteracea bacterium	
	Xanthomondacea	* Lysobacter *	species
		* Dokdonella *	*koreensis*
			*fugitiva*
			species
Gammaproteobacteria incertae sedis		* Sedimenticola *	*selenatireducens*

*Boldface type indicates organisms with cultures examined by cryo-electron microscopy.

†Organisms currently lacking taxonomic assignment to a unique species are identified as ‘species’.

The Gammaproteobacteria in which *npdA* was identified spanned nine orders, 11 families, 31 genera, and 45 species (Table 3). Species with *npdA* were most common in the genus *Thiothrix *. At the order level, the Halieaceae had the greatest taxonomic diversity with *npdA* identified in seven of its constituent families. While the majority of the Gammaproteobacteria taxa identified were terrestrial/aquatic, there was a significant representation of marine organisms (18 species). A distinctive feature of this species collection was the diversity of growth modalities they displayed. While aerobic heterotrophs were the majority, there were also a number of genera that were chemolithotrophs, such as ammonia-oxidizers (* Nitrosococcus *), sulphide-oxidizers (* Thiothrix *), and photolithotrophs (* Congregibacter *). There was also one example of an anaerobic selenate-respiring bacterium (* Sedimenticola selenatireducens *).

The NpdA orthologs from the Betaproteobacteria vs. Gammaproteobacteria generally segregated into two separate branches of a phylogenetic tree (Fig. 5). But, in each half of the tree, there were some branches that had at least one representative from the other subdivision. Notably, the NpdA orthologs from the entire Betaproteobacteria genus *Nitrotoga* was located in the Gammaproteobacteria branch. Orthologs from most species of a genus generally clustered together on a single branch. However, in most cases, these clusters were not homogenous and also included one or more other genera. There were also cases where one or more orthologs from a given genus did not cluster with the rest. For example, orthologs from all *Paracidovorax* species clustered on a single branch except for *P. valerianellae* which segregated from the rest.

### Genome neighborhood of *npdA*

The genomic neighborhood of *npdA* includes an upstream cluster of 12 *gsp* (General Secretion Protein) genes that are predicted to encode components of a Type II secretion system (T2SS, Fig. S5) genes. This genome neighborhood structure was largely conserved across the Betaproteobacteria (Figs. S5A–C) and Gammaproteobacteria (Figs. S5D, E). In Betaproteobacteria variation in *gsp* cluster composition occurred with *Paracidovorax anthurii* and *Paracidovorax konjaci* (*gspC* absent, Fig. S5A), two *Simplicispira* genera (*gsp* cluster is located on the opposite strand and downstream of *npdA,* Fig. S5B), *Diaphorobacter oryzae* (*gspHI* absent, Fig. S5B), *Tepidimonas alkaliphilus* (*gspH* absent, Fig. S5C), *Xylophilus* sp. (*gspGHI* absent, Fig. S5C) and *Thauera butanivorans* (an additional gene between *gsp* cluster and *npdA,* Fig. S5C). In the Gammaproteobacteria, two genera lacked a number of *gsp* genes: *Thiothrix,* which had only three *gsp* genes (*gspEFGHIJK* were absent, Fig. S5E) and *Nitrosococcus,* which lacked *gspEFGHI* (Fig. S5E). Isolates identified as *Candidatus Nitrosoglobus terrae* and as unclassified *Chromatiacea* also lacked *gspEFGHI* (Fig. S5E).

### Cryo-EM of additional species of *Acidovorax* and of other Pseudomonadota

Cryo-EM was used to examine cells of 16 cultures (Table 1) identified by the bioinformatics survey as possessing an *npdA* ortholog (Tables 2 and 3). A bead-like, S-layer structure similar to that of *P. citrulli* (Fig. 1a) was clearly visible in most cases (Figs. S6A–P). Two notable exceptions were *P. valerianellae* and *S. psychrophilia*. For *P. valerianellae*, the cell envelope was a complex structure; the outermost layer was relatively thick, and appeared to be multi-layered with granular texture but lacked a clearly resolved bead-like S-layer (Fig. S6I). In *S. psychrophilia*, there were two S-layers that were apparently composed of dissimilar SLP. The S-layer closest to the outer membrane (‘S-layer 1’, Fig. S6L) was composed of subunits that had an appearance similar to that of *P. citrulli* (Fig. 1a) while the SLP subunits of the outermost S-layer (‘S-layer 2’, Fig. S6L) were smaller molecules.

## Discussion

### *Paracidovorax citrulli* AAC00-1 possesses an S-layer composed of NpdA

The present report demonstrated that the locus AAVE_RS20655 in the *P. citrulli* AAC00-1 genome encodes an SLP. This locus is orthologous to DELCS14_RS25720 in *D. acidovorans* Cs1-4, which was established to encode the SLP designated NpdA [10]. Thus, we propose use of *npdA* as the gene name for locus AAVE_RS20655 in *P. citrulli* AAC00-1, as well as for the orthologs in all other *P. citrulli* strains (Table S1) and other genera (Tables 2 and 3).

### Phenotypic effects of the *P. citrulli* AAC00-1 S-layer

The presence of S-layers can significantly impact the physicochemical characteristics of bacterial cells that affect their interaction with environmental surfaces. Alteration of *P. citrulli* AAC00-1 cell surface properties by its S-layer was indicated by the microtest plate experiment in which the WT formed significantly less biofilm than did the Δ*npdA* mutant. The difference between the cell types at least in part indicated that the WT cell surface had a lower binding affinity for the mixed charge surface of the microplate than did that of the Δ*npdA* mutant. The exact physicochemical characteristics that the S-layer imparts on the cell which impact surficial interactions relevant to the growth and survival of *P. citrulli* in its natural habitat (i.e. various plant tissues) remain to be determined.

The results of the melon inoculation experiment indicated that the S-layer was not essential for virulence in *P. citrulli* AAC00-1 when the pathogen was introduced directly into the fruit. There was no significant difference in the extent of disease occurring in detached melon fruits inoculated with the WT vs. fruits inoculated with the Δ*npdA* mutant, as would be expected if the S-layer did contribute an essential function. Examples of S-layers that are essential for a pathogen’s virulence are VapA in *Aeromonas salmonicida* [27] and TfsA and TfsB in *Tannerella forsythia* [28]. However, an S-layer can also be of minor importance to a bacterial pathogen’s virulence, as in the case of the S-layer (AhsA) of *Aeromonas hydrophila* [29]. There is a vast spectrum of plant-microbe interactions that may occur during pathogenesis, and it is possible that the *P. citrulli* S-layer could play an important role in some part of the process. Thus, discerning the possible roles of the *P. citrulli* S-layer in its pathogenesis remains to be the subject of a focused study. Nevertheless, the present work lays the foundation for such future work with *P. citrulli* as it established an S-layer is formed by this bacterium and provided empirical identification of NpdA as the constituent SLP.

Besides (or in addition to) a direct contribution to virulence via plant-microbe interactions, the S-layer may enhance environmental fitness of *P. citrulli* in dimensions of its microbial ecology. For example, the S-layer could provide protection against predation by *Bdellovibrio* and *Bdellovibrio *-like organisms that it may encounter. Evidence for that function was provided by Aharon and coworkers, who screened libraries of *P. citrulli* M6 mini-Tn*5* mutants for sensitivity to predation by *Bdellovibrio bacteriovorus* [30]. One mutant with enhanced susceptibility to predation had a mini-Tn*5* insertion in locus APS58_1147, which is orthologous to *npdA* in *P. citrulli* AAC00-1 (Table S1). In *P. citrulli* M6, disruption of APS58_1147 by mini-Tn*5* would have eliminated the production of NpdA and the S-layer it creates. The heightened sensitivity of the putative Δ*npdA* mutant generated in *P. citrulli* M6 by Aharon and coworkers can be inferred to demonstrate that the S-layer provided an effective barrier to penetration by *B. bacteriovorus *. The ability of S-layers to provide protection against *Bdellovibrio* predation has been demonstrated in other Pseudomonadota [31, 32]. Moreover, for phytopathogenic Pseudomonadota, *Bdellovibrio* predation can significantly reduce fitness in disease establishment [33]. The findings of the present study build on those of [30] and collectively support the conclusion that in *P. citrulli* M6 the S-layer provides protection from *B. bacteriovorus* predation, which could be a factor enhancing its survival and success as a pathogen in its natural environment.

### Nanopod formation by *P. citrulli* AAC00-1

An intrinsic feature of the S-layer in *P. citrulli* AAC00-1 is that it enables formation of nanopods. These structures were readily observed in WT batch culture fluids as detached fragments but were not observed in culture fluids of the *npdA* mutant. This pattern of nanopod formation by *P. citrulli* AAC00-1 follows that documented in prior work with *D. acidovorans* Cs1-4, wherein Δ*npdA* mutants lacking the S-layer no longer produced nanopods but continued to secrete membrane vesicles [10, 11]. The biofilm imaging of *P. citrulli* AAC00-1 revealed that intact nanopods had morphologies like those documented in *D. acidovorans* Cs1-4 via 3D SEM of thick (1250–1500 nm) sections of biofilm [12]. The 3D SEM imaging of *D. acidovorans* Cs1-4 [12] revealed the full structure of nanopods and showed that these were pleomorphic, elongated blocky structures. This data along with cryo-EM imaging of *D. acidovorans* Cs1-4 nanopods [10] indicated that nanopods observed in wet mounts of culture fluids were truncated fragments of the intact structures seen in thin sections of biofilms.

The biofilm imaging of *P. citrulli* AAC00-1 was also consistent with 3D SEM data of *D. acidovorans* Cs1-4 [12] in that it revealed the potential for nanopods to bridge multiple cells and thus potentially create intercellular networks. The 3D SEM of *D. acidovorans* Cs1-4 unequivocally established that nanopods formed structures contiguous with S-layers of neighbouring cells, and also revealed openings into cells through the S-layers at the points of contact (Figshare movie: https://dx.doi.org/10.6084/m9.figshare.3395176.v1). For *P. citrulli* AAC00-1, 3D SEM would be needed to visualize the full structure of nanopods including portals at cell junctions. Nevertheless, the information obtained from the present imaging of *P. citrulli* thin sections was sufficient to establish the existence of intercellular nanopod bridges.

Linear nanopod-like fragments have also been observed by cryo-EM in culture fluids of the Alphaproteobaterium *Caulobacter crescentus *, thus providing evidence that nanopods are potentially formed by a variety Pseudomonadota [34]. The present imaging of nanopod-like structures in *P. citrulli* AAC00-1 biofilms along with the previous 3D SEM data of nanopods in *D. acidovorans* Cs1-4 biofilms provides impetus for future exploration of S-layers as mediators of ‘connexons’. The data obtained so far has established that nanopods create physical connections between cells. It remains to be determined if the OMV contained in nanopods can consequently mediate the intercellular sharing of resources. If such sharing does occur, nanopods would represent a system for conserving resources within a closed community defined by S-layer interactions, thereby overcoming the potential loss of OMV-transmitted resources to ‘cheaters’ [35]. Such a system would constitute a new type of prokaryotic multicellularity.

### Distribution of *npdA* orthologs

Orthologs of *npdA* occur in wide-range of species that are distributed across the Betaproteobacteria and Gammaproteobacteria subdivisions. Another recently described SLP from *Pseudoaltermonas tunicata*, termed Slr4, displays a similar distribution pattern across multiple subdivisions with orthologs identified in species of Alphaproteobacteria and Gammaproteobacteria [36]. A difference between these two SLPs is that NpdA is mostly associated with terrestrial/aquatic species while species possessing Slr4 are primarily of marine origin. Also, NpdA appears to be distributed across a broader diversity of families and genera than is Slr4. Thus, NpdA and Slr4 are examples of SLPs that have broad phylogenetic distributions.

The *npdA* orthologs were particularly common in the Betaproteobacteria genera *Acidovorax* and *Delftia *. The presence of an S-layer was documented by TEM for three species in these genera decades ago: *Pseudomonas */*Comamonas acidovorans,* (now *Delftia acidovorans *) [37–39]; *Pseudomonas delafieldii* (now *Acidovorax delafieldlii*) [38]; *Pseudomonas avenae* (now *Paracidovorax avenae*) [40]. However, the identity of the SLP that comprised the S-layer in those species was undetermined. The present work has identified NpdA as the cognate SLP for these organisms.

For most of the other Betaproteobacteria and Gammaproteobacteria listed in Tables 2 and 3, the present study is the first identification of a putative SLP, and consequently the potential presence of an S-layer in these organisms. Two exceptions were *S. psychrophila* and species of *Nitrosococcus *. For *S. psychrophila* (formerly *Aquaspirillum psychrophila* [41]), prior studies with other *Simplicispira* species (*S. serpens*, *S. sinuosum*) revealed a unique double S-layer [42, 43], however the SLP comprising each of these S-layers has not been identified. The marine ammonia-oxidizers of the genus *Nitrosococcus* (previously *Nitrosocystis,* [44]) identified with *npdA* were among the first bacteria in which S-layers were originally observed by freeze-etching and TEM [45, 46]. While the presence of an S-layer in *Nitrosococcus* has been known for more than fifty years, the constituent SLP has not been identified; the present report provides evidence of an NpdA ortholog as a candidate for that SLP.

### Cryo-EM imaging of S-layers in other Pseudomonadota

Cryo-EM imaging provided the first views of S-layers in most of the selected cultures. Based on the combination of the imaging data and the bioinformatics identification of *npdA* orthologs in these cultures, we hypothesize that these S-layers are composed of NpdA. However, unambiguous identification of these SLPs will require detailed genetic, biochemical and biophysical analyses such as that done in the present study with *P. citrulli* AAC00-1. The current report nevertheless provides an important first step in guiding these future investigations.

The cryo-EM imaging also provided unexpected insights into other aspects of cell envelope biology. For example, while double S-layers have been identified in other species of *Simplicispira *, the cryo-EM imaging presented here documented these structures for the first time in *S. psychrophile*. In *R. cellulosilytica *, the large extracellular vesicles observed appeared to be composed of an exterior layer, made of outer-membrane and S-layer, that encapsulated an interior vesicle derived from the inner-membrane. These structures, termed outer-inner membrane vesicles, have been observed in other Pseudomonadota and have the potential to transmit cytoplasmic as well as periplasmic materials [35]. Most intriguing was the unusual cell envelope of *P. valerianellae*, which displayed three distinct structures. Two of these were identifiable as the inner- and outer-membranes, the latter lacking the beaded structure typical of the NpdAS-layer. The third, outermost object was an unknown multi-layer structure, which in some respects resembled cysts formed by the genus *Azotobacter* [47, 48]. *P. valerianellae* was first described only relatively recently via its identification as the causal agent of spot disease on lamb’s lettuce [49]. As such, there have been relatively few investigations concerning its cell biology; future research should seek to identify this structure.

### Structure and conservation of the *npdA* genomic neighborhood

The structure of the *npdA* genome neighborhood is similar to that of other SLP genes in the physical linkage of the SLP-encoding locus with a group of genes encoding a secretion system. For example, *rsaA* is clustered with genes encoding a Type I secretion system in *Caulobacter crescentus* [50]. In *Aeromonas salmonicida *, the SLP-encoding *vapA* gene adjoins a T2SS cluster [29]. Similarly, the *slr4* gene of *P. tunicata* is located immediately upstream of a T2SS cluster [36]. The secretion system clustered with SLP genes in *A. salmonicida* is essential for S-layer formation, and is considered dedicated to SLP export [29]. Future studies focused on mutational analysis of the T2SS clustered with *npdA* could determine if this system is similarly essential for NpdA section and S-layer formation.

The *P. citrulli* genome contains two gsp clusters (identified as gsp1 and gsp2) and *npdA* is associated with the cluster identified as gsp1 [51]. Johnson and coworkers explored the potential contribution of T2SS to *P. citrulli* AAC00-1 virulence by deleting *gspG* from both gsp clusters [51]. The resultant mutant was presumably disabled in all T2SS activity, which would have prevented secretion of NpdA (and consequently eliminated S-layer formation) as well as a secretion of a wide range of other proteins (mainly enzymes) that could function as virulence factors. The Δ*gspG* mutant was diminished in several virulence-related traits including decomposition of some plant tissues, colonization of watermelon seed, and seed to seedling dissemination. Because loss of T2SS activity would affect a wide range of secreted molecules, the specific contribution of the S-layer, if any, to these phenotypes remains to be determined.

## Conclusions

This work established that an S-layer composed of the SLP designated NpdA is a core characteristic of the *P. citrulli* cell envelope. *Paracidovirax citrulli* is the second taxon for which *npdA* has been empirically shown to encode an SLP. Bioinformatics analysis of *npdA* ortholog distribution and cryo-EM imaging revealed a potentially broad distribution of NpdA-based S-layers across a diverse range of Betaproteobacteria and Gammaproteobacteria, suggesting it is an important new family of SLP. The present study in combination with work done by Aharon and coworkers [30] indicated the NpdA S-layer can protect *P. citrulli* from predation by *Bdellovibrio *. However, the potential function of the S-layer in pathogenesis remains to be systematically explored for *P. citrulli,* as well as for the other species of plant pathogenic *Paracidovorax* and *Paenacidovorax* all of which possess *npdA*. Lastly, this study showed that S-layer production enabled *P. citrulli* to form cell-bridging nanopods in biofilms, a growth state which it likely exhibits naturally in the colonization of plant tissues. Thus, an important goal of future research would be to determine if nanopods enable direct, intercellular movement of materials. If they do, the biological implications would be significant not only for *P. citrulli* but potentially for S-layer-forming Pseudomonadota more broadly.

## Supplementary Data

Supplementary material 1
