# Supplementary material for: Identification of NpdA as the protein forming the surface layer in Paracidovorax citrulli and evidence of its occurrence as a surface layer protein in diverse genera of the Betaproteobacteria and Gammaproteobacteria
Source: Access Microbiol. 2023 Dec 11;5(12):000685.v3. doi: 10.1099/acmi.0.000685.v3 (PMC10765051; doi:10.1099/acmi.0.000685.v3)
Supplement: Supplementary material 1 [file acmi-5-685.v3-s001.pdf]

Table S1. Summary of npdA ortholog loci in strains of Paracidovorax citrulli

Strain	Protein	Annotation	Gene	Prior Locus tag	Ref seq
AAC00-1	WP_011797163	Hypothetical Protein	AAVE_RS20655	Aave_4148	NC_008752.1
KACC17005	WP_011797163	Hypothetical Protein	QRO08_RS03520	QRO08_03520	NZ_CP127363.1
KACC18784	WP_011797163	Hypothetical Protein	QRO10_RS24485	QRO10_24485	NZ_CP127360.1
KACC17913	WP_011797163	Hypothetical Protein	QRO12_RS21350	QRO12_21350	NZ_CP127362.1
KACC18782	WP_011797163	Hypothetical Protein	QRO09_RS24290	QRO09_24290	NZ_CP127361.1
NWB SC074	WP_011797163	Hypothetical Protein	FRC75_RS24205	FRC75_24295	NZ_CP042302.1
NWB SC196	WP_017437879	Hypothetical Protein	FRC97_RS01750	FRC97_01745	NZ_CP042323.1
M6	WP_017437879	Hypothetical Protein	APS58_RS05735	APS58_1147	NZ_CP029373.1
HPP21-9-4B	WP_017437879	Hypothetical Protein	LKW30_RS00375	None	NZ_CP086060.1
HPP21-3-3B	WP_017437879	Hypothetical Protein	LKW27_RS03475	LKW27_03485	NZ_CP086023.1
Pslb65	WP_017437879	Hypothetical Protein	TY99_RS14760	None	NZ_JYHM01000022.1
KACC17001	WP_074686552	Cell Surface Protein	QRO11_RS03495	QRO11_03495	NZ_CP127364.1
NWB SC107	WP_074686552	Cell Surface Protein	FRC90_RS18175	FRC90_18150	NZ_CP042303.1
DSM 17060	WP_074686552	Cell Surface Protein	BLR99_RS00660	SAMN04489709_101135	NZ_FNEY01000001.1
Tw6	WP_046059823	Hypothetical Protein	RU63_RS06380	None	NZ_JXDJ01000021.1

Table S2. List of bacterial strains, plasmids and PCR primers used in the mutagenesis experiments.

Item	Characteristics	Reference/Source
Strains		
Paracidovorax citrulli AC-0001		
WT	Wild type, reference genome sequence NC_008752.1	R. Walcott
ΔnpdA	Deletion of *npdA* (locus tag AAVE_RS20655)	This study
Escherichia coli WM3064	*thrB1*004pro *thi rpsL hsdS lacZ*Δ15 RP4-1360 Δ(*araBAD*)*567* Δ*dapA1341*::[*erm pir*]	W. Metcalf
Plasmids		
pJK100	Allelic-exchange vector	V. Denef
pCM157	Cre expression vector	V. Denef
pEG100	pJK100 containing “npdA UP” and “npdA DOWN” genome fragments	This study
Primers[a]		
npdA Up F1 BglII	**ATCCAGATCT**GGATCTTTGATGCGTCCGAATG (4603182-4603161)	This study
npdA Up R1 NdeI	**AATTCCATATG**GCGCAACTACCACGATTGATTAC (4602662-4602684)	This study
npdA Down F1 SacII	**ATACCGCGG**TAATTCACTGCTGCTACGGCAC (4602662-4602684)	This study
npdA Down R1 SacI	**TATGGAGCTC**GTTCCGGCTTGGCCTTGTAG (4600528-4600547)	This study
npdA External F1	TGGCAGGGCAGTACGAGAAGAC (4603447-4603426)	This study
npdA External R1	GCCGAACTTGCTCTCCACGATG (4600250-4600271)	This study
npdA Internal F1	CAACCAGACCCGTGAAGGCTAC (4602165-4602144)	This study
npdA Internal R1	CATGGAGAACAGCCAGTCGGTC (4601544-4601565)	This study
kanF	ATTGTTGATGCGCTGGCAGT	Denef et al. 2006 (18)
kanR	TCCGGTGAGAATGGCAAAAG	Denef et al. 2006 (18)

[a]Bold letters in primer sequences indicate 5’ extensions used to introduce a restriction site (underlined) for the enzyme indicated in the primer name. Numbers indicate base range in the reference genome sequence.

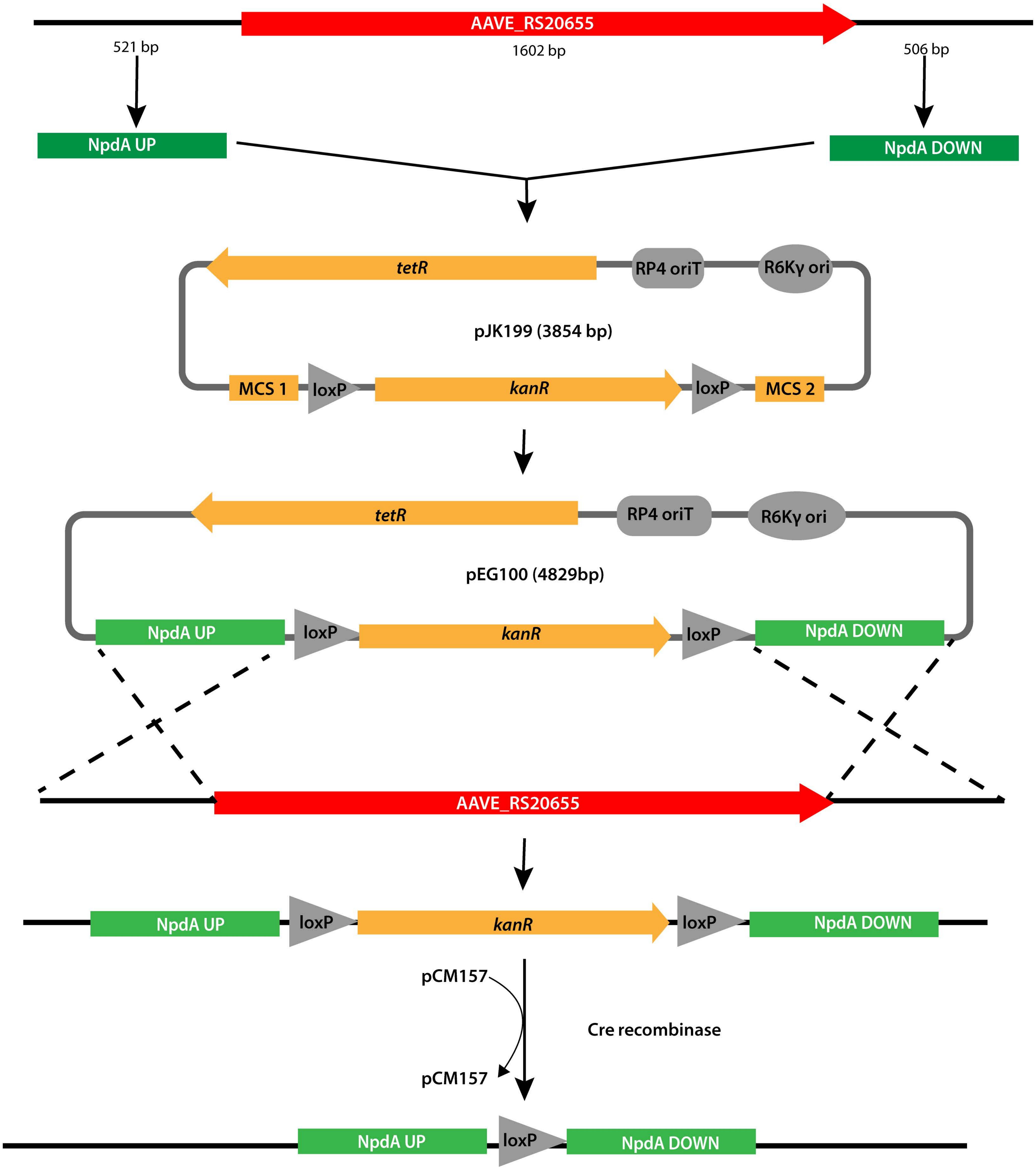

Figure S1. Outline of the allelic exchange approach used to generate the P. citrulli AAC00-1 ΔnpdA mutant.

A

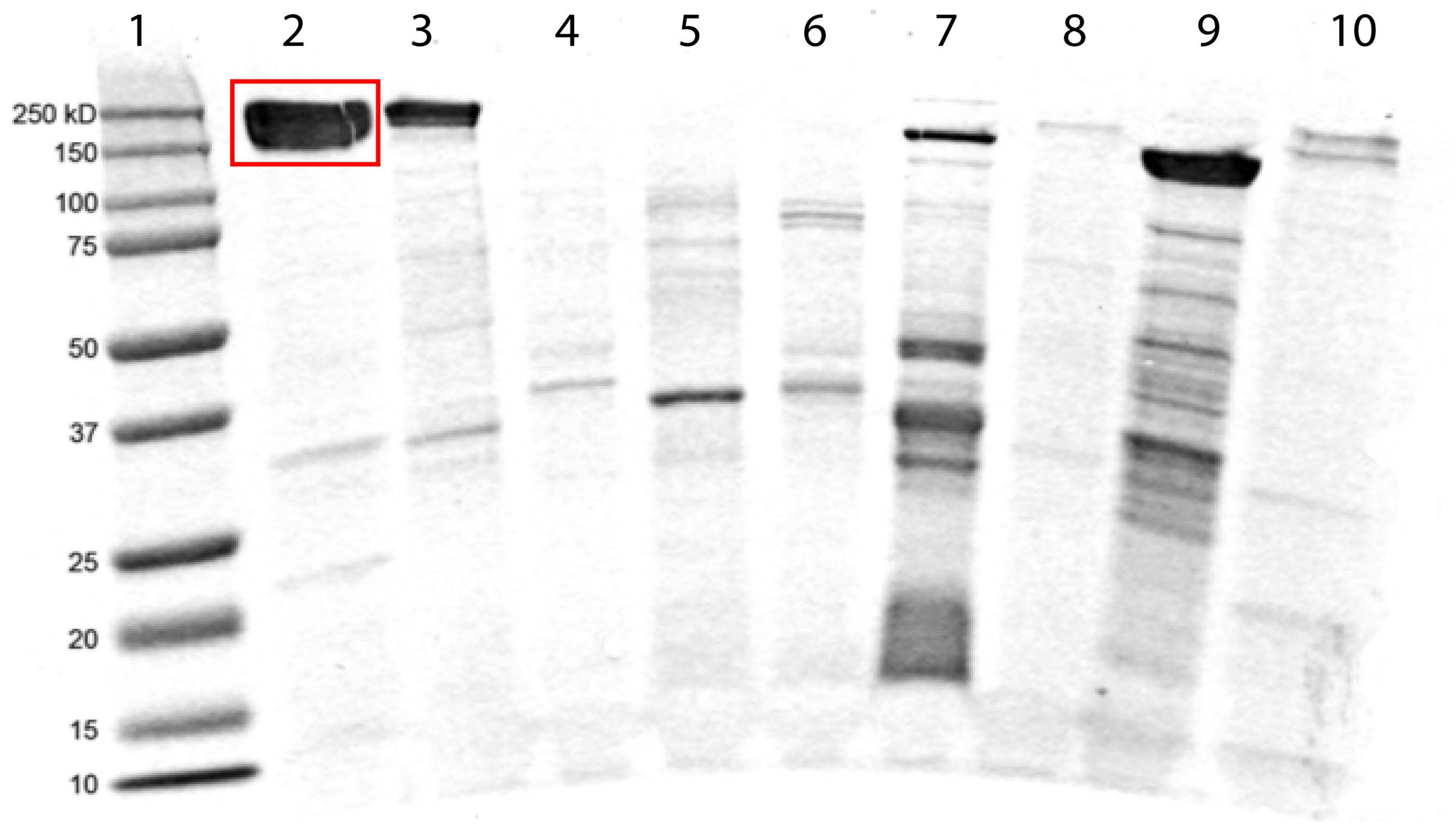

B

>WP_011797163.1 hypothetical protein [Paracidovorax citrulli]
MKKNVLALSIAAMIGGLGFAGAASADVVVGATPLTATNATSLSFAEGGVGHALLVPYFNAQNGNMTVLH
VVNTDTSRGKAVKVRFRGAQNSDDILDFQVFMSPGDVWTAAVTAGSDGVAQLQTADGTCTLPALAKNVP
QRFVTDR**LNQGLATADLANQTR**EGYVEIFNMADIPATLAGSNTTNPLYTAIKHVNGVAPCTSSALNATLQNF
TTPAAVAAAGFDTPTTGLVGDWYIINVAQTTTFAGAATAIR**AENGGAPAVGNFVHFPQMASNAATPDNF**
TADPLFRATNVFNAAGVAVTSPKIAAANYDLPDMSTPYTANGGVAVSPLVQATNLTNALAVTSITNQYATD
ASISAK**TDWLFSMPTR**R**YNVAANYAAANQSPADTSNVRLFTDLNGSGGVADERFNPTNTSLQAVGGAIC**
VNSTGQAFFDREEQTQTAGAVFSPGSVTQTR**FCGETSVLSFASGSVLGASVASQQLTTGAYTNGWSRVDV**
PNNGLGLPILGASFIKLANPLASAGTSGTYGITWPHRFTRPAAQ

Sequence Coverage: 38%
Score: 527
Expect: 6.9e-050
Mascot Search parameters
Fixed modifications: Carbamidomethyl (C) Variable modifications: Deamidated (NQ), Oxidation (M) Cleavage by Trypsin: cuts C-term side of KR unless next residue is P

Figure S2. Panel A: Analysis by SDS-PAGE of surface layer protein (SLP) extracted from *P. citrulli* AAC00-1. Lane 1, Molecular weight marker; Lane 2, extracted SLP; Lanes 3-10, Other protein samples not relevant to the present study. The red box indicates the region of gel excised for analysis by mass spectrometry. Panel B: The amino acid sequence of the predicted gene product of AAVE_RS20655 (WP_011797163) showing peptides identified by mass spectrometry (boldface red) and the predicted signal peptide (boldface blue; SignalP 6, likelihood = 0.9989)

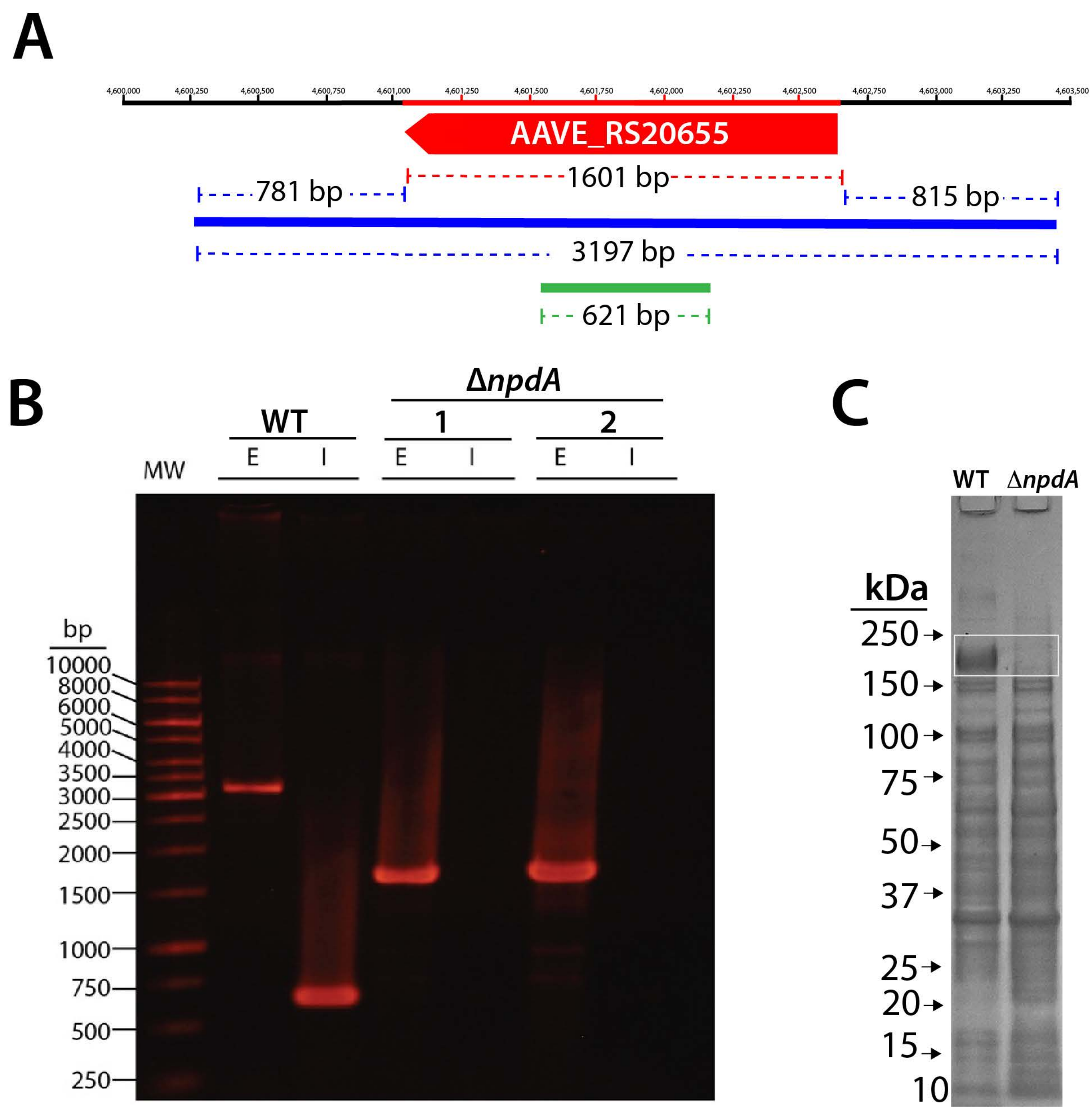

Figure S3. Mutant confirmation by PCR and protein analyses. Panel A: Genome map. The target locus is located at genome positions 4,601,031-4,602,632 in record NC_008752.1. The blue lines indicate PCR products created by primers external (E) to the region targeted for deletion giving a product of 3197 bp in the wild type (WT) and 1596 bp in the Δ*npdA* mutant. The green bar designates the PCR product generated by primers internal (I) to the targeted region giving a 621 bp product only in the WT. Panel B: Agarose gel separation of PCR products amplified from the WT and two clones of the Δ*npdA* mutant. The WT shows both "E" and "I" products of the expected size. The two Δ*npdA* mutant clones gave identical results, having an "E" product of expected size but lacking an "I" product, also as expected. Panel C: Comparative SDS-PAGE analysis of whole cell protein profiles of *P. citrulli* AAC00-1 WT and Δ*npdA* mutant. The band formed by NpdA is indicated by the white box.

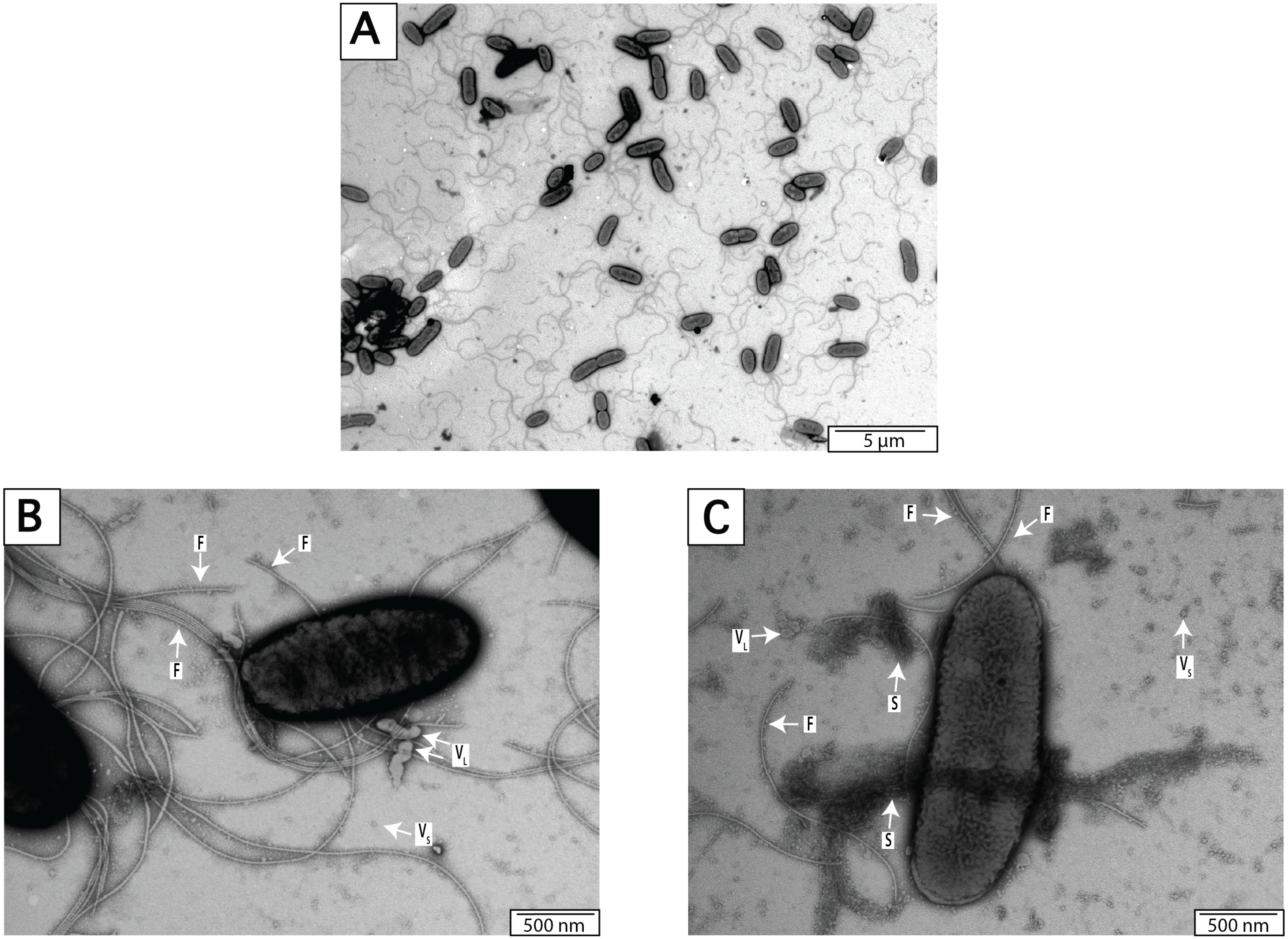

Figure S4. Culture fluids of *P. citrulli* AAC00-1 Δ*npdA* mutant imaged by negative staining and TEM. Panel A: Wide view showing cells and detached flagella. Panels B and C: Magnified views showing details of flagella (F), large, globular vesicles (V_L) and small spherical vesicles (V_S). Areas of Phosphotungstic acid stain accumulation on clumps of extracellular structures are also indicated (S).

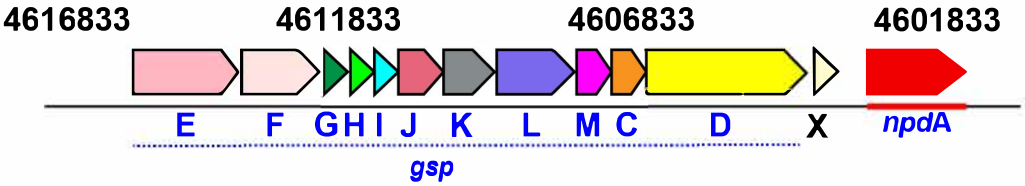

Figure S5A. Illustration of *npdA* neighborhood in the *P. citrulli* AAC00-1 genome showing the associated *gsp* cluster predicted to encode a Type II Secretion System. Blue letters below the illustration indicate *gsp* gene assignment, the black "X" is an ORF with a predicted polypeptide annotated as "Hypothetical protein". Numbers above illustration are the *P. citrulli* AAC00-1 genome coordinates.

Figure S5B-F (Following pages) Genome neighborhoods of *npdA* in Betaproteobacteria (Panels B-D) and Gammaproteobacteria (Panels E-F) showing the associated *gsp* cluster. The genome segments are displayed 5'-3' and aligned on the *npdA* ortholog. The *gsp* genes are color-coded as indicated in the legend to Fig. S5A. Genes predicted to encode non-GSP proteins or hypothetical proteins are colored white.

Figure S5B. Betaproteobacteria: *Acidovorax*/*Paracidvorax* and *Delftia*

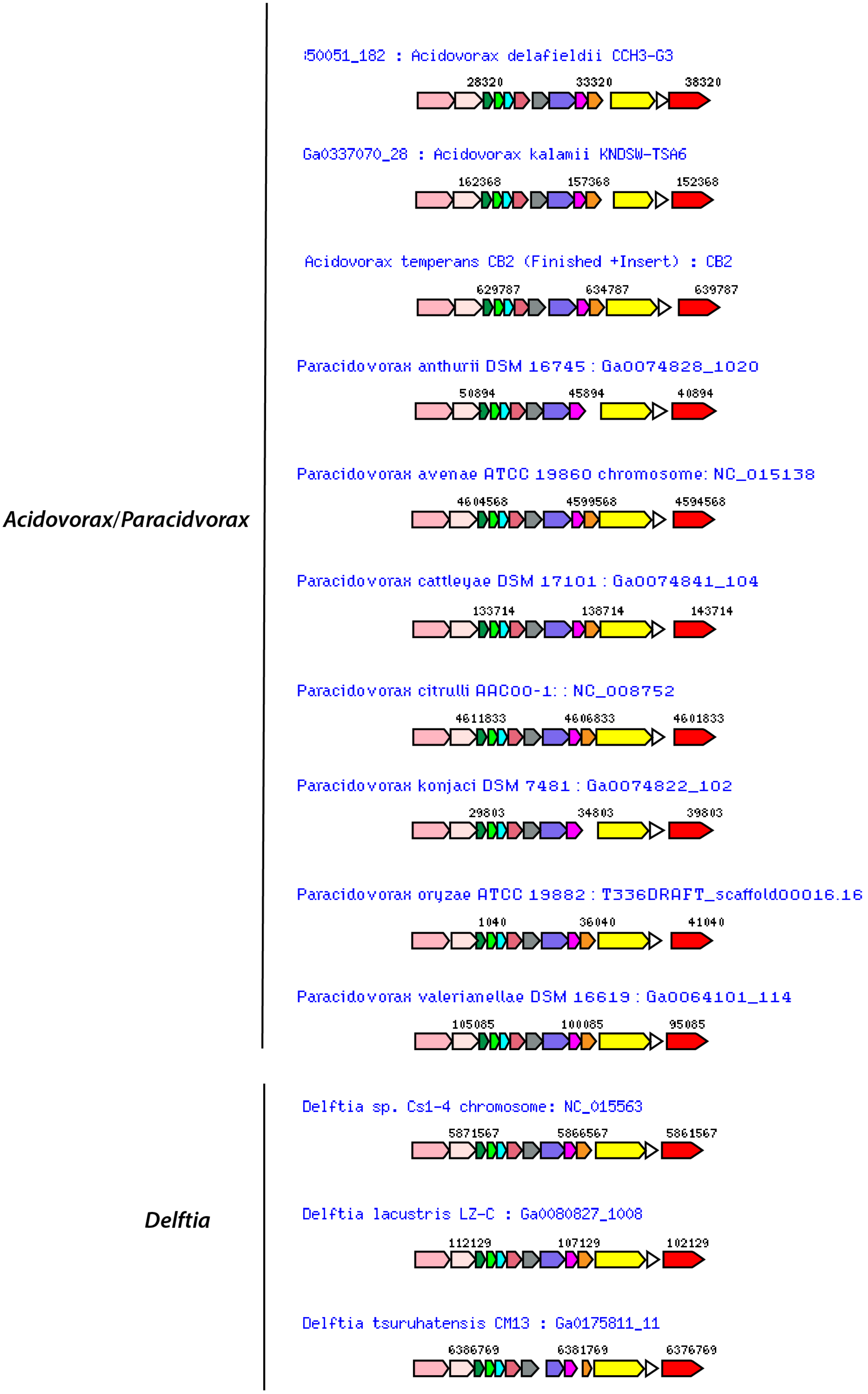

Figure S5C. Betaproteobacteria: Other *Comamondacea* in the Burkholderiales

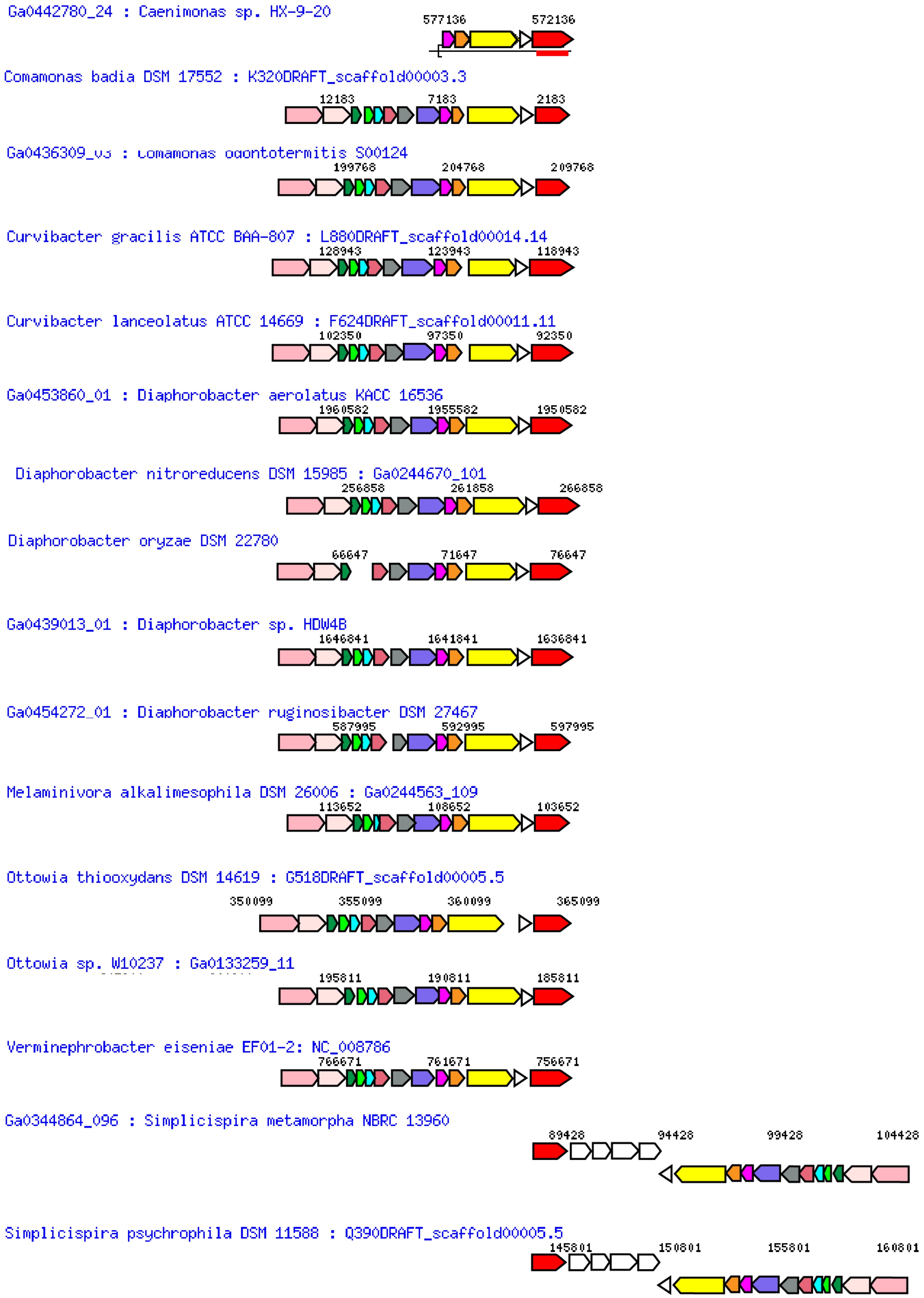

Figure S5D. Betaproteobacteria families Nitrosomonadales, Rhodocyclales and Burkholderiales genera incertae sedis

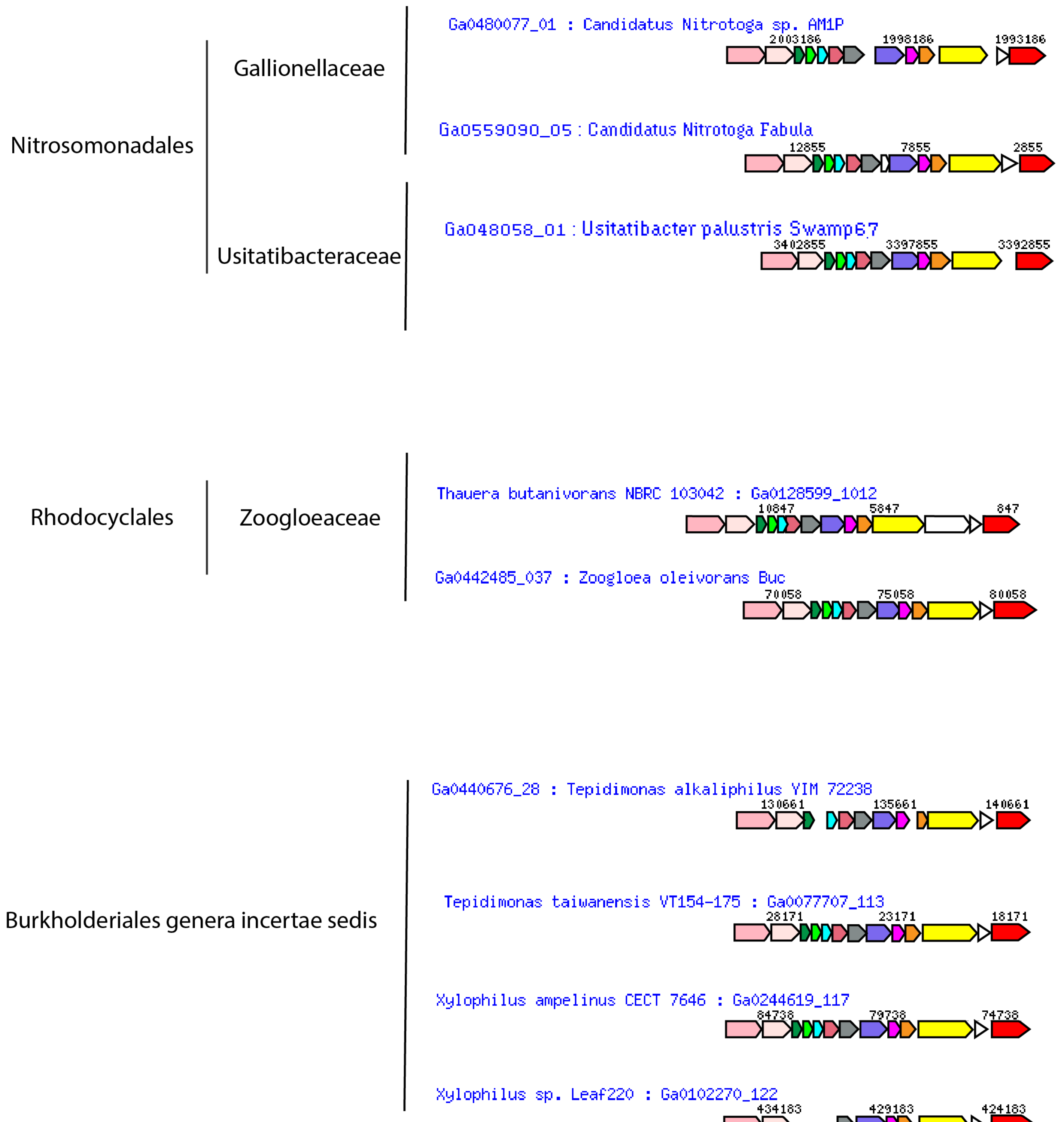

Figure S5E. Gammaproteobacteria orders Cardiobacteriales and Cellvibrionales

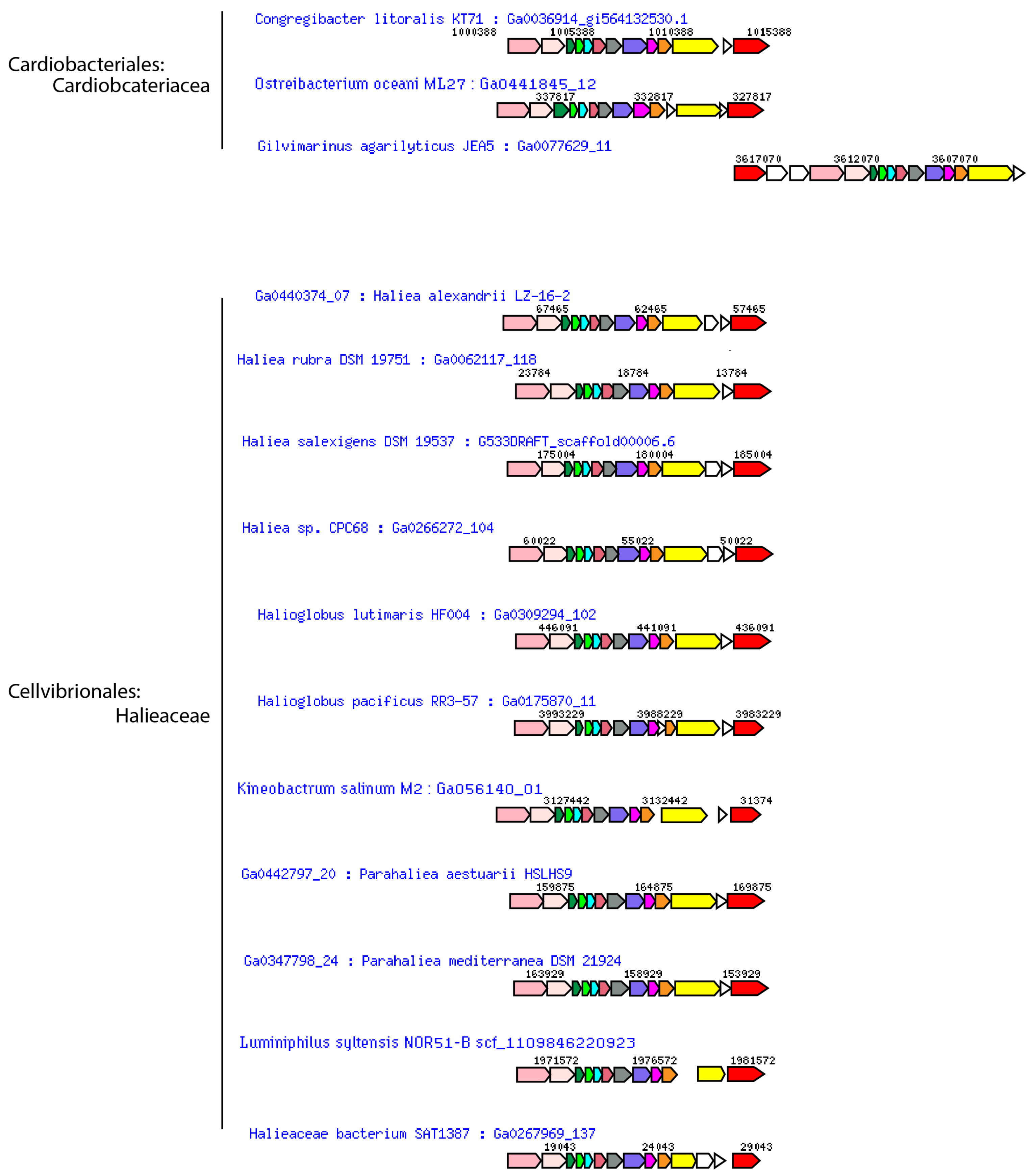

Figure S5F. Gammaproteobacteria orders Chromatiales, Oceansopiralaes, Pseudomonadales, Thiotrochales, Xanthomondales and Gammaproteobacteria incertae sedis

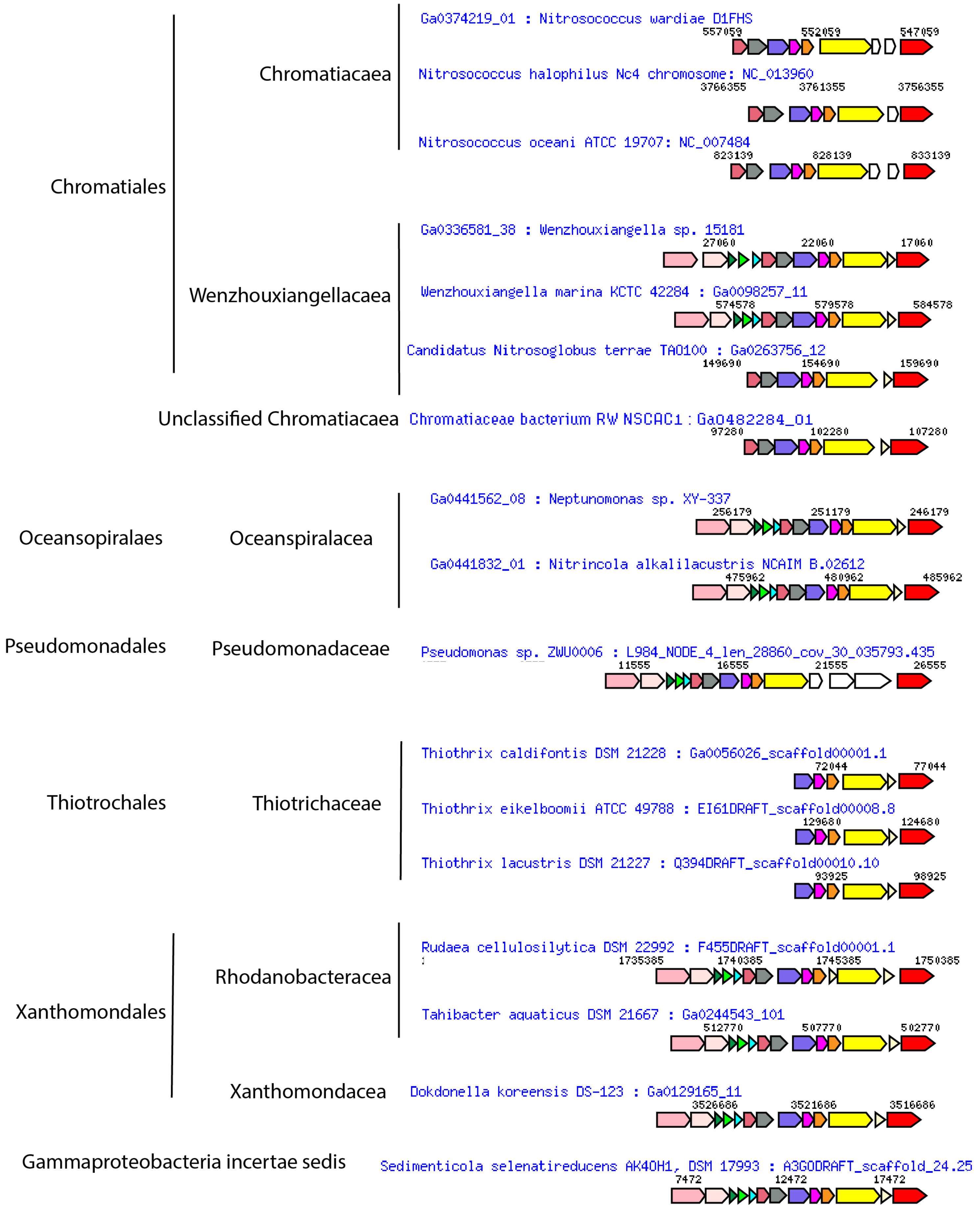

Figure S6. (Following pages) Cryo-electron examination of *Proteobacteria* containing a *npdA* ortholog for an S-layer. Panel A: *Acidovorax delafieldii*, Panel B: *Acidovorax radicis*, Panel C: *Paenacidovorax caeni*, Panel D: *Paracidovorax anthurii,* Panel E: *Paracidovorax avenae*, Panel F: *Paracidovorax cattleyae*, Panel G: *Paracidovorax konjaci*, Panel H: *Paracidovorax oryzae,* Panel I: *Paracidovorax valerianellae,* Panel J: *Delftia acidovrans,* Panel K: *Ottowia thiooxydans*, Panel L: *Simplicispira psychrophilia,* Panel M: *Vermineprobacter eiseniae,* Panel N: *Congregibacter litoralis*, Panel O: *Hailea salexigens*, Panel P: *Rudaea cellulosilytica*. Abbreviations: IM, Inner membrane; OM, Outer membrane; S-layer, Surface layer.

Figure S6A. *Acidovorax delafieldii*

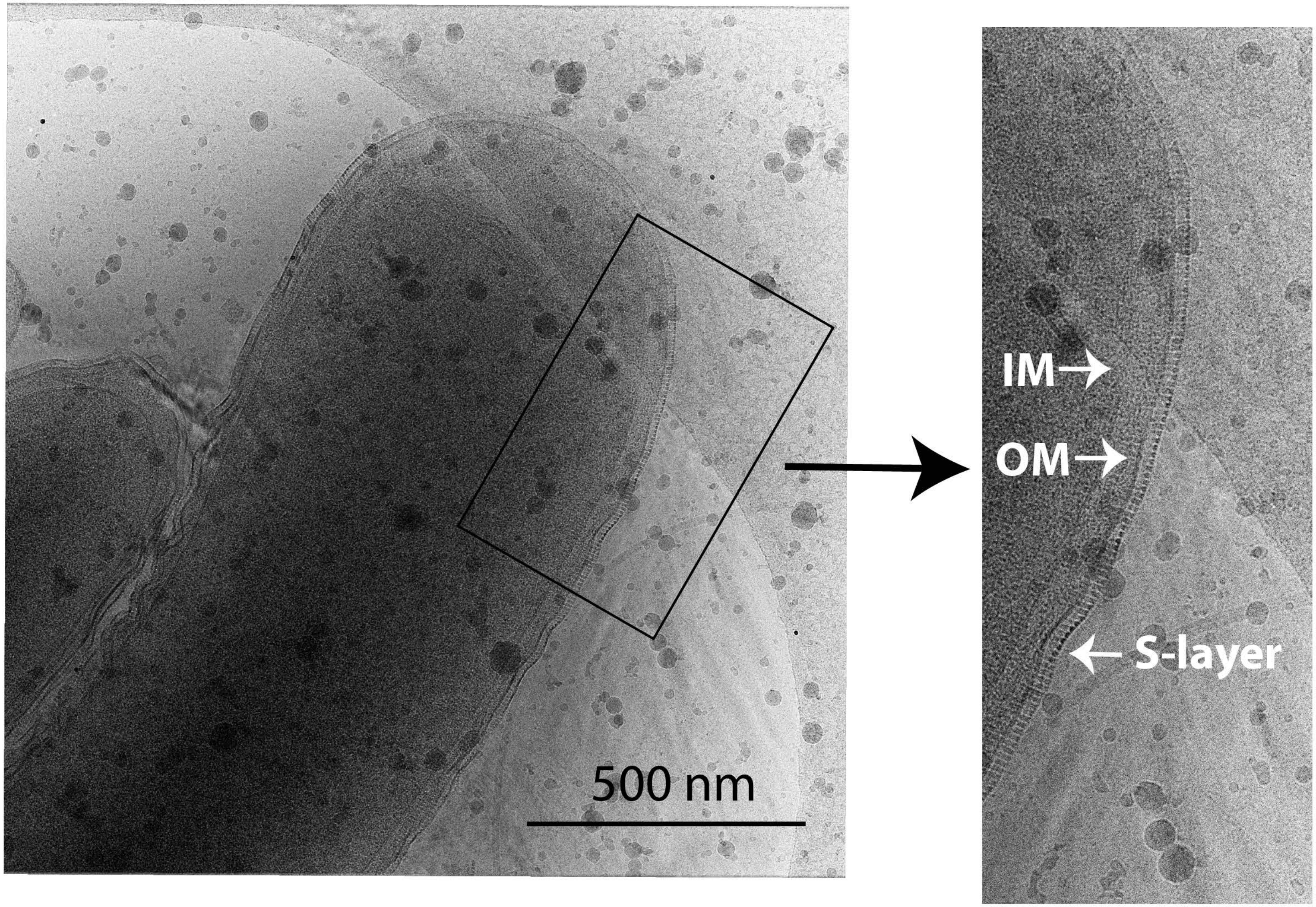

Fig. S6B. ***Acidovorax radicis***

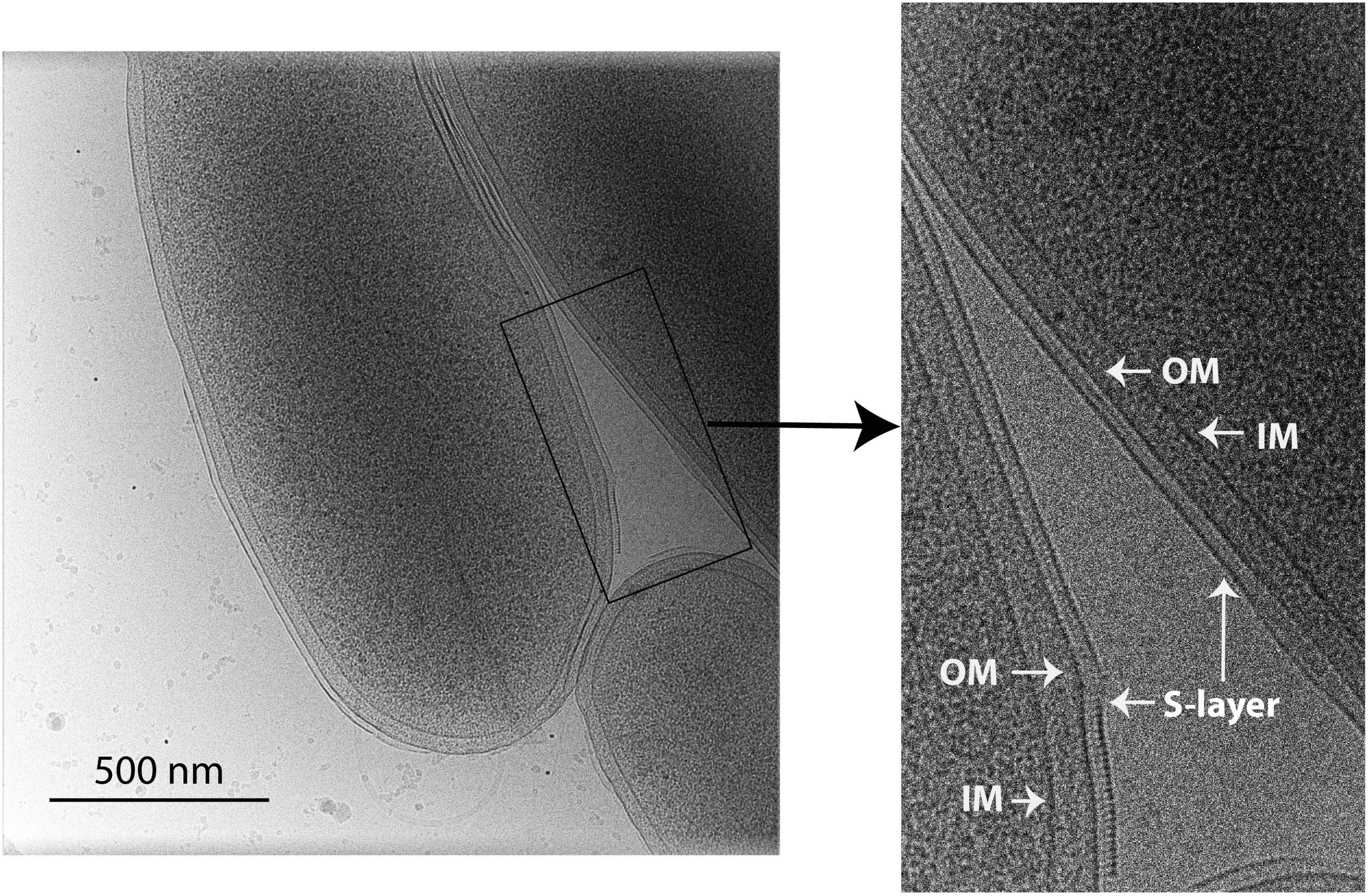

Fig. S6C. ***Paenacidovorax caeni***

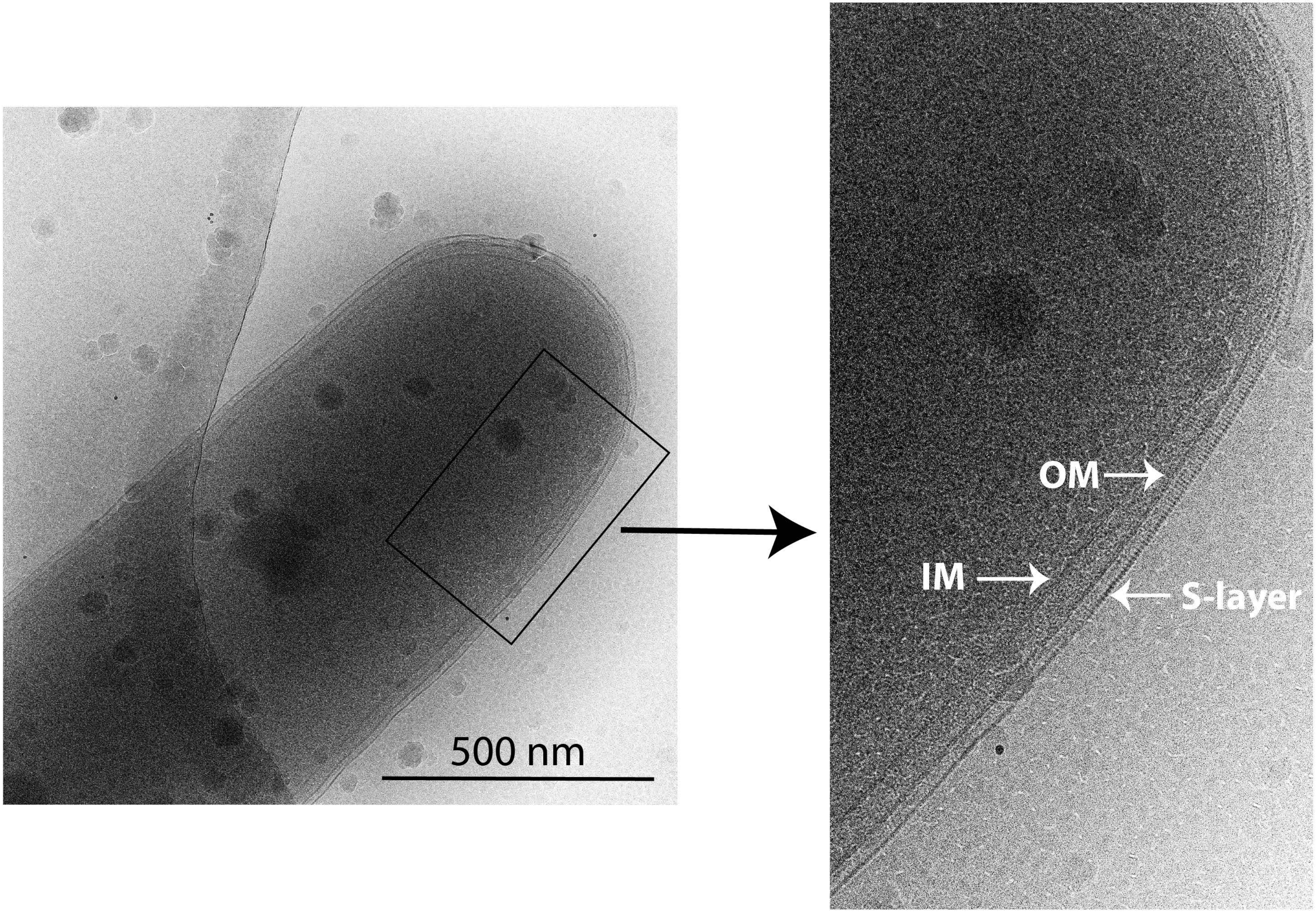

Fig. S6D. ***Paracidovorax anthurii***

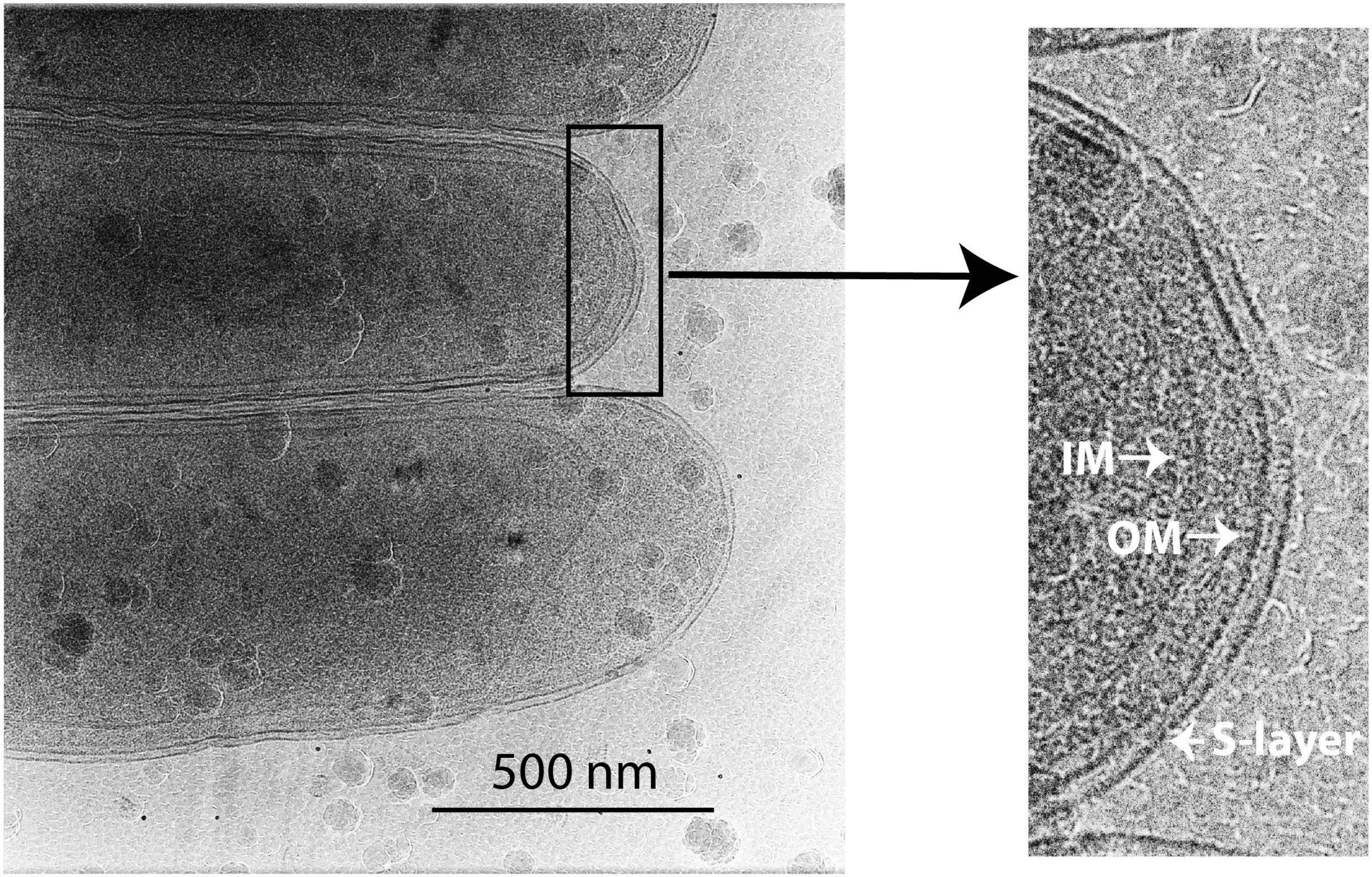

Figure S6E. *Paracidovorax avenue*

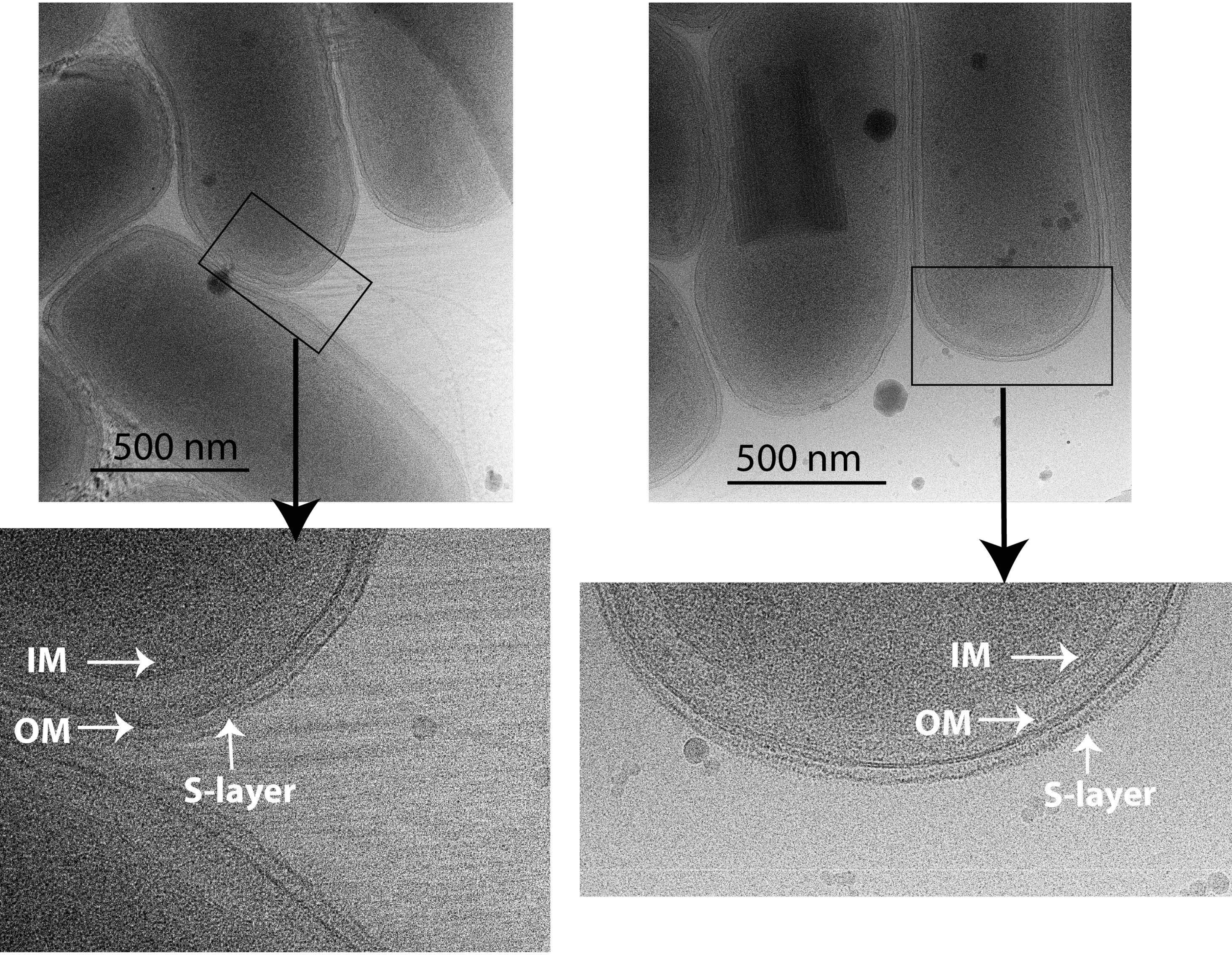

Figure S6F. ***Paracidovorax cattleyae***

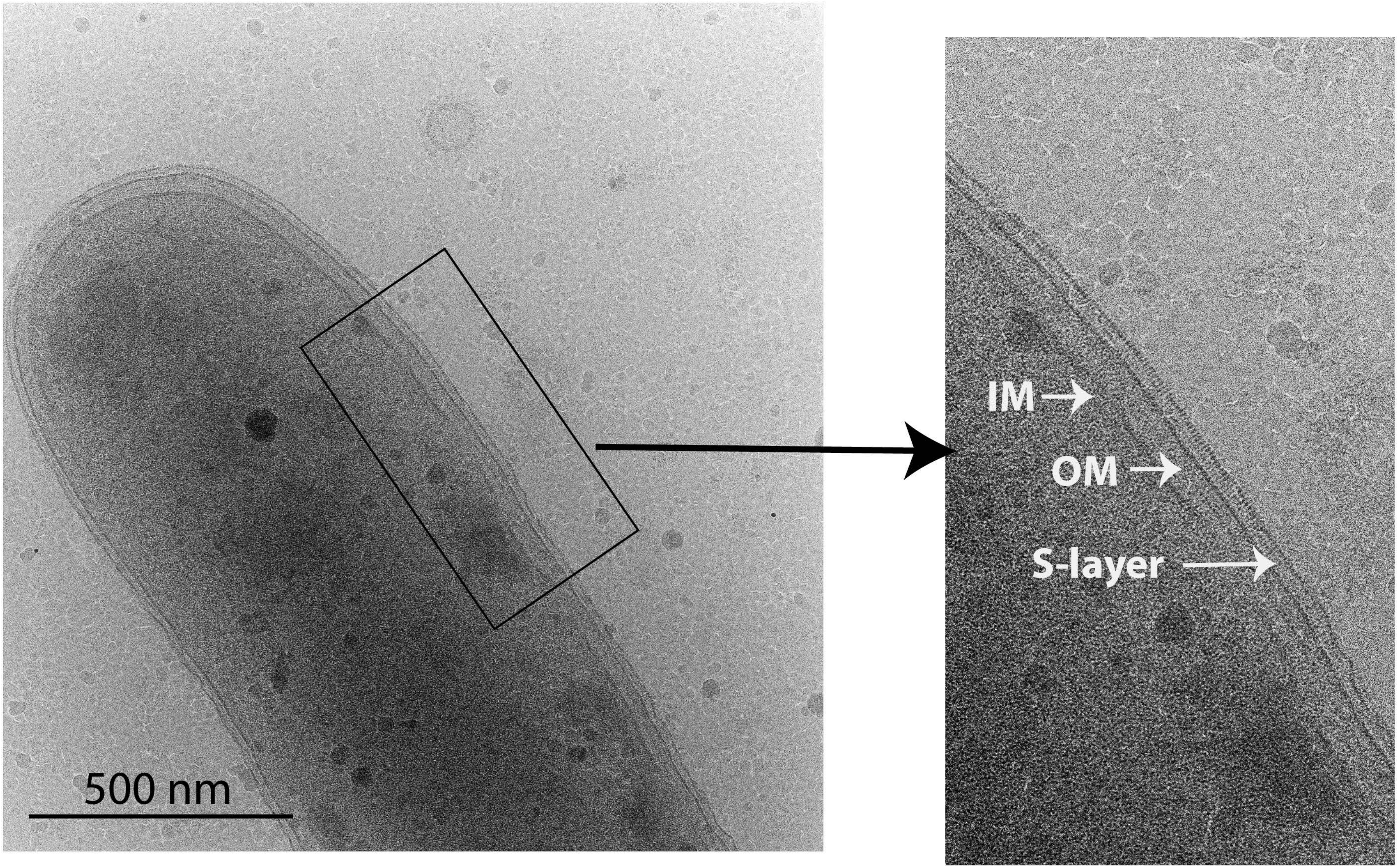

Figure S6G. ***Paracidovorax konjaci***

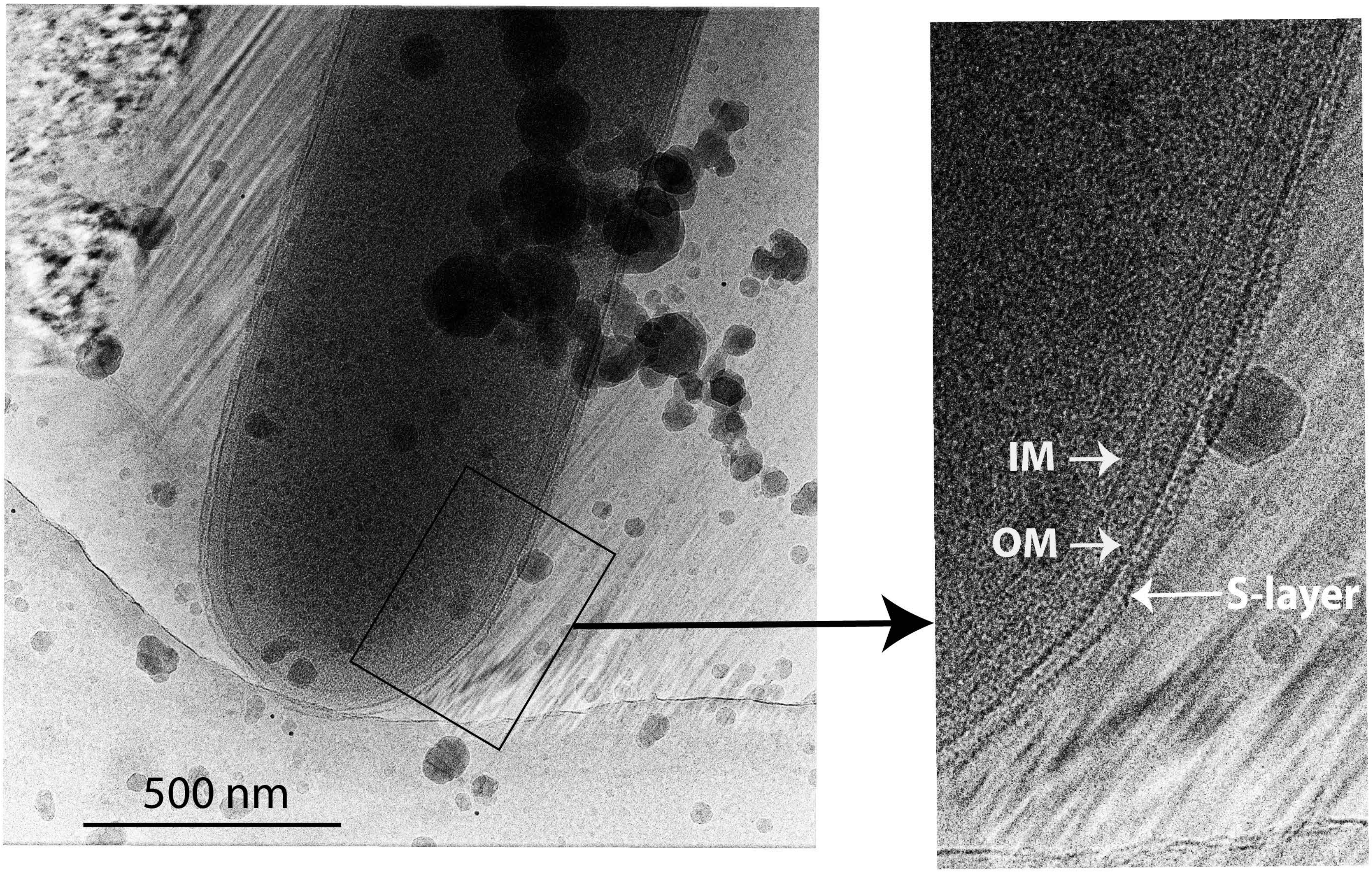

Figure S6H. ***Paracidovorax oryzaea***

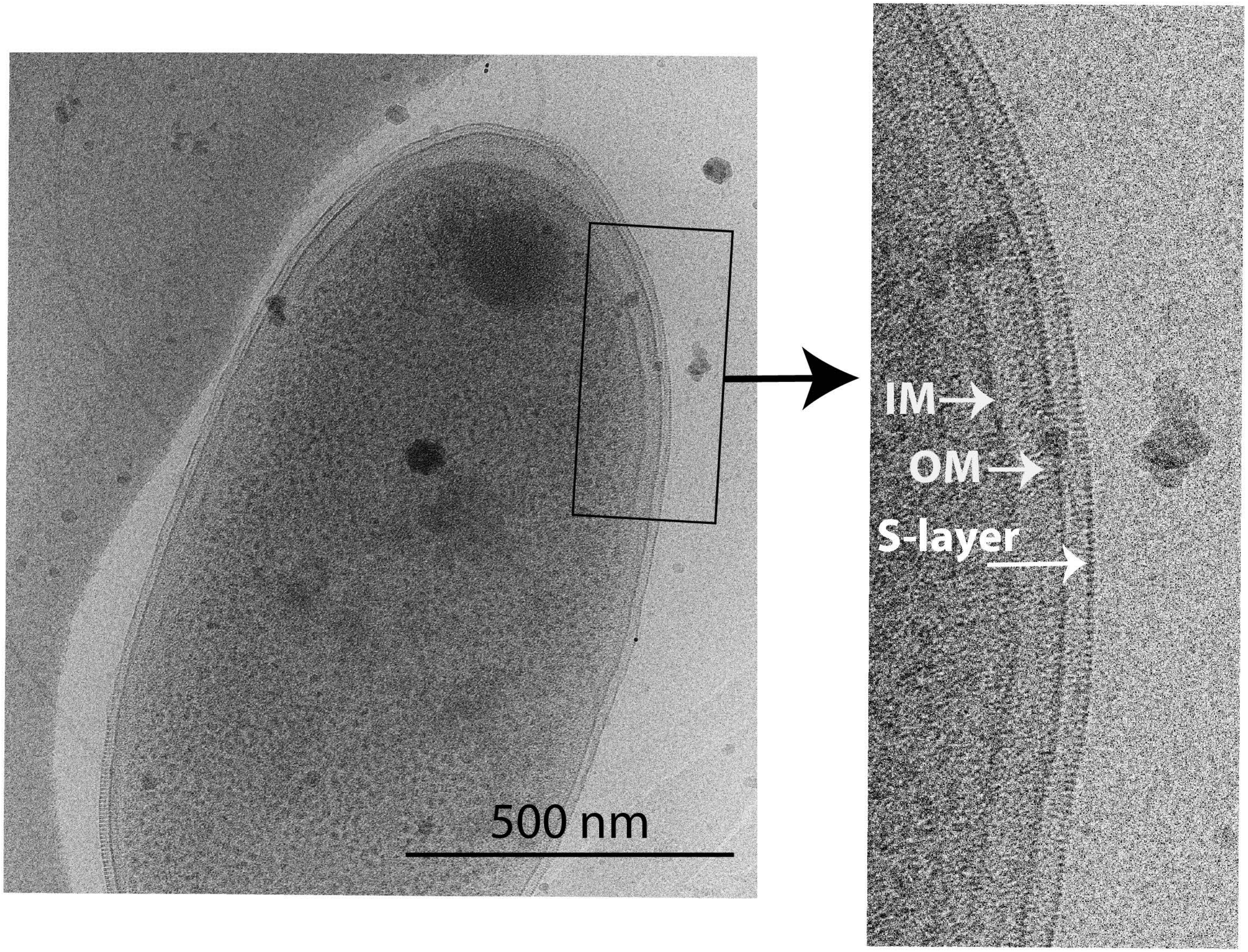

Figure S6I. *Paracidovorax valerianellae*

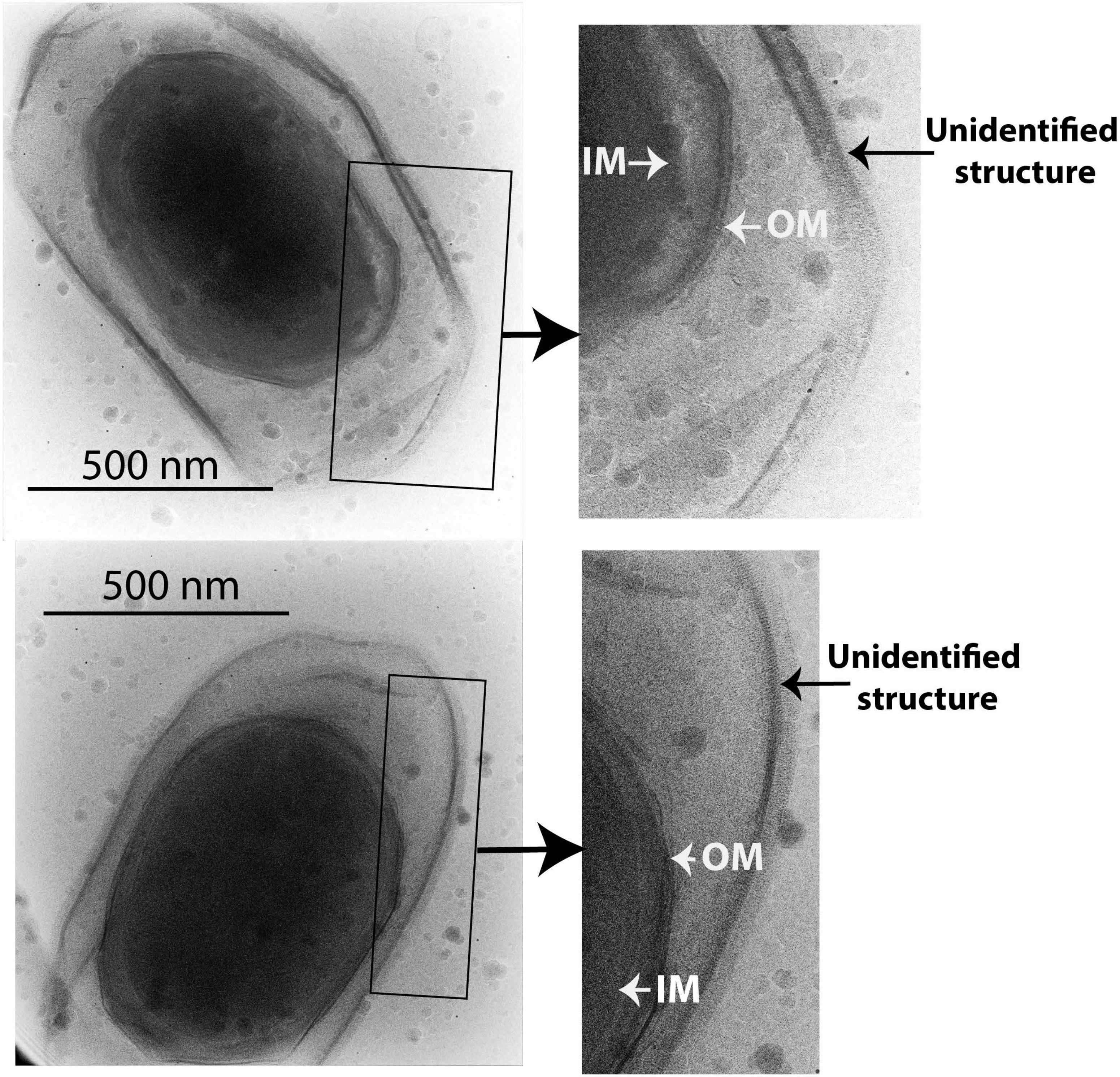

Figure S6J. ***Delftia acidovorans***

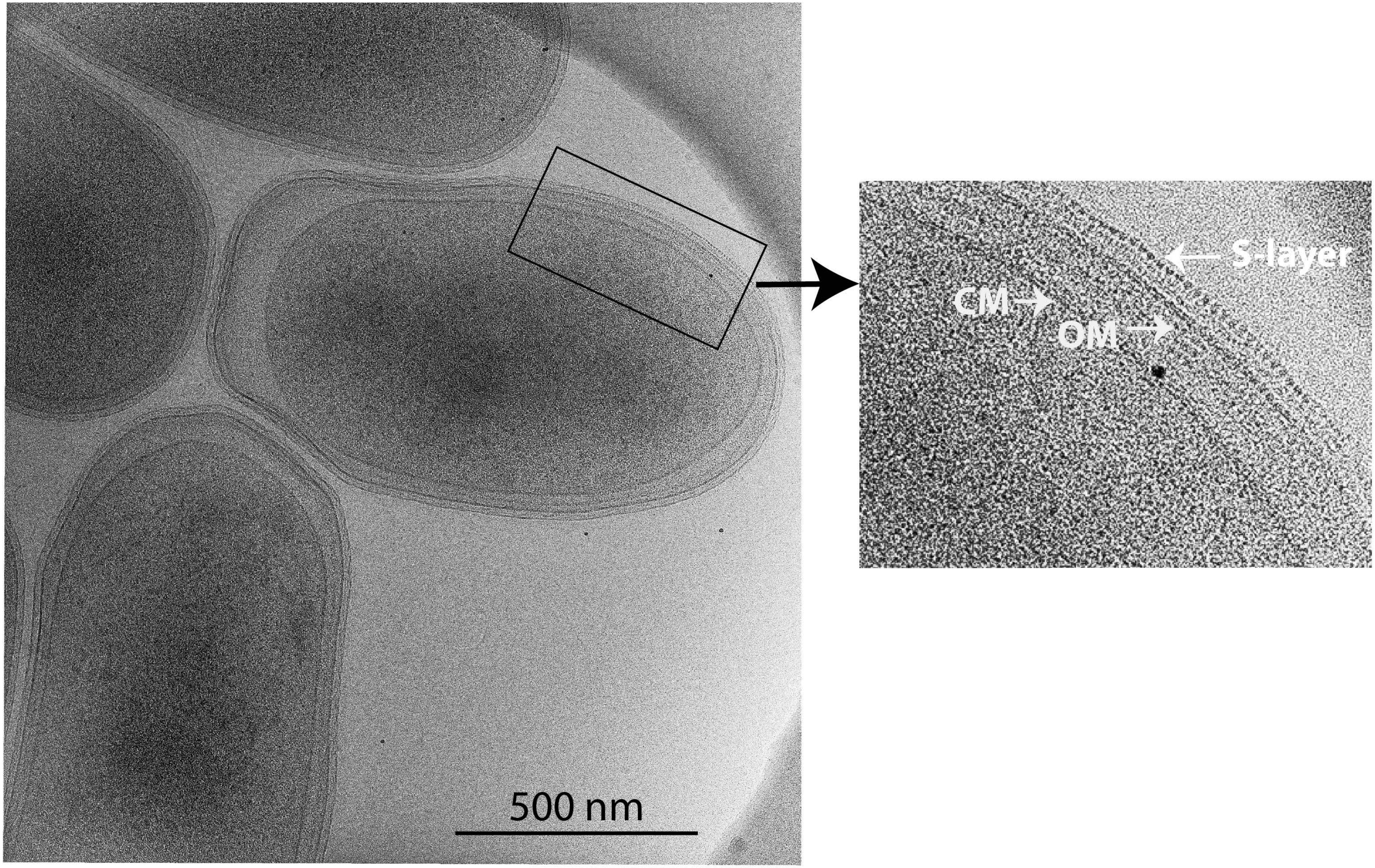

Figure S6K. ***Ottowia thiooxydans***

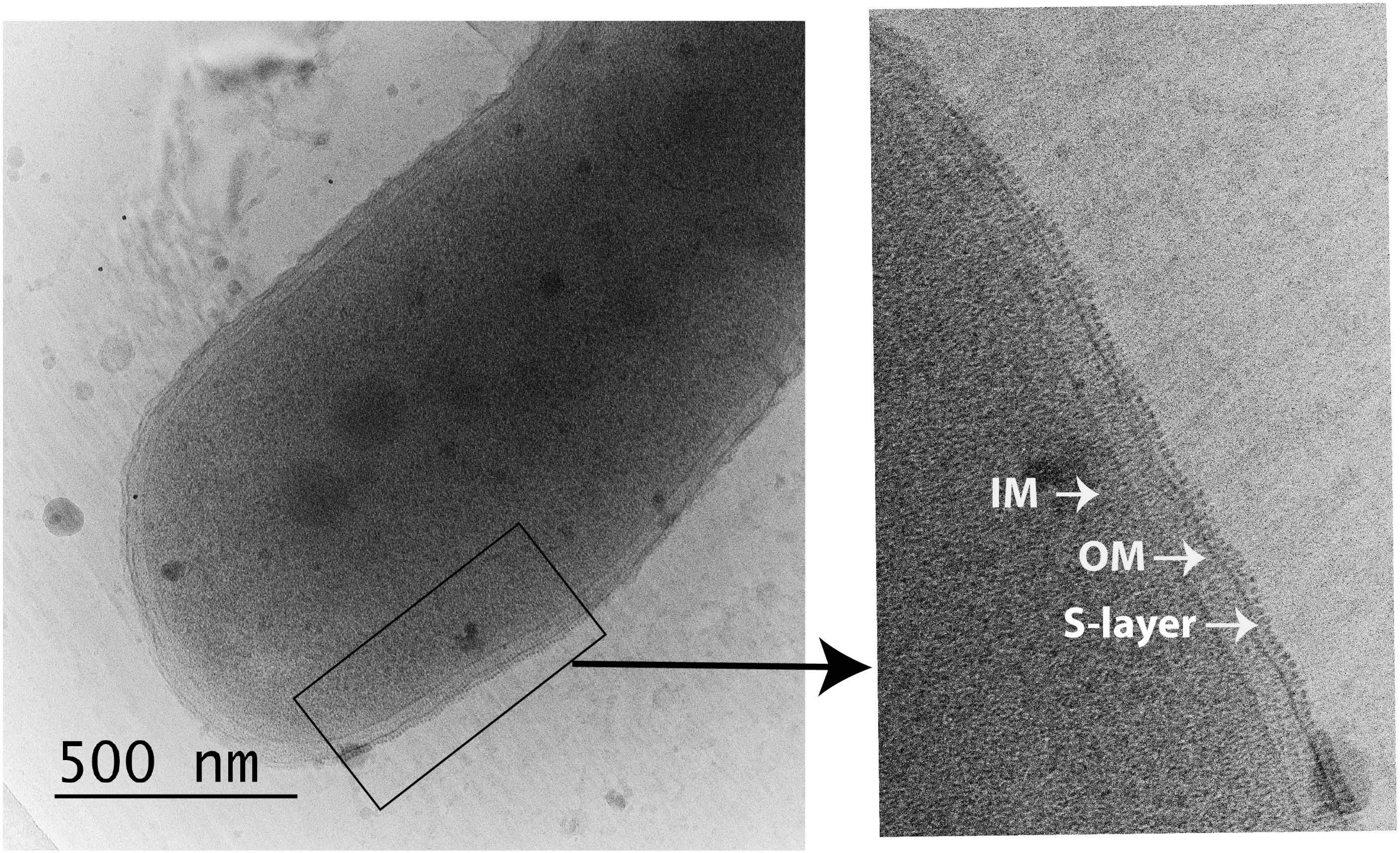

Figure S6L. *Simplicispira psychrophila*

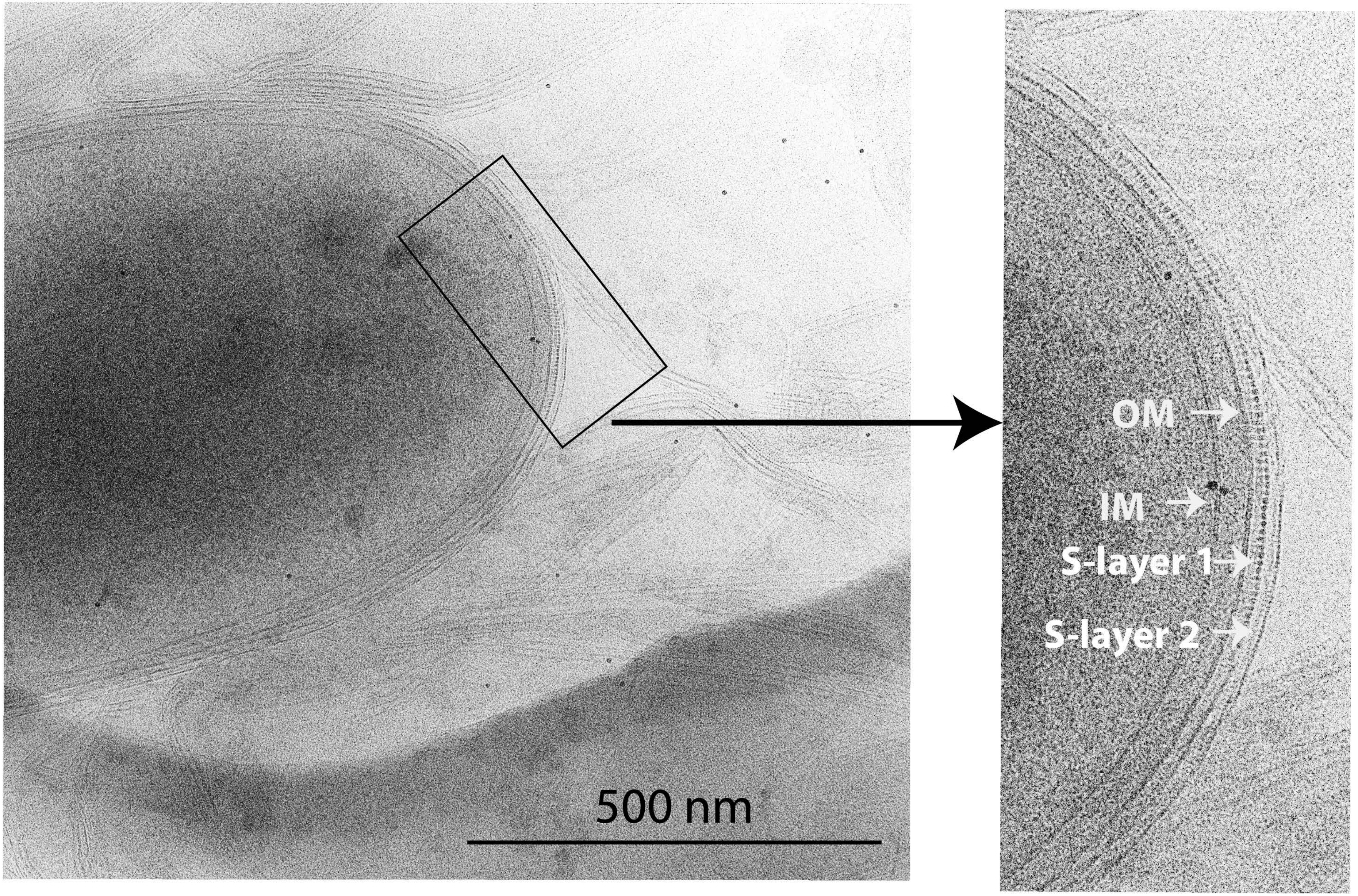

Figure S6M. ***Verminephrobacter eiseniae***

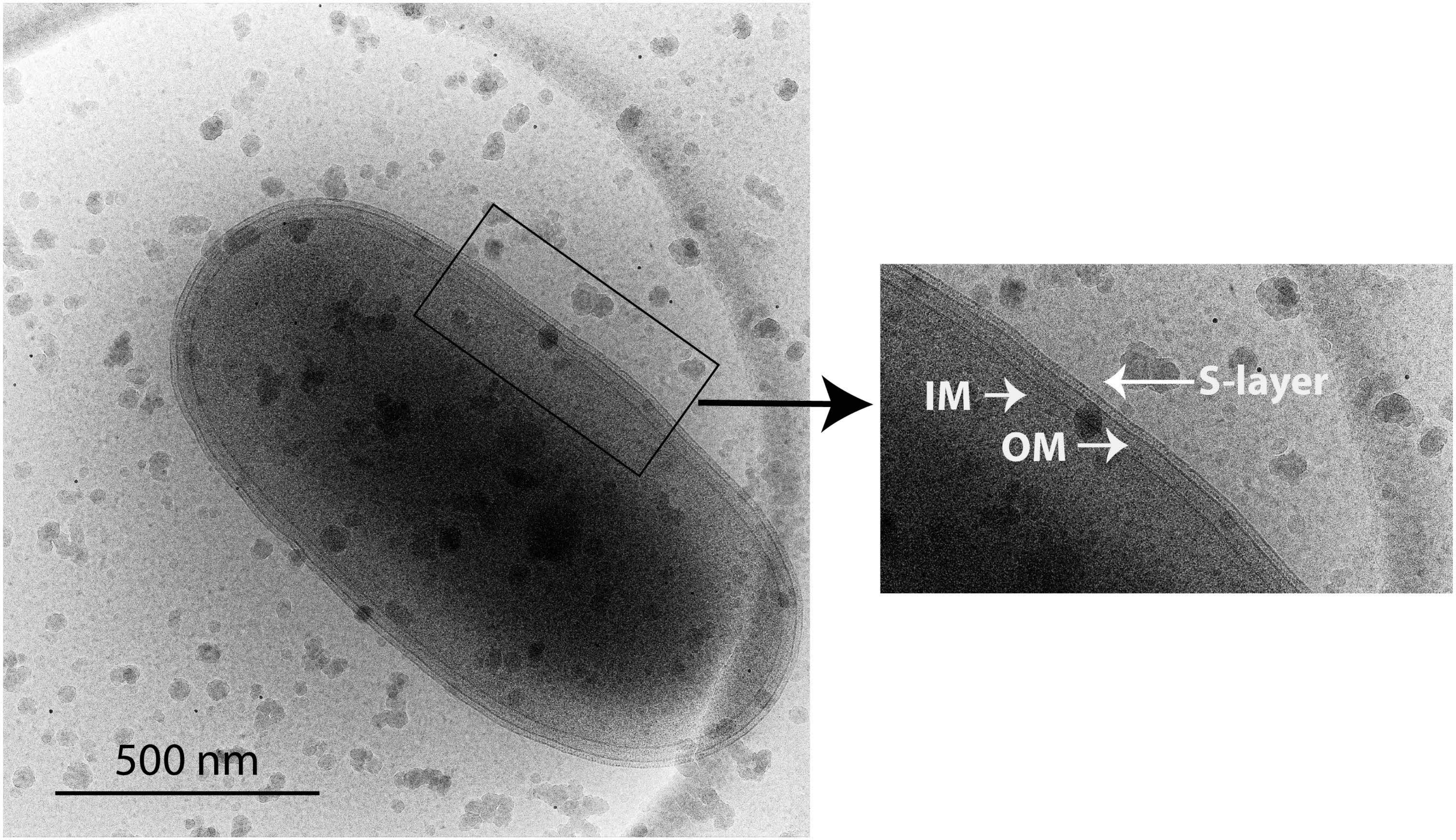

Figure S6N. *Congregibacter litoralis*

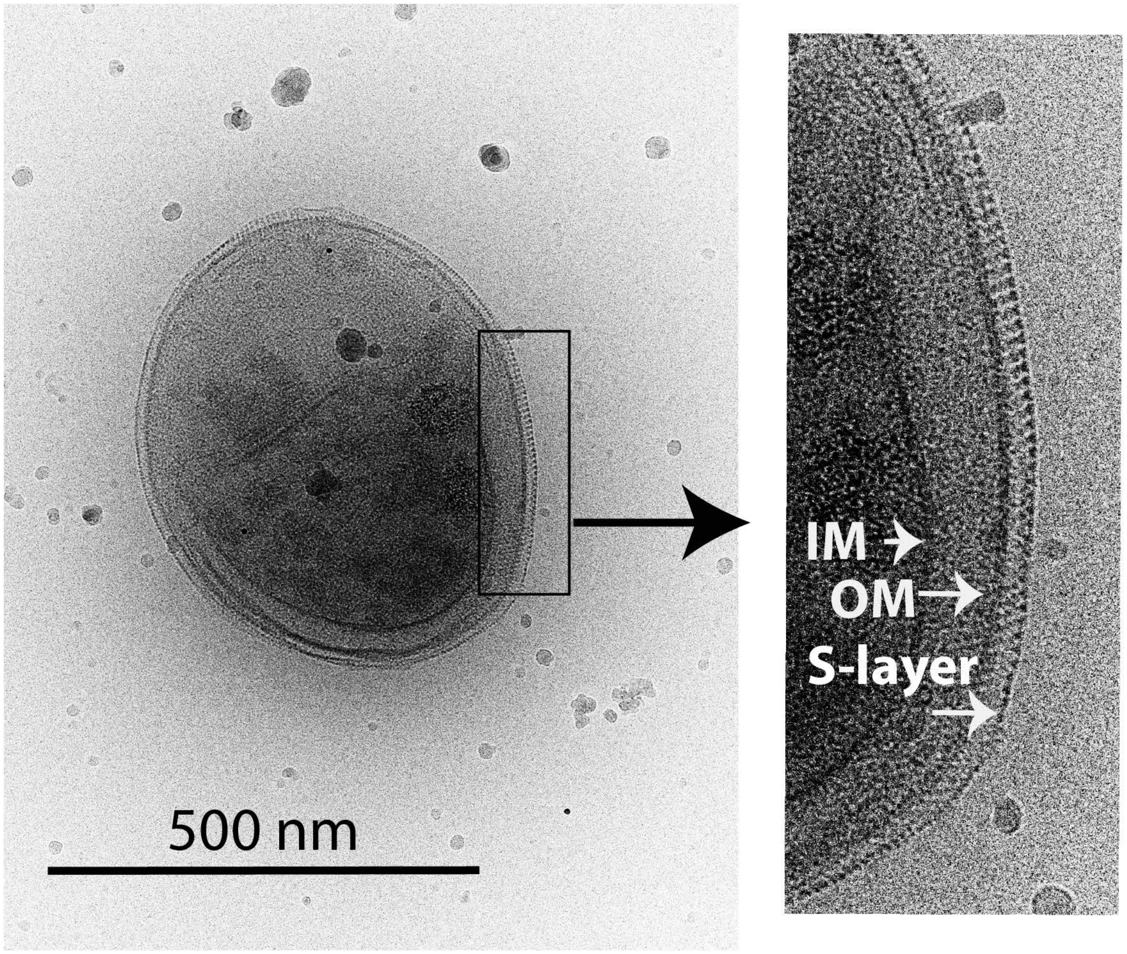

Figure S6O. *Haliea salexigens*

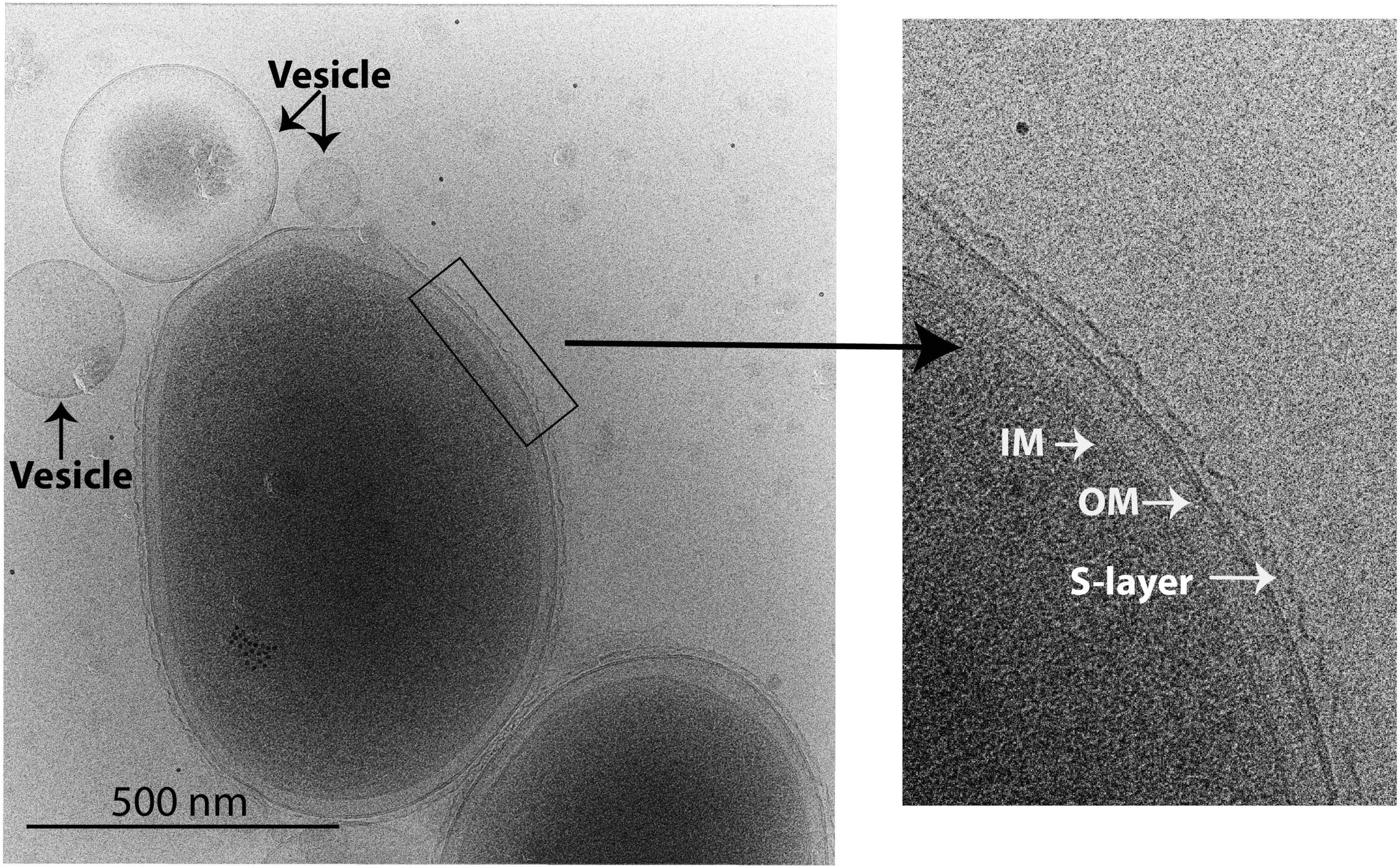

Figure S6P. *Rudaea cellulosilytica*

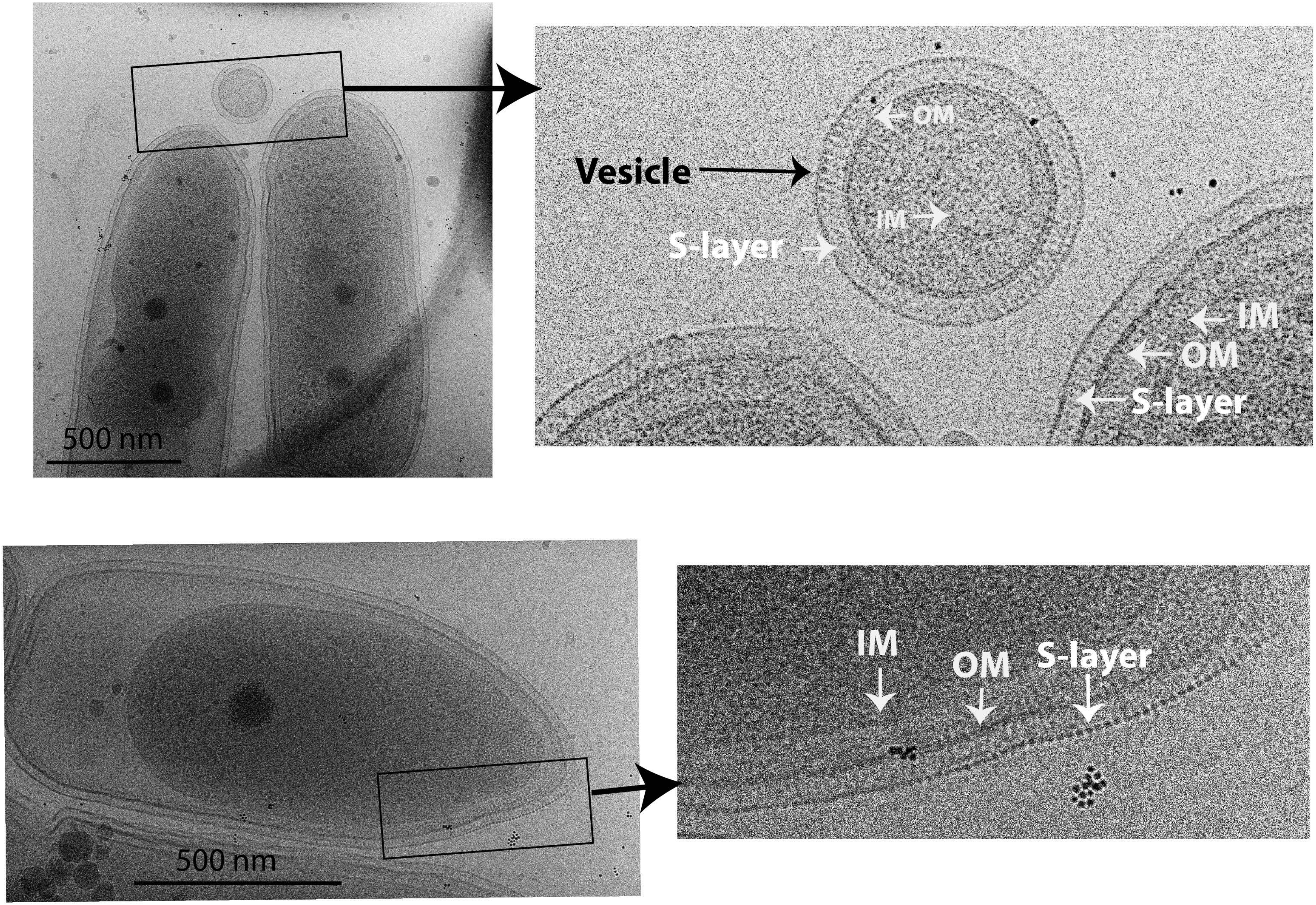

Supplementary Data File S1. Protein sequences of NpdA orthologs used in the phylogenetic analysis.

>WP_011797163.1 hypothetical protein [Paracidovorax citrulli]
MKKNVLALSIAAMIGGLGFAGAASADVVVGATPLTATNATSLSFAEGGVGHALLVPYFNAQNGNMTVLH
VVNTDTSRGKAVKVRFRGAQNSDDILDFQVFMSPGDVWTAAVTAGSDGVAQLQTADGTCTLPALAKNV
PQRFVTDRLNQGLATADLANQTREGYVEIFNMADIPATLAGSNTTNPLYTAIKHVNGVAPCTSSALNATLQ
NFTTPAAVAAAGFDTPTTGLVGDWYIINVAQTTTFAGAATAIRAENGGAPAVGNFVHFPQMASNAATPD
NFTADPLFRATNVFNAAGVAVTSPKIAAANYDLPDMSTPYTANGGVAVSPLVQATNLTNALAVTSITNQY
ATDASISAKTDWLFSMPTRRYNVAANYAAANQSPADTSNVRLFTDLNGSGGVADERFNPTNTSLQAVG
GAICVNSTGQAFFDREEQTQTAGAVFSPGSVTQTRFCGETSVLSFASGSVLGASVASQQLTTGAYTNGWS
RVDVPNNGLGLPILGASFIKLANPLASAGTSGTYGITWPHRFTRPAAQ
>WP_092832654.1 cell surface protein [Paracidovorax cattleyae]
MKKNVLALSIAAMIGGLGFAGAASADVVVGAAPLTATTATTLSFAEGGVGHALLVPYFNAQNGNMTVLH
VVNTDTSRGKAVKVRFRGALNSDDILDFQVFMSPGDVWTAAVTAGSDGVAQLQTADGTCTLPALTKNVP
QRFVTDRLNQGIVSTDQANQTREGYVEIFNMADIPPTLAGTTTTNPLYTAIKHVNGVAPCTPAALNATLQ
NFTTPAAVAAAGFDTPSTGLVGDWYIINVAQTTTFAGGATAIRAENNGVPAVGNFVHFPQMASNANTPD
AYTADPLFRTLNVYNSANAVPSNLPKIAAANYDLPDMSTPYTANAGVAVTPLVQATNLTNALAVTSITNQY
ATDGSISAKTDWLFSMPTRRYNVAANYAAANQSPADTSNIRLFTDLNASGGVADERFNPSNTSLQAVGG
AICVNSTGQAFYDREENTQTAGAVFSPGSVTQTRFCGETSVLSFSSGSVLGASVAAQQLTTGAYTNGWSR
VDVPNNGRGLPIIGASFIKLTNPLASAGTSGTYGITWPHRFTFPVAQ
>WP_053843492.1 hypothetical protein [Paracidovorax avenae]
MKKNVLALSIAAMIGGLGFAGAASADVVVGAAPLTATTATTLSFSEGGVGHALLVPYFNAQNGNMTVLH
VVNTDTSRGKAVKVRFRGALNSDDILDFQVFMSPGDVWTAAVTAGSDGVAQLQTADGTCTLPALTKNVP
QRFVTDRLNQGLVTADMANQTREGYVEIFNMADIPPTLAGSSTTNPLYTAIKHVNGVAPCTSSALNGTLQ
NFTTPAAVAAAGFDTPSSGLVGDWYIINVAQTTTFAGAATAIRAENNGLPAVGNFVHFPQMASNANTPD
AYTADPLFRTLNVYNAANAVPANLPKIAAANYDLPDMSTPYTANAGVAVTPLVQATNLTNALAVTSITNQY
ATDSSISAKTDWLFSMPTRRYNVAANYAAANQSPADTSNIRLFTDLNASGGVADERFNPSNTSLQATGGA
ICVNSTGQAFFDREENTQTAGAVFSPGSVAQTRFCGETSVLSFASGSVLGASVAAQQLTTGAYTNGWSRV
DVSNSGRGLPIIGASFIKLANPLASAGTSGTYGITWPHRFTLPAAQ
>WP_026433145.1 hypothetical protein [Paracidovorax oryzae]
MKKNVLALSIAAMIGGLGFAGAASADVVVGAAPLTATTATTLSFSEGGVGHALLVPYFNAQNGNMTVLH
VVNTDTSRGKAVKVRFRGALNSDDILDFQVFMSPGDVWTAAVTAGADGVAQLQTADGTCTLPALAKNV
PQSFVKDRLNQGIVSTDQANQTREGYVEIFNMADIPATIPGTATTNPLYTAIKHVNGVAPCTQATLAATLN
NFTTPQQVATAGFDTPSTGLVGDWYIINVAQTTTFAGAATAIRAEDNNGLPAIGNFVHFPQMASTANTPD
AYTADPLFRSTNVYNAAGVAVTAPKIAAANYDLPDMSTPYTANAGVAVSPLVQATNLTNALAVTSISNQYA
TDASISAKTDWLFSMPTRRYNVAANYAAANQSPADTANIRLFTDLNNSGGVADERFNPSNTSLQATGGAI
CVNSTGQAFFDREEQTQTAGAVFSPGSVTQTRFCGETSVLSFATGSVLGASVAAQQLTTGAYTNGWSRVD
VSNNGRGLPILGASFIKLANPLASAGTSGTYGITWPHRFTRPSVQ
>WP_092949656.1 cell surface protein [Paracidovorax konjaci]
MKKNVLALSIAAMIGGLGFAGAASADVVVGAAPLTATTATQLSFAEGGVGHALLVPYFNAQNGNMTVLH
VVNTDTSRGKAVKVRFRGALNSDDILDFQVFMSPGDVWTAAVTAGSDGVAQIVTADGTCTLPKLSKGVN
QPFVLDRLNPGIATADKANNTREGYVEIFNMADIPPTLAGSATVNPLYTAIKHVNGVAPCTQTALDATLNN
FTTPAAVAAAGFDTPSTGLVGDWYIINVAQTTTFSGGATAIRAETAGGVPAVGNFVHFPQLSTNAPTPDN
FTADPLFRTLNVYNASNVAATGLPKVAAANYDLPDMSTPYITAAGVAVSPLVQATRLTDALAVTSISNQYA
NDASISAKTDWVFSMPTRRYNVVANYAAANSSPVDTANIRLFTDLNVDGTVATERFNPSNTSLLASGGAI

CVSSTGQAFYDREEQTQSSGAVFSPGSVTQTRFCGETSVLSFADAGNSVLSASVARQNLTTGSFTNGWSR
VAVPNNNLGLPILGSSFIKLTNPQASAGTSGTYGITWPHRFTRPSVQ
>WP_111877457.1 cell surface protein [Paracidovorax anthurii]
MKKNVLALSIAAMIGGLGFAGAASADVVRGTGVGGDGGTITNGVWIPDAGTAPANWRLTATGATGLQL
SQGGNGHSLVVPYFNAQNGNMTVLHVVNTDTVHGKLAKVRFRSAANSDDILDFQVFLSPGDVWTAAV
TADANGIARLTTGDGTCTLPTITRNVAQTFNTGRLSTLAGDIANQTREGYVEIFNMANIDSNAIYGANRNA
NSALYTAIKHVSGTAPCTATALSATLANVTTEAQARQLGLDTPTAGLLGDWYIINVAQSTTFSGAATAVTAT
GGTGAGAGRGNFVHFPQSELQAPTPETFTADPLFRVAYTRTGANAEVTTAALVAANYDLPDLSTPYTLNA
GGTAVVDTRVQAYNLTRALAVTSITNQYANDPTITAKTDWVFSMPTRRYSVAANYNAGATTAANYRLFTV
LDTGSATNTLATNYAAWFAPENTALQGGLICVNSTGQSFFDREETTTTSGAVFSPTTVSQTRFCGETSVLSF
ADANTSVLGASVARQNVASGSFVNGWSVVRTTNSFQTTLGPVYGATAGTANAVQYNSQAGLPILGASFI
KLTNPQASAGTVGTYGITWPHRFTAAQ
>WP_092745325.1 cell surface protein [Paracidovorax valerianellae]
MRKNVLALSIAAMIGGLGFAGAASAGVVPGTGTFNGTNQLTTTNATVLQISDAGVGHNLLVPYFTAQNN
NMSVFHVVNTDTSNGKAVKVRFRSASNSDDILDFQVFMSPGDVWTAAVTQGSDGVAQIVTADNTCTV
PKLTANVPQKFILDRLNPSLAAGDKANQTREGYVEIFNMADIPPNNGGTAAAPTLYGAIKHASNVAPCSV
DGSVAQTLLNTVAVTNFTTEAQAATAGFNTPSSGLMGDWYILNLPQTTTFSGAATSVAARVAVNGADGR
GNFVHFPQTNASAGNVDTFTADPLFRTDARPATQGTTGGTYGAPLGTATLAASFFDLPDMSTPYTAAVPA
SATAAPLEQADLLTTALAVTSVTNQYATDTSISAKTDWVFSMPTRRYSVVANYAATGVVGNQNVVGITAP
NYRLFSQLNGGTVAGGEWFLPTNTSVDSVGNICVAADSQRFWNREETTAGAAGTPPVFSPGNPLAPSV
VRFCGETSVLAFADAGASSLGGNVARTTITAGTYTNGWGAINTTNGGNGLPILGSSFIKLSNPNVGNGVS
GNFGITWPHRNTRIQTVN
>WP_044395736.1 hypothetical protein [Acidovorax temperans]
MKKNVLALSIAAMIGGLGFAGVASAVVIPGGGAANSVDPVVDYADATKNKMALTNATALSVTTGGTGH
NLIVPYFTVQDGNMTVIHLTNTDTVNGKAVKVRFRGAANSDDLLDFQVLMSPGDVWTAAVTAAADGTA
QLSTADGTCTVPSLKGVTQKFDTRRLPTSVGAAGTREGYVEIFNMADISGKDLYTVGTTTSTKSALYTAIKH
VNGVAPCTATVIEPIMLKKDHTEETAVKAGFNTPTTGLMGDWYIINVAKTTTFSGAATAVTAVVSGTDSTA
AKGNFVVFPQLADAVGATIDNFTADPLLRTANIGTTKTAAGVASVAPTTVPAIEAAFYDLPDLSTPYVVAGG
TATAPITQAEILTGALAVKTITNQYATDAGISAKTDWVFSMPTRRYSVALDYRQTTPSRVYTNGIVGDTDPA
TAGVQAGAYFHASNTSLDSGKICVTSDKQAFYDREETTKTAGAVFSPGAVDKARFCGETSILSFGTSTSGVL
GAALAAQFTETAAYTNGWGVIDVTNGNVGLPILGSAFIKLTNPQASAGVSGNYGITWPHRFTK
>WP_252660345.1 cell surface protein [Acidovorax kalamii]
MKKNVMALSIAAMIGGLGFAGAASADVVVGTDAAQTAATYAGKIIAKPTATALAVTAGGVGHALVVPYF
NAQNGNMTVLHITNTDQTNGKVAKVRFRSASNSDDLLDFQVFLSPGDVWTAAVTADANGLAQITTADN
TCTVPALTTQSFNNSTSRLPGYLTEAQRTALTREGYVEVFNVADITALKSFDTNGALNAAGPNRSPLYTATK
HVSGVAPCSVAGSTARSTLNAAAIADYTTEAQVAAAGLDSPTGGLMGDWYIINVPQTTVFSGAATAIAAN
TGAGANGVANFRHFAQLGNALVTLNANGADGVTADPLFRALNVFDATGAAVATATLPMLNYDAPDLSTP
FFGAASSANTLAQATSLLSALAVKSVTNQYALDASITAKTDWVFSMPARRYNVVANYAAVTGTSGATGPTS
SNAKYRLFTDLNASGTQAGEWFYPAVSGAYTTGGGTYKTGGNTTVDTNGNICVYADGQKFFDREETSAT
AGPVFSPGTASSVAFCGEVSVLAFSDTGNSVLGASVARSTITSGIYTNGWGVIDTANAAQGLPLLGNSFIKL
TNPQAGVGVSGTYGISWPHRYTK
>WP_226447122.1 cell surface protein [Acidovorax radicis]
MKKNVLALSIAAMVGGFAGVAQADVFAPGAAATGAVLGTNVAAEPGVAGAVAVNATVLEVNSNNVGH
MLVVPYYTAQNGNMSVIHLSNTDRANGKAVKIRFRGAANSDDVKDFQLFLSPGDVWTGAVLQGADGA
AVLYSADKSCTVPAIPATGTAFATARLNATTNVNGTREGYVEIFNMADIPSVKLYTSTQTSSGTVAGAAGTA
NSVLYTAIKHASGVAPCSVAGSASRTLLDEIAMTNYTAAAHAASVGFGAATTGLSADWYILNLAQTTAFSG
SATAIEARVAAAGAAGAANYVHFPQTDAGSGAFFATANKSADTYTADPLLRTLVNDFAKASVTGPVVTPLF

IDLPDMSTPYLAAQAPAGAAGDANLPKLQAVALTAALSRTSVSNQYALDTGISAQTDWVFSMPTRRYSVA
ANYKAATTSAANYRLYSDLSVIATGPALPVVAAATHGLADEWFYPGNTQVDSRGNICVNADAMRFFDRE
ETTTGNAPIFSPAVASQLALCGETSVLSFSSGKVLGSSSDLTATTMASPYVNGWTLVSTANAFVGANNTNA
FTGGLPILGDAFIKMKNDSAAQGFASTFGLTWGHRYGR
>WP_005793489.1 hypothetical protein [Acidovorax delafieldii]
MKKNVLALSIAAMIGGLGFAGAASAQVVSPLTVNESGTGHMLLVPYYTAQNGNMSVFHVVNTDTANGK
ALKVRFRGASNSDDVLDFQVFMSPGDVWTAAVTQGSDGVANLVTADGTCTLPAIAKGVAVPFVQDRLPS
AMTAADKANNTREGYVEIFNMADITSAKDPLFGGVGNLVPVNAPLYTAIKHVNGVAPCTDSVLQSTLTTE
AATAGLVAPTDGLTGSWYIINVPQTTTFAGAANAIVATGTTNNVFSAQSTGTAGLFTADPLFKSGVLTAQKY
DVPDLSTPYVGAASQAAADTQASQLTAALAHAQVINQYANDASITAKTDWVFSMPTRRYSIAANYKAPIG
AGVLATSAYAAASATEKAAAYRLINDGVTGSYFVTASNTTVNSIGQICASSIGATFYDREETSKTSGAVFSPG
TVSQTPLCGEVSVLSFADAGASVLGASVARSNVTGAYTNGWGRISFSAGVPVLGASFIKLTNPAASAGVSG
NYGITWPHAYK
>WP_074930106.1 hypothetical protein [Paenacidovorax caeni]
MRKNLLALSIAAMVGGLSGAANAQVALFDNSLATNTTGAGNTPAKAYVGTTQAGFDPAAVLAPTTTGV
GHVLVVPYFTTQTSNHSLLSITNTDLVNAKAVKLRYRGASNSDDVFDITVYLSPGDVWAADVAAENDFSR
LTTDDNSCTLPSKADIKATNNGRFKVNRVRGSDAAQTREGYIEILNSADIRPFLNNDAGVQDPTKPNPLYA
AIKHNADGVAPCTAAVMDQQAIALDFTDPDSPNTPRNRGYSWPTGGLFANWLIVNVATNASFSGEAVA
LRASSSTTLDTTGSGNLVWFPQTTETVSQANAGQLTADPLLSIGTPAPLRAAMYDFPDLSTPYTAAAAGAP
STQANNLAFALETQSVTNEYLTAASSGGFATDWVFSMPTRRYAVAMNYAAASAPAGVSRNALVARHFGA
GNLTTNANWQICVDAGNLRGFDREERSKTTFVISPDMSMRFCGEASVLSFNSATSAVLGAQIATQQIPTG
FAEGWFTVRTPGVTSTAANPVGLPVIGYAAAKANSGNLGGTWMHRTAR
>WP_182343689.1 cell surface protein [Comamonas koreensis]
MKKNVLALSIAAMIGGFAGAASAQTFTAPTATKFEQSEGGVGHILVVPYFTTQNDNMSVFHVVNTDTTN
GKALKVRFRGAANSDDILDFTVFLSPYDVWTGSVQADANGSYFMTADNSCTYPNIHGQKVSFVQDRLA
NGWTAEKRVNNTKEGYVEILNMADVKPGSDLFKTIKHAANGAPACADSVLERTLAINPTTESAAATLGYD
TPTGQLTGDWYILNVAQTTTFSGAATAIKAVDDAGANARGKFVAFPQDNQSTGLNNDLYTADPLFKTTG
AVFADNSPAVNLVAAVNYDFPDLSTPYLTSTATPEEQASLLTQAVANTGVSNQFATDTLIQAKTDWVLSMP
TRRYSVGANYAATDAKQYRVFNPLVVTAGGARFFDAGSTTVDTDGNICVTTTSNTFYDREESSVRNGAVIS
PGTAKIISLCGEVNVLAFKDTGTSVLGATVARQTAAVTYENGWGQVNYTRPLPVVGSSFIKLTNSGGAAN
GFSGTYGITWPHRFSK
>WP_184705622.1 cell surface protein [Comamonas odontotermitis]
MKKNVLALSIAAMIGGFAGSANALTAATATAFEQNDGGAGHILVVPYFTTQNNNMSVIHVVNTDTQNG
KALKVRFRGAANSDDILDFTVFLSPYDVWTGSVQADANGSYFMTADNSCMYPDIRKVKQSFIQDRLSDS
WSAEKRVNNTKEGYIEILNMADIPPSTVSGTLYPAIKHKSGGAPSCDEAVLAKTLSINPEKAADATALGYDT
PTGGLTGDWYILNVNQTTTFSGAATAIKAVDANGANARGNFVAFPQDNLSPAVGNTDLYTADPLFISTAV
DKGNVAQNKLIQIANYDLPDLSTPYLTNNVGVPAQQASTLTAAVANNGVANQFATDTLIDAKTDWVLSM
PLRRYSVGANYAAATKDNAYQVFNPAVLNPANGQPFFARSITSVDADGNICVDTASNTFYDREESSVTNG
AVVSPGTAKKIRLCGEVNVLAFKDAGASVLGATVARQTAAVQYENGWGKVNYGSNNAFPVVGSSFIKLTN
SGGAANGFSGTYGITWPHRFSK
>WP_182325627.1 cell surface protein [Comamonas piscis]
MKKNVLALSIAAAMIGGFAGAASAQTFPNSTMVQPDATTLVRAEGGAGHILVVPYFTSQNDNMSVFHV
VNTDTVNGKALKVRFRGAANSDDILDFTVFLSPGDVWTGSVQGNENGSYFLTNDKSCTLPDIRGQQVQF
VTDRLPSGATAAQTKEGYIEILNMADITGTATDMYKATKHVAGVAPCTSTVLDATLNINPTDVDSVTAAKT
MGFETPSGKLTADWYILNVNQTTTFSGAATAIAATGGNGRGNFVLFPQDATNSAATAANVADLTADPLLQ
QGMVKAVNFDFPDLSTPYLATTGTPEDQATDLTTSLSNSGVSNQYALDTGINAKTDWVLSMPTRRYSVG
ADYTKKPTDAGYRVFNDGVGGFFTEANTYVNANNGLICATTNSYTFWDREEQSKKNGAVISPSTLKGLDF

CGEVNVLSFNDAAGTSVLGASVARTNVNVDYQNGWGAISYTNSLPVVGSSFIKLSNSGGLANGFSGTYGI
TWPHRYAAPAVAP
>WP_029695889.1 hypothetical protein [Comamonas badia]
MKKSVLALGVAAALGGFAGSALAITDAAPVPGTATALTLSPTGTGHILIVPYYSAQNGNKTLLSLTNTDNTN
GKIVKIRFRGAANSDDVNDFQVFLSPGDVWTADIHQGPNGVAALTTADKSCTKPADVNGDFGTGRFKST
WDADRLASNTREGYIEILNMADVPPAAALYNAIKHKDGVAACASSSIANGTAGSAAAWTYLDGNPKQGT
GRLTADLTANTGITTPTTGLMANWTIINVPRAVAWAGAATAVEATTGPGTAPTDGAIVYFPQVNQATGG
DLAGIAGAYTADPLLTGALVVGDAQFDLPDLSTPYAGAANPSLQAQELSTAISTSSVSNEFWTLQGIFGATD
WVFSMPTRRYALAVDYTPATSVRVYNAANATQFAAANTAMNGDQACVTGVTWNYWNREEATQAGDV
TNTTRSPGGGAPAGAMFCGETSVLSINDVAAGTAPTKALGAEVAVTNFEQKSASGSLFEEGWMKITTPG
AVATNLPILGSAFMLAVNPDVDSGTSGNFNMTFPHRTER
>WP_031255557.1 hypothetical protein [Curvibacter lanceolatus]
MKKRILSLSIAAMIGGVGLAGGASAAVFTPATAGSTLSLSSTGAGHILLVPYFSTQSGNATLLNIVNTDTTNG
KAVKVRFRGAANSDDLYDFQVLLSPGDVWTASVSQDATTGISTLATSDATCTLPTSVNGAFQTGRVQQLS
TAAAIANQTREGYIEILNMADIPKGGLVDQATLANGVYTIPTTNPLYTAIKHVNGVAPCSTNAAGAAAINLL
VQDPVAYSSQVTTLTSATATASNTAQALGLTYPTGKLFADWTIINVANTTTYSGQATAVSADSANLVFFPQIS
TSITNTPVSSTAGSTTGVVSALLDSVTADPVFRKTAVAYNQATVLGTSNPNGTSAANVANGVVVNAASSTA
ASIANPVVAAAYYDLPDLSTPYVGLVNTSTVAALNQSNALTGTLARGTVTNEYLTSSSISGATDWVFSMPT
RRYHTAYAYAYAGATGNGQVYTDYTSSAAATAYTYFGPLNTTVSSTNGLQICTKLATGVTSFGREEQAPATS
NPGFVVSPGTVAATSTLSLCGETSVLSFGVASAVLSATVANTVYPAASLPAADGWATFSTAANYTGTLPIIGY
SAEKAVNGAVKAGVSGNFGLLFSHR
>WP_027476560.1 hypothetical protein [Curvibacter gracilis]
MKKRILSLSIAAMIGGVGLAGGASAAVFSQGAGTLTNVGNAAGNILLVPYFSTQGGNATLLNITNTDTVN
GKAVKVRFRGAANSDDLYDFQVLLSPGDVWTASVSQDATTGISTLSTTDATCTLPTSVNGTFQTGRVQQF
STASSIANQTREGYIEILNMADIPKFGLFDQGTVSGTTITAPTTNPLYTAIKHVNGVAPCSTNAAGAAAINLL
VQDPTTYSATVAFTTGAAAAGTIAVNTKTASLAATSSSSAQGLGLVFPTGKLLADWTIINVASTTVLSGQAT
AIAADTANLVFFPQVGTAIPTTNSTAVSTVAIATGTAATLATPTLDAVTADPIFRTTNLAYNQGTVLANANV
NNGTAANNANGTIQNAASSTAASLASPAVAAAYYDQPDLSTPYVGVVLGGATATANVSALNQSLTVTALL
AKSSVTNSYLTTSALNAATDWVFSMPTRRYHTAYAYSYAGATGNGQIYTDYTKAATVTGAGATGSAYRFFS
PSNTSVSTTNGLQICTKLATGVTAFGREEQVAAVSNPGFVVSPGTVAASATLSLCGETSVLSFGKASALLSAT
VANTVYPAASLPAVDGWATFGTSSGAANVVGTLPILGFAAQKAVNSAVSAGVSGNFGLSYTHR
>MBP7606356.1 cell surface protein [Giesbergeria sp.]
VVGAAPLTTTNAQQLQFAEGGIGHALLFPYYNAQNGNMTVLNVVNTDTSNGKAVKVRFRGALNSDDVL
DFQVYMSPGDVWTAAVSAGSDGVAQIITNDGSCTLPKLNKGVGQSFVQDRLNGNLSVADKANNTREGY
VEIFNMADVPPTLRGANTTNPLFTAIKHVNGVAPCTDAALNGTLVSLTSPAAVAAAGFDTPSTGLVGDYYII
NVAQTTTFSGGATAIRALTNTTGAGVNARGNFVHFPQSASTANNVDNYTADPLFRSANVTDAAGVAVTS
PKIAAANYDLPDMSTPYFTAPGANAPLVQARELTDALAVFSVVNQYANDGSINAKTDWVFSMPTRRYNV
AANYAATTTTAADYRLFTNLPVNRFTAGNTAVVNGAICVNSDGTTFYDREETSKVDGAVFSPGTAVKTRFC
GEASVLSFADSGVSVLGGSVARQNVSGVYVNGWANVNTSNGGLGLPILGSAFIKLSNPQATSGMSGTYG
ISWPHRFNRFQ
>WP_275825027.1 cell surface protein [Delftia tsuruhatensis]
MKKNVLALSIAAMVGGLGFAGVASAAVIGGSNAGTDAKAAAAASQLLQATNGGLTLAHGGVGHALVVP
YFNAQNGNATVLHLTNTDTVNGKAVKIRFRSAANSDDLLDFQIYLSPGDVWTGAVSANGTDGATLVSAD
NTCTVPALTKNVAVPFGTRRLNPSLSDADKANQTREGYVEIFNMADITSAAAWNADGVAPVDLSGNRRS
PLYTAVKHVNAVAPCSVAGSAARSLVDTVAVTNFTEATAAQAGLQTPTSGLMADWYILNVAQTTTFSGAA
TAIQATGRGNFVHFPQVNQEAAGVSANATTADPLFRTANVYGQDGKNIATAALTMNYSDLPDMSTPYTG
GVTDPRVQASNLTTALAATSVTNQYATDAIISAKTDWVFSMPTRRYNVVANYAAAKTSDANYRLFTNLNT

AAAEQTNNWFYPAAANNIAAGAGAYKGNGNTTVDSKGNICVLADGQKFWDREETTSGVIPEFSPGGTT
SVQFCGEVSVLAFKDTGNSVLGASVARTTATSGTYENGWGVLTTTNSGLGLPLIGSAFIKLENPSARQGMS
GTYGVTWPHRYTRVAP
>WP_203399988.1 cell surface protein [Delftia lacustris]
MKKNVLALSIAAMVGGLGFAGVASAAVIGGSNAGTDAKAAAAASQLLQATNGGLTLAHGGVGHALVVP
YFNAQNGNATVLHLTNTDTVNGKAVKIRFRSAANSDDLLDFQIYLSPGDVWTGAVSANGTDGATLVSAD
NTCTVPALTKNVAVPFGTRRLNPSLSDADKANQTREGYVEIFNMADITSAAAWNADGVAPADGTGNRRS
PLYTAVKHVNAVAPCSVAGSAARSLVDTVAVTNFTEPTAAQAGLQTPTSGLMADWYILNVAQTTTFSGAA
TAIQATGRGNFVHFPQVNQEAAGVSANATTADPLFRTANVYGQDGKNIATAALTMNYSDLPDMSTPYTG
GVTDPRVQASNLTTALAATSVTNQYATDAIISAKTDWVFSMPTRRYNVVANYAAAKTSDANYRLFTNLNT
AAAAQTNNWFYPAAANDIAAGAGAYKGNGNTTVDSKGNICVLADGQKFWDREETTSGVIPEFSPGGTT
SVQFCGEVSVLAFKDTGNSVLGASVARTTATSGTYENGWGVLTTTNSGLGLPLIGSAFIKLENPSARQGMS
GTYGVTWPHRYTRVAP
>WP_180318125.1 cell surface protein [Delftia acidovorans]
MKKNVLALSIAAMVGGLGFAGVASAAVIGGSNAGTDAKAAAAASQLLQATNGGLTLAHGGVGHALVVP
YFNAQNGNATVLHLTNTDTVNGKAVKIRFRSAANSDDLLDFQIYLSPGDVWTGAVSANGTDGATLVSAD
NTCTVPALTKNVAVPFGTRRLNPSLSDADKANQTREGYVEIFNMADITSAAAWNADGVAPVDLSGNRRS
PLYTAVKHVNAVAPCSVAGSAARSLVDTVAVTNFTEPTAAQAGLQTPTSGLMADWYILNVAQTTTFSGAA
TAIQATGRGNFVHFPQVNQEAAGVSANATTADPLFRTANVYGQDGKNIATAALAMNYSDLPDMSTPYT
GGVTDPRVQASNLTTALAATSVTNQYATDAIISAKTDWVFSMPTRRYNVVANYAAAKTSDANYRLFTNLN
TAAANQTNNWFYPAAANDIAAGAGAYKGNGNTTVDSKGNICVLADGQKFWDREETTSGVIPEFSPGGT
TSVQFCGEVSVLAFKDTGNSVLGASVARTTATSGTYENGWGVLTTTNSGLGLPLIGSAFIKLENPSARQGV
SGTYGVTWPHRYTRVAP
>ROR50522.1 hypothetical protein EDC60_0275 [Diaphorobacter nitroreducens]
MKKNVLALSIAAMIGGLGFAGAASADVIVGTGVAAGAGSVNVQQGLLIDAVTPANSTMSGQALGANLA
LSEGGVGNMLLVPYYSAQNGNMTVLHVVNTDTLNGKAVKVRFRGAANSDDVMDFQVFMSPGDVWT
AAVTAGADGVAQIQSADNTCTVPALGNNTPQAFATNRLDPNKSDAEKANGTREGYVEIFNMADIPSDKL
YTVGTTANTNSALYTAIKHVNAVAPCSVAGSPAEATLFGVALTNYTAEADAANAGFNTPTGGLMGDWYII
NVPETTTFSGASTAVVADGRGNFVHFPQIDTGVAAAAIDAFTADPLLRTDALKTDGTAFGATPALAAGYYD
LPDMSTPYLAADIGNPKAQAVKLTSALAVTSVTNQYANDESITAKTDWVFSMPTRRYSVAANYAAISGTT
PANDSNTSYRLFSDLIVGVTPDAAATLQDEWFFAANTPVSTDGKGNICVKADAQKFWDREETTPGASTP
GPVFSPRTPGVAAVVSLCGEASVLAFKDLGNSVLASSVARSTITSGTYTNGWGVVTTANDIGAPGAGVVE
GLPLMGASFIKLSNPQVAPGLSGTYGITWPHRFTR
>WP_166067545.1 cell surface protein [Diaphorobacter sp. HDW4B]
MKKNVLALSIATAVVGFAGSAHAIVTADTTGTATALQFSNTNQGHMLLVPYFSTQGGNATLLSLINTDTVN
GKAVKVRFRGAKNSDDIFDFQVFLSPGDVWTANVSQNKDGLSYLTTADASCTKPEKSVINSTPFVTQRLN
PKSTDLAGETREGYIEIFNMADIPSSTSGVYPLIKHANSVAKCADNGTNTAWSTLDTDQINLAAYQALGLQ
QPTNGIAANWTIMNVPKALSWSGTAAGIEAVDATAKLAKGNLVYFPQMSEAASNVNGFTADPLFTNSIV
SASQYDLPDLSTPYAGGAVTPRVQADNLSKALAATQVSNEFWTLASIEAETDWVFTMPTRRYNVAVDYS
GTTPKATYATGATYFTASNATISGDAICVKGVTSFVWDREENTTVSGPVISPGSAAAPFCGEVSVLGFNNA
GAKATAVVGATVAIKDLDTGNFVNGWGGLRTPNGGLGLPVLGSAFVKAYNPAAAAGVAGNYGINWTHR
YTRP
>WP_187725498.1 cell surface protein [Diaphorobacter aerolatus]
MKKNVLALSIATAVVGFAGSAHAIGTIGGTATANDLQFGNTGTGHMLLVPYFSTQDGNANLLSLINTDTV
NGKAVKVRFRGARNSDDVFDFQVFLSPGDVWTANISKNKDGLSYLKTEDNTCTKPSKAKINDTPFITSRL
NPNVTAEEKANNTREGYVEIFNMADIPKSTTGLYPLIKHANSVAKCADDGTNLAWSAINKDLANEAAYKN
PAIGMTNPTTGLAANWTIMNVPKALSWSGAATAIEAVNSAVNPSALASGNIVYFPQVSEPVTNPGTFSS

DPLFVGSAASPTTPAYAAAMYDLPDMSTPYTVYTAESTNWPADQAIKLADAITTRAVINEFWTEPTIKAET
DWVFSMPTRRYAVAVDYSATKPTAVYNTTVNKHFTSANTVMNGDAVCVRNVTYTVWDREENSPASPD
DVVISPGTPTAPTLFCGETSVLSFNNAGAAQSAVLAASVSLKDMDTGYANGWAQLNTPGARVAGLPILG
QAFVSAYNPSVASGTAGNFGVNWGHRFVRVTP
>WP_194770285.1 cell surface protein [Diaphorobacter caeni]
MKKNVLALSIATAVVGFAGSAHAIQTIGGTTTPATANDLQFGNTGTGHMLLVPYFSTQDGNAMLLSLINT
DTVNGKAVKVRFRGARNSDDIFDFQVFLSPGDVWTANVSKNSDGLSYLTTEDATCTKPAKATLNSTPFITS
RLNSKVTAEERANGTREGYIEIFNMADIPKSTTGGLYPLIKHKDSVAKCANDGTNTAWTAINTDLADEAAY
EAPAIGMANPTTGLAANWTIMNVPKALSWSGTATAIEAVDSVAVPPVLGTGHIVYFPQTNVNVVNPTTY
TADALFGANAAGKVYVKPAMYDLPDMSTPYTQYTTAAATWPGDQAVKLADAITTRAVINEYWTEPDIKA
ETDWVFSMPTRRYAVAVDYAAASGPEAVYNPYVNKHFAASNTQMNGDAVCVLNVSYTAWDREENSPE
SPDDVVISPGTPTAPTLFCGETSVLSFNNAGASQSAVLAASVALKDMDTGFTNGWAQLNTPGATSAGLPI
LGQAFVSAYNPSVAAGTAGNFGVNWGHRFARP
>WP_187598085.1 cell surface protein [Diaphorobacter ruginosibacter]
MKKNVLALSIATAVVGFAGSAHAIQTLGTAATATQLAFGDTGIGHMLLVPYFTTQNGNATLLSLVNTDTVN
GKAVKVRFRGAKNSDDVFDFQVFLSPGDVWTANVSQNSAGKSFLTTEDNSCTRPKSVNREFITDRVSGK
VDKNGETLEGYVEIFNMADIVKDSLVYNVIKHKNGKAPCWDSAVPAELAAWNKLSNDLAAEADYALLGL
VNPTTGLMGNWTIMNVPKALSWSGSATAIQAVKADGTLGTGNVVYFPQTNNPVAAANVALFTADPVL
NTPAAAVAAAMYDLPDMSTPYLNGAATPSKQAEDLAKSIATTHVINEFWTDDGIMANTDWVFSMPTRR
YAVAMDYKNDKALQNTANVLHFDTNKNVVKNGDALCVNDPSATIWGREEETEVASNEDVISPGTPAEKK
AFCGEVSILTFGEEATSSKSAVLSAKLALSGVDSSKYQNGWADLATPGATGVGLPIIGGAFASAYNQAVDQ
GTSGNFSVTWGHRFIRP
>MBN9576710.1 cell surface protein [Alicycliphilus denitrificans]
MKKNVMALSIAAMIGGLGFAGVASADVIVGAGTGTGADAQTVTDIQKASPAGLMVKANATNLEVSRGG
VGHALVVPYFNVQNGNMTVLHVTNTDQKNGKVAKVRFRSAANSDDLLDFQVFLSPGDVWTAALTADA
NGLAQIVTADTTCTVPALASGVAQSFNNSTARLPAYLDETARNALTREGYVEIFNVADITNVKAWDASGGT
TGTTSFSPLYNAVKHANGTPPCATAGSAARTLLNNVAMADYLADAAGEDAAAKAGLQTSTGGLMGDWY
IINVPQTTVFAGPATAVVANTGAGTAPARANFMHFAQLGNPLATANADGANGVTADPLFRNANVFDATG
TAVATAAVPMLNYDAPDLSTPFFNAAGNNNARAQAQALLGALAVKSVTNQYALDPSITAKTDWVFSMPA
RRYNVVANYAAATSASNATSSNAKYRLFTDLNGAGQAGDYFYAAVSGAYATGGAAFKNGGNTTVEAGTG
NICVYADGQKFFDREETFSTSGPVFSPGTASSVAFCGETSVLAFQDTGKSVLGASVARSTVTSAIYTNGWG
VLDTTNAGLGLPIIGDAFIKLSNPNVSAGMSGTYGITWQHRFTK
>WP_145892328.1 cell surface protein [Caenimonas sp. HX-9-20]
MRKNALAMSIATLVGGMGFVGAASADVIVGTGAAPTSAVLGATTATQLVLAAGGIGHQLITPYFNAQNG
NATIISVTNTDTVNGKVMKVRFRGASNSDDILDFTVLMSPGDVWNATVTAPTTTAPAQIVTSDKTCTLPQ
LAAGVPQQFVTARLTSKSGNDIPNNTREGYIEIFNMADIPSTQSLYDGSAVAKQSNLYTAIKHVAGTPPCTS
TAINTAILDTNHTAEATAANRGLATPTTGLFGNWTIINVPQTTTYSGSMVAVRALTAGNVDGRGNFVVFP
QSATQYPGVINNVTADPVLRTANVCTSLTAANVCTAAAGLPAIPAAFFDLPDMSTPYAGAATPLIQAGTLTA
ALAVQTVVNEYATDAIISAKTDWVFSMPTRRYSVAMDYSPATSRRLYSQTLDPTLNLPFFHDSNTRINTGN
AQQICVDATAQTFYDREEQTKSSGAVFSPGNISITTFCGETSVLSFADSGVSVLGGTVARQDTSSSAFTNG
WGVVNVRSGATNLGLPVMGGSFIKATNPQVAAGQSGTYGVSSEHRFTR
>WP_275851194.1 cell surface protein [Extensimonas sp. H3M7-6]
MKKNVLALSIAAMIGGLGLAGGASADVIPGATKMTPSNASKFSVSEGGVGHILLVPYFTAQDGNMSVIHI
TNTDQTNGKVVKVRFRGAANSDDILDFTLLLSPGDVWTGAVTAGADGVAQLATADKSCTYPAIPSGGVSF
VTSRLTAANMDVASNTREGYIEILNTADIPGPSVTGTATALYTATKHVNGVPPCTQSVLDANFLNNNYTTES
AAAAAGFDTPSGGLAGDWYIINVPKTTTFSGVTTTVKATDNNGTSANANFVVFPQSAAAVNDGSNTTL
AVNYTADPLMADAVQGVAPATTFAAVTPAIRAGFYDLPDLSTPYVVAPGTIAIGDTNSVPAPVDQAAALT

NAIAVTSIYNQYAVEPGISAQTDWVFSMPTRRYEVAVNYSGNQRVFSPAPSSLSVARFTSSNTFMSNVVP
YEVCVNADGQSFFDREETGKTNSAVFSPSQLQSYSFCGETSVLSFGTGKVLGAQVAASSVTPGFLNGWGI
MNTQNGPGKQGLPIIGASFIKLVNGAAQPGVSGTYGISFPHRYTK
>WP_106683308.1 cell surface protein [Melaminivora suipulveris]
MKKNVLALSIAAMIGGLGFAGAASAQTVDTLTVNESGLGHMLLVPYYTAQNNNMTVFHMVNTDSVNG
KAMKVRFRGAANSDDVLDFQVFLSPGDVWTGAVVKGGDADGRAQLVTADGSCTLPAIAKNEPVNFIYD
RLPSWMDGAAKANSTREGYIEIFNMADVKSGSDLFKATKHVNGVAPCTDAALSATLMMEEATPGLAAP
TGGLMGSWYIMNVPSTLTFGGSTTNVLATGATRNVFSAQANGAPRSGLFSADPLFKNGLLETQKFDMP
DFSTPFLGVTPTTQTGADTQANALTQLLARTAVMNQYAQDANISAKTDWVFSMPTRRYNVAANYNVTP
GNAGYRVYNSAVGSFFTAGNTSVNATAGDANYGQICLSSTTQIFLDREETAKTTGAVFSPGNVTKLPLCGE
VSVMSFASGSVLGAAVAKTAASGTYTNGWGRINFAEPGIPVLGGAYISLRNPSASAGVSGNYGLLWPHSY
ISALPPK
>WP_040435241.1 hypothetical protein [Melaminivora alkalimesophila]
MKKNVLALSIAAAVVGFAGSAHAVTNLTGADATTLRFGNTGTGHMLVVPYYSAQAGNSTLLSIINTDEKN
GKAVKVRFRGAANSDDVYDFQVFLSPGDVWTANVSADPNGKAYLTTHDNSCTKPAKNTLNTTPFVTARL
NGDEDVRANNTREGYIEIFNMADIPSTAALFPNIKHSNGVAPCSGTAWTLLNTDTDNATLTGPTIGLAAPT
TGLAANWTIINVPKSVAWGGQAAAIEAVDAAGKPAKGNVVYFPQTSTAITATQAAAFTADPLMQGATPV
VKGALYDLPDMSTPYTNIAAGTPSQQADLLAKAIEATAVMNEYWTEPTIFAATDWVFSMPTRRYAVAVDY
KAVGGPAPVYNADNERHFTNANTQMSEDKLCVTDITSSPWDREERQPAAPDEVVISPSEPGKPVLFCGE
TSVLSFNNSGATTSGVLGANVAVKDIDLAYTNGWLKLVTPGVAANSGLPILGQAFVSAYNPDVGAGTSGN
FNTGWSHRYIRP
>WP_122239414.1 hypothetical protein [Franklinella schreckenbergeri]
MKKNVFALSVATALAALAGSANAQVPFIQPDAGTVSAGNVVATEFRVNDGGIGHVLLQPYYTVQGARNT
LLNVTNTDTKNGKAVKVRFRGARNSDDVFDFYVFLSPGDVWRSRVYRDGERTYLDSSDTSCTLPGADVIK
NTAFKTVRVFNDLESEVREGYVELLTAADIPPGSALYKSIKHDRMTGKVSCNLPQLIDSTVRLTDLDTARKN
GLDFPTAGLVGQWAIVDVSKNNAVSGNATALVATDAKGVPGAGSIVVAPQDSVVAPLAWTDVTSPVLGT
TDPLLTKGSVQAQNFDFPDLSTPYVKYAPTTRTPAGHQANELSAALAVSKVVNDFSTGMGVDLKTDWVF
SMPTRRYGVAIDYATNTPVENSESAFFQLAGGANRAGNVRFEVENGIPHLSIALNGSYFDNNERQLQPEV
SPARIQRLAGEVNVLTFNGNPADSVLGAQIAAQRVNPKLGTESIAEGWASISLENSVALANGYRSALPVVG
YASFTAGSAALGFTFPHRYSR
>MBP7606356.1 cell surface protein [Giesbergeria sp.]
VVGAAPLTTTNAQQLQFAEGGIGHALLFPYYNAQNGNMTVLNVVNTDTSNGKAVKVRFRGALNSDDVL
DFQVYMSPGDVWTAAVSAGSDGVAQIITNDGSCTLPKLNKGVGQSFVQDRLNGNLSVADKANNTREGY
VEIFNMADVPPTLRGANTTNPLFTAIKHVNGVAPCTDAALNGTLVSLTSPAAVAAAGFDTPSTGLVGDYYII
NVAQTTTFSGGATAIRALTNTTGAGVNARGNFVHFPQSASTANNVDNYTADPLFRSANVTDAAGVAVTS
PKIAAAANYDLPDMSTPYFTAPGANAPLVQARELTDALAVFSVVNQYANDGSINAKTDWVFSMPTRRYNV
AANYAATTTTAADYRLFTNLPVNRFTAGNTAVVNGAICVNSDGTTFYDREETSKVDGAVFSPGTAVKTRFC
GEASVLSFADSGVSVLGGSVARQNVSGVYVNGWANVNTSNGGLGLPILGSAFIKLSNPQATSGMSGTYG
ISWPHRFNRFQ
>WP_280581151.1 hypothetical protein [Lampropedia aestuarii]
MAWFYVHGRKKRCFIVKNYRISLVEPFLRQVFMLRFRSVIETWSLRFQTFPFWSLHMKKNVFALSVAVAL
AGLAGAANAQEVPFLEGNTVTASDADSFVVNPDGIGHIQLVPYYSTQGSRNTLLNIINTDSDEGKVVKVR
FRGARNSDDVFDFYVFLSPGDVWRARVYRDGETTKLESADNSCTWPLNFAADGGSAFQTSRVFNNLQT
EVREGYIEILTAADIPKNADKDSLYTAIKHVKGVAPCTEAVIEASMQPLTTPPAALAAGFANPSTGLIAQWAI
SDVETKATTSGVATALLAVKGVGKTPALGNIVVAPQTNDAAPATWSAPAGAAIGTADPLFTFDTRDTSYFD
YPDLSTPYLGNIAPAAQANAVSSALAVTEVINEFATVDADLQTDWVFSLPTRRYGVTIDYTGGANGRPIAR
VNGDSEYFGGLTQAALDTTGAAPAIVINVETKLFDDEENTASSSAIVSPGTPVRSSIRGEVNVFSFNKAPED

SLLGAVIAAQNMKTTIGSNVVKSGWATVGLKEQGTVTAGNNTGLPVIGYATVGSAGNAMSFTWPHRYN
RAVK
>MBP7535426.1 cell surface protein [Ottowia sp.]
MKRNAFALSVAAAIGAFGLAGVATAGVIPGGTFNITGTVGPVAGTARAVGANALVPGRFKVGHILLGEYFT
TQNGNATMLHITNTDQVNGKAVKVRFRSAQNSDDVFDFTILMSPGDMWTGGIVADEATGRSKMITAD
TSCTLPPVVNGELFPTDRLPSSKTDAEKASMTREGYIEVLNMANIPSDKVAPGKEVYDSNRDSAGKTGALY
RATKHSAGKPTCDSAVINTLRTNPTDMQAAADMGLDLPSGGLFANVNIINVPNTASYANQAAAVVAAA
AGVSSTGNIVFYPQIGGDAGNVDAATSDPLLRESAVLNSGKLALSPAALPLIKAQWFDLPDLSTPYITGLAT
GNDAPLDQANKLTTAMNSSSVINEFNVDPSLAGATDWVFSMPTRRYHVAMDYDHAGRGLTGFDGRVF
TSTTVALGSDTNTAANNTFNTQFNATNTALDTDKIKICVNSAGITMFDREETTKTSGFVISPGTASTVRFCG
ETSVLSFNLDGSTADPSVLGSKETLQIIGTPYTAGWAALATAGNGSPVIGSAYMRLTNAGARAGVSGNYGL
VYPHR
>WP_180549957.1 hypothetical protein [Ottowia beijingensis]
MKKSILALGAAVALGGLGVAGSAHAVAYFGAGNTVGHTTLAASTALSLNPGATGHMLFTPYFTAQGNMG
TLFNITNTDLVRGKAVKVRFRGAANSDDVLDFTVFLSPGDVWTGSVAKDAATGRAAISTSDKSCTIPDATA
FPGVFKTDRLASYLAADVKNANVNEGYIEVLNMADIPPFLDMVAGEGGTKANPLFTNIKHKAGVAPCDN
LAFQTVLSTDIVDAAGAEAAGLFEPTGGLMGSWAVFNQSELAVYSGNQTAVVAHAGWGQDNAQTPLS
ARGRIIFAPQVGSPVGVVVDGVTADPLLRGAAPLIQPLWFDLPDMSTPLDQAVAGAIDQARNLSTALWR
SAVYNDYVATAAGAAVPMSTDWVVSQPTRRYHAAVNYGASASASALVWNADMANNATPTTTTAPTLA
QNMYAPLSLNKTLAMGPQACIMLNFSSAGREEQFQVAGGGFSPGVSLPNCGEVFTVSFGATSVLQGAV
TNTRVNPVSTEGWARLALGAGRLPMVGFAATSIVNTATNGNYGLTLPHRWVD
>WP_028602012.1 hypothetical protein [Ottowia thiooxydans]
MKKSLLALGAVAALGGLGFSGAAHAVWVAQDVALAPAQTLSASTDTGVGHILVTPYFNTANQTGTLLSVV
NTDTLNGKAVKVRFRGAANSDDILDFTVLLSPGDVWTASVTEGSDGYSRLITPDTSCVLPGIPNAANTGG
EGQPFTPTRLDPKLTAEAKAAHTQEGYVEWLTMADIRKGSTLYAAVKHKNGVAPCTQTEMDFLMDPYST
MNTTEASAYGLTAPTGGLMGNWTIYNSANVTSYGGPNSAIVALQSQEAGPAVPSTARLFFAPQIGASAPN
FPTDPDLVWSQEILTGTRTHLAETADPLITTVGNGGSKLVEILPFDLPDLSTPYVSGYQYAPTQSGALSASLA
TTAVMNEWTSGTSNGVEFSTDWVFSQPTRRYHAVVNYGGLSGTPSAVYNTQSSVFYYGGGTTIKTRKFG
DVQYGSMLCLKDGLAKYNAFNREEGEITIRISGEWSPGTPAGETQGFCGEVAVLTFGDSMNRALNASLTT
NNIPATSLPAGGAGWMAVSLPPAVTIGTPPRTYAGGLPVIGYAANTFKAGSGAGAGNFGDAITHRYKRP
SGPAAEIVPPVVTP
>MBY4595853.1 hypothetical protein [Ottowia caeni]
MKKSLLALGAVAALGGLGFSGAANAVWVVDSAANATAQTLSASTDTGVGHILVTPYFSTAGSTGTLLTVV
NTDTTNGKAVKVRFRGAANSDDVLDFTVLLSPGDVWSASVAQGADGFSRMSSPDTSCVLPAQPTGEGV
AFSDARLDTRLTAEARAAHTQEGYVEWLTMADIRPGTRLFTATKHKLVGGQMVAPCTSDVMAELMDPT
TTMNVDEASAFGLTAPTGGLMGNWTIYNSGNVTSYGGANSAIVALDTTAVPPAPASARLFFAPQIGASNP
TFPPNGGDVAWSTGTHLGETADPLLTNFDNGGPAGGPILQMLPFDLPDLSTPYVSGFGFAAAQSTALSAA
MATSAVMNEWTAGSDNGVNFSTDWVFSQPTRRYHAAVNYGTGGATPAAVYNSLSSPFFYPPASMVKK
TLGANGAFGTVLCLQDGIATYHAYNREEATIVPTFNGEWSPGTPVGKTPGFCGEVAVLTFGEQSKALNAQ
LTTYQIGSLPSGGAGWMSVGLPGVTTIGEATYAGGLPVIGYAANTFNTSVGSMVGNYGDAVTHRYKRPS
GPAPAIVLPAPAVE
>WP_106847399.1 cell surface protein [Pulveribacter suum]
MKKNVMALSIAAMIGGLGFAGAASAAVITGTTGATNAAASIQAGEMAKATATSLTLAEGGVGNALIVPYF
TTQNGNMSVFHVTNTDTDNGKVVKVRFRSAANSDDILDFQLFLSPGDVWTAAVLADENGLSQLVTSDN
TCTVPHIPKNTFIPFRKGNLPAFAADQNQLTREGYVEIFNIADITNVKTWDAAGAVSATGTQFSPLYKATKH
VNGVAPCSAAGEARTLLDDLAVTNFTEPTAVAAGLQTPSGGLMGDWYIQNIAQSTTFAGVTTTITANGR
ANFTHFPQVAQPATATANDVTADALFRTTFVRDAVGAPVTTAAKTLLDEDVPDLSTPMLPANAVAEGARA

QASDLLAALAVTSVTNQYALNAPVQGKTDWVFSMPARRYNVVANYGAVSGASGASGPTAANAKYRLFT
DLSAAATQDDNWFYTAVNAAAAPAGYGAGGTGQKAAGNTTVDAATGNICVYADGQKFWDREET
TGTTGPTFSPGSTTKASFCGEVSVLAFNDTGKSVLGAEVARSNTTLGYVSGWGRLDTTNADMGLPLTGAS
FIKLTNPSAAAGMMGTYGITWPHRFTK
>WP_275743087.1 cell surface protein [Ramlibacter sp. H39-3-26]
MRKNVLAKSIAAAVAGLGLVSGAYAITVADISGTATDLRLSTSGTGHSLFVPYFTTQSGNSTLLSLVNTDTTN
GKAVKVRFRGASNSDDIFDFQVFLSPGDVWTANISANSDGLSYLTTADNTCTKPSKATLNSTPFITSRLNPL
ATTAAKAAGTREGYVEIFNMADIPPAAALFPAIKHVAGVAPCGSATGTALTAWTSLDTNASLATYTGATIGL
ANPTTGLLANYTYINVPKALSWTGEAQAVEAVDATPALATGNIVYFPQSAAPVPVAAAVGFTADPLLATGI
VVAGMYDLPDMSTPYAGGVGVTPSAQAINLTAAIAATSVINEFVTEPGIFAETDWVFSMPTRRYAAAVKY
APPISIVQNPLNITYFDVATNLTLTGDQICVTGLSTLPAGDGATLNQEEGSLASSEEVVISPGLPAAPLTFCGE
DSVLSFNSGLSESDPTLVLGATVAKKAIDLPYTNGWMKIATPGATPGVGLPVIGKSFISAFNPSVSAGTAGN
FGMGWAHRYTRP
>ODU10753.1 MAG: hypothetical protein ABS84_01910 [Rubrivivax sp. SCN 71-131]
MKRTLVAQCAAAVLTGAAFVGANAAVVAPGAGAVDGVQTATQLVPNKDGIGHIVIVPYYSAASGNDTYL
SITNTDMRNGKAVKVRFRGASNSDDVFDFTLLMSPGDVWTAAITKDATTGKAKLLTGDTSCTIPNALSGS
TFVTGRLAPYQAADAQAKETLEGYVEILNMADIYRGAVPAVGAGSPTRAAAVAPAVNNALYGATKHVNG
VPPGCGETSPAIAALANNPTDYNATLTGAVQLGLDVPTGGLTATWTIINVPLATSFSGVGDAVEARDAATG
LPGWGNVVFSPQLATPVGLAAARLATADPLLRGAVPDSETVVAPAVARGAPVLPTAATSYVAAAMYDFP
DLSTPYLQGSIAAMANGVATRLQASLLTDAYAVTSVMNEFVSDASLLAKTDWVFSSATRRYNVARNYGTA
ATNASTVFTDLEWDDVNTAGGVTVGGATTANRYAVSNTTAEGNLICVTGVGTAAGTFGAVPDNKTLAVN
ADREETFVGSTSQFVISPGVPTAPLTICGEVSVLTFNNAGGGGALGAQLTKKDLTGTNVNGWARLSSPGLA
GVAPGTVSARLGLPIVGGSFMQLTNGAVAAGTSGNYGLTFPHRTTRY
>WP_119109288.1 cell surface protein [Simplicispira hankyongi]
MKKNVLALSIAAMIGGLGFVGSASAALTVAESGAGHMLLVPYYTAQNGNMTVLHVVNTDTAHGKAMK
VRFRGASNSDDVLDFQVFMSPGDVWTAAVTQGSDGVAQLVTADNTCTLPSISSGVPVSFVQDRLSPALN
AADKANNTREGYVEIFNMADIDNAAIYVPPLNTSQAPLYVATKHVNGVAPCTSSVLNGTLTTGAATTGLL
APTTGLTGSWYIINVPETTTYAGAMTAIKSDAATNNVFSPQATGATALLSADPLFDAGVLVKQAYDVPDLST
PYDATYATASAQATALTTTLAAQSVINQYANDASITAKTDWVFSMPTRRYNIAANYAAPISQASASTTEAG
VITYNGGATAATAYRYINSDVALFFSNVANTSVNSIGQICTSATGQVFYDREETSKTSGAVFSPGTISKTQLC
GEVSVLSFADAGMSVLGSSVARSNVSGVYTNGWGQVNFAAPGVPVIGASFIKLSNPNANAGTSGTYGIT
WPHAKK
>WP_027995450.1 hypothetical protein [Simplicispira psychrophila]
MRKNLLALSIAAMVGGLSGAANAAVFVNTVGAAANGLPAISAAAPLATQLTSTTTGIGHILVVPYFSTQQ
NNNTLLNLVNTDTVNGKAVKLRFRGAANSDDVFDISIFLSPGDMWTANVSADGDLSRLATSDTSCTLPSA
QDIKDLGGKFKTNRVRNADPLQTREGYVEILNMADVPPGTGTAGANSALYTAIKHVNGVAPCTQALMDL
QENDLVAIDGDVNSPVRRGYSWPTGGLMANWVIVNVGDSASFSGEAVALRATPSGTIDTSGSGNLVWF
PQTTATPTMAANLLTADPLLSSGAVIAASYDFPDLSTPYVSGALGASPAAQASELASTLAVRSVTNEYLTNK
SVNFATDWVFSMPTRRYAAAYNYGATGSAQRVLNPLVSAHFNSDNVTLDAAKGQLCISTGTMRAFDREE
NTRTTFVISPASAVSFCGETSVLSFNSAAYSVLGAKIAQQNIETKFADGWFSINTPGVGNGLPVIGFAAAKT
RGTNLGGTWKHRYSR
>WP_157983670.1 cell surface protein [Simplicispira metamorpha]
MKKNLLALSIAAMVGGLSGVANAQAVISDTLGARVVSNTAAFGNLAAPAAGTAATVLTPTSTGVGHILFV
PYFTTQGNNMTLLNLVNTDTINGKAVKLRFRGASNSDDLFDISIFLSPGDVWTANVSANGELSALSTSDTT
CTLPSIEQIKETAGKFSTNRVLNNAAAETREGYVEILNMADIPPNTIGAGRTNSALYTAVKHVNSKAPCTQA
VMDLQANPLVAGDVVNDPVARGYSWPTGGLTANWTIVNVASKASFSGEAVALRATPTNTIDVPGAANL
VFSPQTGNTVANAAALTADPLLKTGLVRAASYDFPDLSTPYTTPLVAGAPEAQANQLAGVLATRSISNEYV

TDPAVSFATDWVFSMPTRRYAVALNYETKTIVGHGVAGYTAAPASNFFSAAVGGNASLNRDKTRICVDAG
PLTAFDREENSRTSFVISPDEILKFCGETSVLAFNGKASVLSAKLAVNDIATRFTDGWLRIGTNGLGNGLPV
VGYAVGKAIGPDGGNFGGTWTHRTQP
>MBX3689854.1 hypothetical protein [Dokdonella sp.]
MALLLPDLGLACAVSIDFRPVQGGGKSKKPCGQKFRSVAMKKNSLTMAVVAGIAGVAGFASLASAVELNP
DGLGQVLIYPYFTVNKGQDTLVSVVNTDDVNAKIVKVRFLEGYNSREVLDFNLYLSPNDVWTGAVVQLTD
EDGGAGIKTFDNSCTYPLFKAGVPQAFRTYTFDGTIGPKDGAPTSITRTREGYLEMIQMGDIPPNTDLFDT
VLHDSTGHPECDTSLIGNSVIANTDVVAPTGTLFGSGAIVNVGQGTFFGYDADAIDGFFISPYVTRSGDTLP
SLQSYDNFTSYIFNAGALLTLDYSAYTTGADAVSALYQSDTLYNEYLTAPGIGAATDWVVTFPTKRFYVDPFY
VGAGPAIAPFVQVFNKVSNVEVILQQYDQEEQTIIPQDCDPSNPTPGSDCDWSPSPPVDVEPSSLPYEVN
VISYLDPADPNAGVSSGVLGSKLTFNVPPFASAGWIRLDLASGNSGTHVMRAAA
NGTQLRGLPATGFEVYNVINSNVSGGVMANYSGLFRHRASRSCAAGPCS
>WP_182531496.1 hypothetical protein [Dokdonella fugitiva]
MKRNSLTTAVVAGIAGVAGFAGLANAVDLNPDGVGQVLIYPYFTVNKGQDTLLSVVNTADVGKAVKVRF
LEGYNSREVLDFNLFLSPHDVWTAAVTQVSDDGGAQLITSDHSCTDTITNPQPFLPYAYDGSLAPAQPAD
NGPQDITRTREGYVEIITMGDIIPGSDLDVVTTHVQNGTPDAGTPDCGAPVGNDTATAADLQTPTSGLFG
SGAVANVSVGTYFAYNADAIEGFYDNPTGNLYTPAGSLLPSLQSASNAAVPGGAQAFLFLNNGQLLTANY
ARGLDAVSAVFMADAVYNEYFVDDVNFGAASDWVLTFPTKRFYVDKYVYPFAITAPFVEPFDNTADGES
RVNLAIVTYDREELNSVVNVPRCPSPVNPVTCFSQSPFLGHEVNVLSFLGPSTATPSESTVLGSNLFKTVNA
VGLDGWASLDLFSGDGGHVLAGGTLAGGGAVNLNGLPVTGFYAQNVINTNANPGLLANYSGVWRHRA
HRSCSANGADPACS
>MCP5473353.1 hypothetical protein [Rhodanobacteraceae bacterium]
MKKNTMATAIVAGLAGVAGIANISTAVNLNPDGVGQVLIYPYYTVNGGNTTVMSVVNTTSAGKAVKVRF
LDARNSREVLDFNLYLSEYDVWTAGLFSLADSGPGNIVTTDTSCTVPGIEDGIFLLPTLADGRRYFPFRTSAF
TDFSAAGLNTGAFTRTRDGYIEIIEMGSIPTNSTFGSALTHSNGRPGSCAFLEAAWLGSGSPGANGIWVQ
DPFFDLEPPSGGLFGGAAIVDVADGTYLSYNAEAIDGFSASIQHTAPGSQLPNLASVNSTTPGNVTSYVFD
RGRLITSNWSTAGGGGFRAVSALFMREAVFNEYELDENLGAGTEWVVTFPTRKFHVTGSTTFSAPFTNG
YSGCERVSSRIYDREENTFALFDFSPGAGQQQVCWEANPLWFSKTPLSSGSATPILGAPGAAGSVGSLWT
GIPTYLEVFGIIQRTFTNGWFWLGFYDENAINGQGIPTPLTRAPLVETNPGAGNTPDSYYGLPAVGFWALR
VVNVNQGAGLQASYAGAYPHRASRACFKGAYGTAPCD
>WP_067644292.1 hypothetical protein [Dokdonella koreensis]
MKKNSLTTAVIAGIAGVAGFASLANAVDLNPDGLGQVLLYPYYTVEKGQQTYLSVVNTTSTGKAVKVRFLE
GYNSREVLDFNLWLSRYDVWTATIFALDDVFDDQGDGAAILTRDKSCTSPMFSEGDGTVGGAPYTKFRSF
AYAGDGGPQTITRTREGHFEIIEMATLTGPTNSAITHNNGNTPGGCGTVRNLNAANADMSAPSGGLFGG
GAVANVAQGTYFSFNADALEGWRAAPLFTGTDNLRPALDDAIDASGDSATAFVALGTSISTSVYAANRAID
AVSATLTANAIYNEYVVSPAIGANSDWVVTFPTKRFYVDPLMVVAPAVAPVQPFVQRFTAPGVSCVEVGIS
IYDREEVTTTAGSSGWSPPPPGAPPSSLCRETNVISFLDVTTAPTASGVLGSKLVTNIRPTVGAEGWVKLNL
NPGSEPHALRASLNGNVYHGLPVTGFWANNLVNENVSDGVMSNYSGVYRHRASRSCTAGESTCS
>MCI1710691.1 hypothetical protein [Chiayiivirga sp.]
MKKSSLTTAVVAGLAGVAGLANVSNAVNLNPDGLGQVLIYPYYGVEGGNATLISVVNTTDSVKAVKVRFL
EALNSREVLDFNLYLSPFDVWTGAVSAGATGPGRLTTSDKSCTVPTIPAAGVDFRNFAYTGSFDDDGPNAL
ERTREGHLEMIEMGTVLDTNASFNVATSFYDAAIHIDGTPLGCARLNSSWSVGGQWFNNGTFGANCST
ANCGVDNSVQGGLFGGGDIADVANGTSISYNADAVEGFYTLATTTLHTNPGFTFPSLSNAQTSALGEADS
VIFSPTSPLGVVTLDFATGRPNAVSSVFMHDAIYNEYNTTDGLLAASEWVITFPTKRLHIEQTTFDRRRPFT
DNVDDSDGAGQVTPAGADSVDTLVFDPSGSCEPISLVFFDREEGPEAAIPVVDFSPQPEGEDVGFALCYET
NVLTFNQEAAVTAGASEVLGAKTIANNVNLANASGTEVTTGWVGIGLANDVDGDLAIDQFMLDNNGSA
VTNRNIQFGLPVTGFWAANYVNTAAQPGLLANFAGTHKHRGSRFARTATVTNEGSATGETIVVTGFAAS

>PZQ19592.1 hypothetical protein DI564_02495 [Rhodanobacter denitrificans]
MKKRNGSRSFHRLAASIAVACGLAAAGQAGAVHLDPRGSGQVLLYPYYTVNKGQQTYVTVLNVSNRTK
MADVKFREGYNGRTVMDFKLFLAPFDTWTGTVFALEDIAGEGSEGAAILTADRSCTAPSFWTEGHGMLG
GAPYARFSSNNFVDDGGPAGIARTREGHIEIIAMADLTGSLAAAVELRSGVPVDCSRVRGLAYGTEEALVP
TGGLVGSGAVINVGQGTYFSYRADALADFTKRPLLTGLGELEFLDHANDGPGANVATARVPVDGQWVEA
PYRLQESIDAVSAVLMADSLFNEYVVQAAIGANSDWVVTFPTKRFYTDLSVMSGTVAIAPFIELFGENGSC
VTTRVRYLDRESQTVADSGFPGAPPIPTRPRLCHVTNVLAFLGDTNAEESAVLGSRLVAQGFALDRAFSAG
WARLDLNPEEQPHAMRASTSIGGAGSSGKVFHGLPAIGFWATNLVNNNVADGVMSNYSAALSHGSSVS
CSAGTSACN
>WP_157578530.1 hypothetical protein [Rudaea cellulosilytica]
MKRTSLTTAVIAGIAGVAGISNMASAVYLNNDGLGQVLIYPYYTVNGGNNTLLTVVNTTNQGKAVKVRFL
EAYDSREVLDFNLYLSPQDVWTAAVVPVGGGAGVFTNDNSCTVPALPTSLATAQPFLTYAYDGTGDTAAD
GGPTGVSRTLEGYVELIEMGTVTNASQDTLKSITHSAGVPLDCTQVADAWNGGYWDITAGGDVTIDLSA
TSGGLFGSGAIINVNQGTIAGYNADAIDQFYSDTFSHHTAPGSLSPNISSATSHVSYVFAPSPTTTSPTLITTS
YTSGVDAVSSLFMADKIFNEYWTAGGTAANSEWVITFPTKRFYVDSASAAAGHLQPFDKLFSKGVSCAPI
GIGIYDREERTTSGTIGFSPAKSQQGAALCYEAQVVTFNQPGIGTAASAVLGSNLTANIAPPAGANNGWA
VIDLYGKSAANHVLPAAYTDGTVGAANVFRGLPVTGFWVANLVNGNVGGVLSNYSALFRHKLHRTCAQA
SGSACS
>WP_137914996.1 hypothetical protein [Rudaea sp. 3F27F6]
MKRNLGTFFFGAAASLCAPAAMAMYLNPYGAGQVLVYPYYTVNGGYATFFAVFNTTNQGKAIKVRLLEG
YNGRDVQDFNLYLSPDDYWVGAVVDSGNGGAAIFTNDNSCTVPKLPRTSATALALTTANFDGSAMQGK
DGGPTDVSRTREGHIEIIEMGTVTGPSATLNAITHVEGVPADCASAVNAWAAGGQWVADSTKDIGPPTG
GLVGNGMVLNVANGTVFSYGADAIAQFYVKDGRGEHSRPDALTPNVSNATSLSADVMTDAGRLTLAFA
RPIDAVSAVFMANEIHNEYWTSNSVAAASEWVITYPTKRFYVDPYYINGAVRPPFELAFSKALGGTSGSAI
RAAIFDREEGQNTPEIVTLPPVWGKGLFYETQVATFGQQQSASQIVASRLVTANFQIPDAENGWAKFDLA
MPEATTHRLAAVNGNVLIGQPVTGFWINQLINGDAGGKGVLANYTSLYRHKLHAACLSADGTPCS
>WP_212350137.1 hypothetical protein [Azoarcus sp. L1K30]
MNKKILALSVAAAIGGISTSASAALTFNADGVGHILIQPYFTAQEGNSTLINLVNTDTVRGKAVKVRFRSAG
NSDDVLDFQVFLSPGDVWTANVSEGADGKAVLSTADKTCTVPAIPAGGVSFITNNLPPSATDAEKATWTR
EGYVEFLNMGDIVKDADTTTAAYKLYATTKHVAGVAPCDTAILNDPTKVEPGLSAPTSGLMGNWTIINVPS
AASWAGESAALVSAGAPTNVVYFPQDSNQYFPAVSVNMHTADPLLIKADKVANFDLPDLSTPYDGDYAN
ATAQAAALTTALAVSSVTNEFLTNAAIAAETDWVFSMPTRRYHVAYDYATTVAAEKRVFNVAGAAVTTAP
VASWFYSSNTEVSGNLICVKLGGVTNFDQEEGTATAGVVFSPGTIETVKFCGEGTVVKFNGSNVLGASVAS
GSLSTGSTAGWTTINTANGGFGLPVLGQSFVQATNSAVAAGVSGNFGAGWAHRVTRP
>WP_148578221.1 hypothetical protein [Zoogloea oleivorans]
MKKSLLAVGVAATLGALSGVSNAAMTVAQNGVGQINILPYYTVQNGNTTLISIANTDELNGKAVKVRFR
GAQFSDDVFDFQVFLSPADVWTAAVTLDGNVARLTTNDNSCTLPAKADLNQKFVTARLLDANKAQGTRE
GYVEIINMGDIPVSAVPDSLYVATKHVNGVAPCTTAVLIGDVAAPNDLTGKLTNPKTGLTSFATIINVANSKA
FTVPATALNQIDGVTGLPSLVVPVRYSRQANLTTGTLSFGSVSPAFASAQTADSVFDPATGNVKMYEFDLP
DLSTPYGTANAAAQLAELQTALAKGSVVTEYATDDSILASTDVVLSQPTRRYYYTWTDTPTAATDPLRTLAT
VDGATGVYSSLDADTNSVTVGAPSVYDREERTVSAENNIVISPNPQGSATWTLIGEVSVVSINNGAGKTG
ALGAEITAQDYTFAYNDGLVRLSTTAGVQALPVIGFTAVNVFNASAGGAAGTNYGQVLPLKWNTAP
>WP_123785262.1 hypothetical protein [Pseudazoarcus pumilus]
MIRNKLLAYGIAALGLGSGVAHAGAFGFLEGADAYGGALVVPYYTVQNGNVTLLNIVNVDPESGKAVKVR
FRGAERSDDVFDFQVFLSPGDMWTANISQGPDGRARLTTEDNSCTLPENVNRSFETLRLSGDEAARAAG
TREGYVEIINMGDVIDYGNDARTELFDAIKHVDGVAPCTPEILQSVVSGDTSQLNVDNDENGEAERLNDV
ATPFLYANVTILNLDEAAAWSMNAVPVDSLFTTIFSSEGLPETRYWSQTASKFDADLAQVTLDGVFLSGAV

DPAQYDFPDLSTPRYPDSSPIEQYLITSFYGIGAFPWLSNEFITDQEIFAATDWLFSMPTRRYAVEGRPYEG
GVVHLDTADFQGDDGRGVLFPFSEYYDADTGCVRVAGLEVQDREERILTPDVVVSPGTTVRPQLCGEVA
VMSFNRKGATRSGVLGAELTLNDITVPYENGHASFLFGGGIFDELDFELGYGLPVIAQAFVRANNGGSGA
ANMNFGGNWKHRGFSLPFGFPFFMGEP
>WP_110525088.1 hypothetical protein [Parazoarcus communis]
MKKKLLATGVAVALGALSGVASAAVTVNAGGVGQINVLPYYSVQEGNDTLISITNTDTTRGKAVKVRFRG
AEWSDDVFDFQIFLSPGDMWTGAVTKDGNLAKMSTSDSSCTLPASVNQAFVPIRLQAAAQNTGTLEGY
VEVITMADIPATGTGAGNLTANWAVTKDLYNAIKHVAGGKPACRTDATAQTLLEGLTQDNVLVPAGAPQN
AWMVAPTASLTTFATIINVPTSKAFTFPATALVESAPLKQYFEQSNNALAFNVALTADNIFAPIAANGGAELP
MYQFDMPDLSTPTNAVDVLAVDQRDAIATLLAKASVTTEYVTNDSLLASTDVVISQPVRRYFYEYTQKAA
GQTFHLTIPDSTGTNHNYDVNGDVGTVYQPLDGATNRIAVANPRFFDREENTFTSSSGIVISPTPPSAITPIS
LKGEVSVVSINNSGLPTGALNASITANDYTATGGYSDGWTTLSTTSQGTNGGALPVIGFSAINLFNASAGA
AGTNYGMTLPLK
>WP_146060740.1 hypothetical protein [Thauera chlorobenzoica]
MKRQLLASVIAGLGVVGSAHAVHVNPDGLGQVLLYPYYSVQDGNDTYVHVVNTTTAAKAVKVRFLEGK
NSQEVLDFNLYLSAKDEWTAVITRTETGAQLATTDTSCTAPAIPAGGIAFRNFEYKGDGGGDTLERTREGYL
EIIEMGDLDDVAPAIAAAATHTAAGVPADCAAVRAHASTASGTLPASGGLYGFNTLINVEAGLSSTVDAVA
LDDFWLNNEGTYTTTGSLSPSLEDGTQSADILDGNQVINAVFGQRIDAVSAVLSRTNVMNDYVVGLDFN
ALTDWVVTFPTKRFYVNGDLAPRDPFTQKWVGTSCDSISITYYDREEQREVGEDDFSPQPVAEGATLCNE
VNTISIVANGEEGGLLGAEFTAAQIELAAGFNAGWMDIGFNSDAAQDGLVSLNGVTVTGLPVIGFSAMSF
TNNTLVVDGVNVLSNYGGTSVHKGLRDITVVAPQ
>WP_247734156.1 hypothetical protein [Thauera aromatica]
MKRQLLASVIAGLGVVGSAHAVHVNPDGLGQVLLYPYYSVQDGYDTYVHVVNTTTAAKAVKVRFLEGKN
SQEVLDFNLYLSPKDEWTAVVTRTSTGAQLATSDTSCTAPAIPAGGVAFRNFEYSGDAVSTLERTREGYLEII
EMGDLSGAVAAAATHTSAGVPADCAAVRAHASTASGTLPASGGLYGFNTLINVEAGLSTTVDAVALDDF
WLNNQGSYTTTGSLSPSLEDGTPVADIIDGDQVITAFFAQRVDAVSAVLSRTNVMNDYVVGLDFNALTD
WVVTFPTKRFYVNGDAAPRRPFSEEWDGSQSCDNISITYYDREEQREVGEDDFSPQPDAAEAALCNEVN
TVSIVANGEEGGLLGANYTAAQIELAPGFNAGWMDIGFNSVAATIGLVSANGVRVAGLPVIGFSAMSFTN
STLVVDGVNVLSNYGGTSVHKGQRRITAVAP
>WP_068635735.1 hypothetical protein [Thauera butanivorans]
MKMVQKPRKSLVAFALASAIGGAMVSAPAQAVNLSPDNLGQVLVFPYYTAKNGFDSYIHLTNTSNTTVIA
KIRFREAKNSREVRDFNVILSPYDVWTAAVTQDGEGAKLVTYDKSCTSPLLPASSTSPGATEVDFTSLGYDG
SDQYAYDNGGLGLDRTQEGYFEVIAMGASSNQTSTYNSTTDNLIEYNAKHVNGLPRDCAIVDQQFAGKA
GNLSTDEAFDHFRFPGNVLKGFSTLINVASGQAVGVEPTVFANFREVGSVNTIIFPPGDLKPDLSDVDDAL
GANYVDDGGLLASVAGANPIDVVSALLMRRNVINEFTSTAAGTTQTDWVLTFPTKHNYTDNGGLGAPFL
VAVEPFDEVFTKYGPAPDYSIDMSRHDGKSCVDIGPTYYDREEATRTGSSTDFSPRPAGGRIELCNEVNVLS
FNNSNVFGSGVRFAINTSAVAQTGWMNLRMGPATIGSSASAPNEGRLAVGGEVGGVSDDFYMHGLPVI
GFGAVVRFNDAEAGNNRNYGVAEEHSFNRTVVVQ
>WP_095551576.1 hypothetical protein [Vandammella animalimorsus]
MKKNVFALSVAAALAGFAGTASAQVPFITATGPDAATHFYINAGGVGHILLQPYYTVQGNRNTLMNIINT
DTANGKAVKVRFRGARNSDDVFDFYVFLSPGDVWRTRLYRQGEDTHIELPDTSCTLPSRDVVASTPFKVD
RVYDQKDVREVREGYVELLTAADIIPGSDLFKAIKHDSVTGKVSCNPSVIEGAMVALTNEDAARAKGLTFPT
AGITGHWALTDVTTDSTNTGNLTAVAAGYTPATAPGVAASYVFGKGNLVVAPQNEHAAPMAWRTTGTS
DPLLSSGIVPAQFFDFPDLSTPYLNGGTPVDQANHLSEGLAQKSIINEFVVDMGVDFRTDWVVSMPTRR
YGVAIDYRGQGAAVYATGNHYFDASNTEFKMVGGVPQVAVTTMTQTYHDDNERTLRATVSPGTLPRLA
GEVSVLTFHADAKDSVLGAVISTQRVNPMYNIEKINAGWGELSLSGLNGRGLPVIGYAALQAGGKALGFT
WPHASKR

>WP_011808472.1 hypothetical protein [Verminephrobacter eiseniae]
MKKNVLSLSIATMIGSLALSGAASAGVAYGTAAAASTAVTLPATSATDLKVNPNGIGHILLVPYYSVQEGNG
TLLSIVNTDTKNGKAVKVRFRGASNSDDVFDFTLLMSPGDVWTANVSKDAASGKAQLFTPDNSCTLPSS
AALNSTKFMTARVYGADEAARALETLEGYVEIFNMADIPPKVPATAGEIAQNGTAAIDNPLFTAIKHAPSG
DNAGKPACGSGLDKLRSDPTLSNISASTGTAAETDRTNLLKHGLWAPTTGLTGSWTILNIYGASVGWSGN
MTAIEARNGTDAPAPGRVVFSAQKSDRVPAADVNLLTADPLLRNKGGATPGTGIIPAVQFDLPDMSTPYLL
YGTNASIEAENATAIDLAARGSAPIRQAEALTESLAVQHVINEYITNPKISAATEWVFSMPTRRYAVGVDYT
STATMENPIFNGVSQGVAANAVRGPSDFFTSTNTKMGADGAPKYQACVEPGGDLFVYNQEEAKKDSG
GFVISPGVAGKSVNFCGEVSVLGFSTTQALKAKIATKDIDTGGFTSGWMDINTRGLEVANSNPKTYRGLPII
GSAFVKMTGPIAAGKSTNFSITAPHRFSK
>WP_239233986.1 hypothetical protein [Candidatus Nitrotoga sp. BS]
MQKFKRKSLYLALVAAVSSVGIANTASAAVNVNSNGLGEVLIYPYYTTRAGMDTYLSVVNTTNSSKAVKVR
FTEGKNSREVLDFNLYLSPNDMWTAAVVNTTNGAKLVTADKSCTAPQIPANGKEFVNFAFSGAALEGIQQ
SGGDGETQSLDRTREGYFEIIEMGTITNTAINAAITHVNGVPANCAVVQAATMNMGVGSALVVGGQSAI
ANPSTGGLAGTASLIGVAAGTDFGYDPVALDAFAPNNVQNIWNAPGSIFPDLTFANTTSVIFNHGSPVTST
WLSGDATVSALLMHNNIINEYVLDNATLSGTDWVITMPTKRYNVPVHNPAVSPDTTQLFSPFTHKFWLN
GACEPVGLSYWDREEGNVSIVDFSPPAPGGGTSLCWETTIVTFNNSHVLGSVNEVNVPVNFENGWLRM
AFNATGISVTNGQVDGNNVTHGTASQSLTSVDGDTYIGLPTVGFMVQDFINQNAAPGVMATYGGNFN
HKYTTSISED
>WP_239288550.1 hypothetical protein [Candidatus Nitrotoga sp. 1052]
MQKFKRKSLYLALVAAVSSVGIANTASAAVNINSNGLGQVLIYPYYTTRAGTDTYLSVVNTTASSKAVKVRF
TEGKNSREVLDFNLYLSPHDMWTAAVVNTGTGAKLVTADKSCTAPAIPAGGKEFVNFAYSGANLEGIQQS
GGDGETQSLDRTREGYFEIIEMGTITNTAINTAVTHVSGVPANCAVVQAASMDMGPSSVLIVGGQSARA
NPSTGGLAGTASLIGVAGGTDFGYDPVALDNFAPPQVQNIWNPPGSIFPDLTFADLTSVVFNNGGTVTST
WLSGDSTVSALLMHNNIINEFVLDNATLSGTDWVVTMPTKRYNVPVHNPALSGDATQLFSPFTHKFWL
NGACEPVSLSYWDREEGNITVRDFSPPAPGGGTALCWETTVVTFNDSHLLGSVNEVNVPVNFQNGWLR
MAFNATGIVVANGQTDGNGLNNPTEASQLLTSFDGDTYIGLPTVGFMLQDFINQNAAPGILATYGGNFV
HKYTTAISAVN
>WP_173053945.1 hypothetical protein [Candidatus Nitrotoga sp. AM1P]
MQKFKRKSLYLALVAAVSSVGIANTASADVNVNSNGLGQVLIYPYYTTRAGMDTYMSVVNTTNSSKAVK
VRFTEGKNSREVLDFNLYLSPNDMWTAAIVNTANGAKLVTADKSCTAPAIPAGGKEFVNFAYSGAALEGIQ
QSGGDGETQSLDRTREGYFEIIEMGTITNTAINAAITHVAGTPANCSVVQASNMDMGRLSSLVVGNQSA
RANPSSGGLAGTASLIGVAGGTDFGYDPVALDAFAPSNVQNIWNPPGSIFPDLTFAALSSVVFSNGGTVTS
NWSSGDNTVSALLMHNNIINEFVLDNATLSGTDWVVTMPTKRYNVPVHNPALSTDATLLYSPFTHKFWL
NGACEPVGLSYWNREEGNVATVDFSPPAPGGGTALCWETTVVTFNNSHVLGSVNEVNVPVNFENGWL
RMAFNATGITVANGQTDGNGVNHPTAAHSLTSIDGDTYLGLPTVGFMLQDFINQNAAPGVLATYGGNF
NHKYTTAISGNI
>SPS04664.1 conserved exported protein of unknown function [Candidatus Nitrotoga fabula]
MKNFKRKSIHLALAAGIGASSMAMAESPEGRGMYINADGTGQVLLYPYYTTRGGMDTYMSVINTTGYA
KAVKVRFIESRNSREVLDFNLYMSPYDMWTAAVVDTGTGAKVVTADKSCTTPMIPAGGQAFVNYAYTGT
VDNEDKIDPSSSAANGINIGGRDGETQSLDRTREGYIEVIEMGEIYGNDDILEALTHVDGVPDDCSKLQGL
KYSDLGTKLYIERPTGGLTGSASLVAPGSGTDFSYDPVAIDNFAWDDIWHQAGSIEPDLRHGRDRSLVFYN
QQVVDTSWNGTGEYGNGTADKDSVNAVNALLMHRAVINEFVLDNVTLSGTDWVVTMPTKRYSVPIQD
PNNSNHDGRHALPPFTSTFWENGACEPVDIEYYDREERTKVVGGFSPPPPGVNNALCWEANVITFNDS
NILSSAHSVNISIAEGFQNGWVRMHFDDEGHQQVGHSNQEGYHTYYGLPVIGFMVQDFVNLNAAPGV
MATYGGSFKHKYETCITGDVNQRYESCHYAY

>WP_239795478.1 hypothetical protein [Candidatus Nitrotoga arctica]
MQKFKRKSLYLALVAAVSSVGIANTASSAVHVNSNGLGQVLIYPYYTSRAGMDTYLSVVNTTSSAKAVKVR
FTEGRNSREVLDFNLYLSKNDMWTGAVVNTANGAKLVTADKSCTAPAIPAGGKEFVNFAYSGANLEGIVG
SGGDGETTSLDRTREGYFEIIEMGTITNTAIEAAVTHVSGVPANCAVVQTATMDMGVSSTVIVGGQSARA
NPSTGGLAGTASLVNVAGGTDFGYDPVALDAFAPTNVQNIWNPPGSILPDMRFADHTGVVFNGGNTVT
SSWSSGEDTVSSLLMHNNIINEYVLDAATLSGTDWVITMPTKRYYVPVHNPALSTDATQLFSPFTHKFWL
GGACEPVSISYWNREEGNVLIQDFSPPQAIGGTSLCWESTVMTFNNSAVLASANGVNVPVNFDNGWLR
MSFSATNVLVFNGQDDANGDHHNDASHSMTSLDDDTYFGLPTVGFMVQDFINQNAAPGILATYGGNF
NHKYTVSISEAN
>WP_171164597.1 hypothetical protein [Usitatibacter palustris]
MNTFKRKALTSAVLAGLGAAGTAQAVYLDPNGQGQALIYPYYTVQASNGNGYNTYISVVNTTTAVKVVK
VRFREGKNSREVLDFNLYLSPNDVFAGAVVPTSDAATAGGRFVTTDTSCTNPALPDLGGGLRGIDFRNYLY
SGANAEAAGVNEGLDRSREGYAEIIEMGTLSPTVGPGSAAVHGASGSPACGTPLTGQTVPGVAAAITTPS
GGLNGTGTIINVNSGADAGYNADALSNLTATPIYFDIGNDTPNFSNADPVSVVVANNRAYFSTWAATPVG
RAGAVSATMMRSSVINEYILDSGTKSQTDWVLTFPTKHHFVTTATATTPFTNKYVAASGACETISFSYFNRE
ERGATAAGADFSPLPPGAAANTVCWESTVVSIRNGATHMPTGTASGVLGSTNAVPINVTTGFQNGWAS
LTFTGTNAVAGAGTGLASATGVTDNILTGATSAVAQTFQGLPVTGFMVRNLNNSTLTCGTATCQGNYGSLF
GHKYLVTAAPTP
>WP_198970414.1 cell surface protein [Xylophilus sp. ASV27]
MKKSLLSLSIGAFIGGLTLAGAANAAVSIAGTGTGTGGNKLTTTTATVLELNPGGIGHIGIIPYFSTQNGNHT
LISITNTDATNGKAVKVRFRSAANSDDVFDFTLFLSPGDVWTADVGKDAATGLSQLYTADNSCTLPASVNG
KFITDRLPPTFTDAQKAEWTREGYIEVLNMADVPPQLSDGTTTNPLFTATKHINGVAPCTGSTLTALATDN
VTTDGAKLNALGLDTPSGGLYTNAFIINSANAYMAWSTEPTAVEARASAGGAPGRGNLVFSPQTGNKLGS
GEVTNIGNLTADPLLVSGAPVPYVSGIVPLQFDFPDLSTLYIGASTTTTTAASQAAALSKALAVSTVKNDYST
LASIGSKTDFTFSMPTRRYAAAVDYSASPAKVVFNGDNPTYFTAANTTVKGNLACVQPGGSVTGWSRSE
QYLTSGPVISPGTATTLQFCGEVSVLTVNNNPTNPAGNGVLGATVAVQNLYTGYQEGWINIGTPGLSNAG
LPVLGSAYTSAVGAPAAGMSTNYSWTYAHKFTKAGAQ
>WP_146228730.1 cell surface protein [Xylophilus ampelinus]
MKKSLLSLSIGAAVAGLTLAGAANAAVSQQGTGAAAGTTAAGTLHATTTGATLELNAGGIGHIGLIPYFST
QAGNQTLFSITNTDTVNGKAVKVRFRSAANSDDIFDFTLFLSPGDVWTANISRDVATGGSRLVTNDNSCT
LPATAVLNRGFVTDRLPASFTAAQKAEWTSEGYVEVLTMADIPPVVYTDGAARPNAAGTLNNSTNALYTTIK
HVNGVAPCDATQLATVLTDKPTEADLVAAGFDTPTSGLYTNAFIINTANALMSWSNESTAVQAVTVAGGV
TTAARGRVVFAPQTNDAVTQTINQLTADPLLAGGLSSTGVAVPAPIQALQFDFPDLSTPYVDVNALSATGA
GSPAAQSSRLSGSLAATSIKNDYSTLAAIGSKTDFTFSLPTRRYHAAVNYATTPFTPVYTTPAAGFASYFTAA
NTSLLGSLLCVNPGSAPLSVFDRSERTLTTSNGAVVSPGAATAGLRFCGEVSVLTINNDPSAPAGKGVLGAT
VAVQNVFTGFQEGWINIATPGIANAGLPALGSAYTSAVGSPANGVSTNYSWAFKHKATFP
>WP_156362758.1 cell surface protein [Xylophilus sp. Leaf220]
MKKNLLSLSIGAAVAGLTLAGAANAAVSISGTGPLSANTFTATTAGATLELNPGGIGHIGVIPYFSTQNGNQ
TLFSITNSDTVNGKAVKVRFRSAANSDDIFDFTLFLSPGDIWTANISRDPTTGTSRLVTTDNSCTLPGIAALN
RGFVDARLPAGFTAAQKAEWTREGYVEVLTMADIPPTAGTAAAPDLYYATKHVGGVAPCTDSVLSKLTVP
ANFADEASARALGFDTPSTGLYTNAFIINTANAFLSWSNESTAVQAVTAATGVAARGRFVFAAQTNDAVQ
TTAANLLTADPLLVGGFSTNGGVVAATVPPLEFDFPDLSTPYTGTAPLATTGTGSPAAQATRLSGSLATTSVK
NDYSTLAAIGSKTDFTFSLPTRRYHAVVSYVGAPNGIGGGAPVAGPIAVYSTAPTFTGRTFFIASNTSLQGN
LLCVNTGSAAPVTAFDRSERTVTTGNGAVVSPGTGTAPLSFCGEVSVLTINGNASNPAGNGVLGAAVAAK
DVFTSYQEGWLTIATPGPFVDAGNAGGLPVLGSAYTSAIGSPANGVSTNYSWAFKHKTTPSLVANP
>WP_143890119.1 hypothetical protein [Tepidimonas alkaliphilus]

MKKSSIALGVAALMAAGVHAAIVQGNATQIVVNPNGIGHKLIFPYYTVQGNNATLINIVNHDQLNGKAV
KVRFRGAANSDDVYDFTLLMSPGDVWVAAVTQGADGRAQLFTPDSSCTLPANVNGSFITGRLDPNSTV
SLEEQTREGYVEIINMADIPKNTASTSLWTAIKHGSNGKPTNCNAAVVTQLSGDAVANINSVDGSGQMTS
YGLSFPSTGLSGDWIIINQQTTAAWSGSATALEARNGGAATTANFVFFPQSGSGVSSTTASNWSADPLFE
KGVVGAAYYDFPDLSTAYLTSTALSVSNLNSYAAAASYRNLVAAALAKTVVANEFVTDSTIDAKTDLVFSQP
VRRYYAAVNYGSTATAVYATSSGVYTSNNTALSNRVLCVRRPTGSSLLSFTAYDREEATLSPSGAVISPGTPTR
FDICGEVAVTSINGQGGAADPSALNGKLARNDINLGSLIDGWIEFNTALDTGDRVPGVTGLPIIGNAHLRA
RNGSVNYGFTWPHKYNYSNK
>WP_052231333.1 hypothetical protein [Tepidimonas taiwanensis]
MKKSSIALGVAALMAGAAQAGIVQGSASTIAVNPNGIGHKLVIPYFTTQGNNATLINIVNTDRTNGKAVK
VRFRGAANSDDLYDFQVLLSPGDVWTAALTQGADGRTQLTTSDNSCTRPASVNGSFLTGRLDPNSTRSLE
EQTREGYVEIINMADIPVNTASGSLYMSIKHGSNGKPGNCNAAAVTALDDDNLGQASATASYTALGLDFP
STGLTADWIIINQATTAAWSGSATAFEARSGGTPAAATTANIVYFPQSGSGVSTTTAGLYSSDPLFINGDVQ
AAYYDFPDLSTPYINGVTSAGVYRNTLASTIARSSVINEFVTSDAIAAQTDLVFSQPLRRYYAAVTYGATAGV
VRVNSVGAVVSTGEVYTSNNTALINRVLCVRRPTGSQLLAFAAFDREEQTLTPSGAVISPGTPTQFNICGEV
AVTSINAGDNTQPSALSATLARNNIELPFADGWIRFNTTLDPVDQAGTAPNVVTGLPIIGNAHLRAANGA
VNYGFTWPHKYNN
>WP_068606511.1 hypothetical protein [Tepidimonas fonticaldi]
MKKSYIALAIGALAAGAAQAGVVTTSTGAATLEVNPNGIGHKLVVPYYTVQNNNATLLNIVNHDQTNGK
AVKIRFRGAANSDDVFDFTLLLSPGDVWTAAITADADGTAKLTTADNSCTLPAKADINRKFVTTRLDPYSSK
SVAEQTREGYVEIINMADITKNTATTSLWYAVKHNSSGVPNSCGSSVVTALLDSSLIESSTLSDYLARGLENP
STGLSADWIIINQTTTAAWSGQATALEARDAAGGRAEKANLVFFPQIETEFGVSATTAGQWTADPLLAGG
TVKAQPFDFPDLSTPYSAAGATTANELLVSTRTLAKTSVANEFVTSAEIAGATDFVFSQPLRRYYVAVNYMG
GTDGKTATQVPLSTSVGVPYGPGLTTVSDRVVCLVRSGSQPTFLSYTAFNREEATLSGSTSAVVSPGGPGQ
TFRLCGEVAVTSINAGSNTAPSALGGTLARNNIELPYADGWFRLDTDSNGSTSGGGLPIIGNAHLRAANGP
VNYGFTWGHKYNY
>WP_155294660.1 hypothetical protein [Pseudomonas mendocina]
MKTLAKAIALAATMGAAASASAAISLNHNGQGEVLLYPMYTVENGNDTAISVTNTTNEYKVVKVRFREA
LNSQDVLDFHLFLSPKDVWNGVVVATDDGAKLITRDTSCTVPTIPADGQAFTNLAYTGGAVETALRGEDG
GPQDLKRTRVGYAEIIELGVLDPTVATTSGTVNGQPATFGTAIKHVNGVPGNCGAIVSEYVSGQWKDVAP
NDFSYGFKDVAAINASTDGGLGGLYGVGTVTNVFAGQQIGYDATAIDGFISLVDAETAGGAAIGNLHNAT
GYSFPDLNGNRTPGAAVTAPAAGLGGSLVANIGGTNYTFGKTIDAVSAVLQRTSLSNEFSVENAVGGGTD
WVVTFPTKHHYVWDSQIQADGTRPITTAGVPNGNAPGVDGTNVADYLAGTAQGNVSPFQTASLWQG
VDGAARSQVPVTLGYLDREEKEVTVVRDISFSPRPTIPNVSFALSYEANVVTFNNSNVLVAGDSYARYNWA
LETGYTSGWATIGLDTNAANVVTATAPAQTFRGLPALGFATIKVTNGDLGGVLSNYNAAWNHKSN
>WP_008296404.1 hypothetical protein [Congregibacter litoralis]
MIKKFLKPLGIATAVAAASAGYVNVATAQPAVANNALGDLALVPYYTVNGEWITGIHIVNTSDRTQVVKFR
FRRAPDSLDALDFNVIMSPQDVYAGFLSDDENGNIVWSANDTTCTAPAATDGSLQMPAIYREGAETGYV
EIIGMGAPIDEDQGIAVAAKHTTDTLVPADCAAVRSNFFANGVAGTTRGIVDNANSVQSASDVLPAPYDA
VNDNEFELTENVLKVSYFIRDNATGVEFGDNAVHIADFLPEPSMTNQQFGYLSGDLNGFDFPDLDGGEP
VNGTRNLFEGLRAGDVLGVTNLINEWTANPANGAALSWVVTLPGQYLMLDMPAYIASLDDEDVDCDTG
LVADGNVTAGGDVCDFRDIPVNATVVPYNREELTEIPESGDLTVSPALPGQPSVLQLAKETNVITFGGNAV
LGVSDVDITADLGQNFGWLSLAVTAADPLEQAICAWDGPDTPAGDEGLPAGAALTMTCNAVVETAVPM
VGFAAWVRNVAANPDASYGRAVAHSFTVAP
>MPT01079.1 cell surface protein [Pseudomonas sp.]
MKKNVLALSIAAMVGGLGFAGAASAALSVNESGTGHILMVPYYTAQNGNMTVFHLTNTDTVHGKAVKV
RFRGASNSDDVLDFQVFMSPGDVWTAAVTADANGLSQLVTADNTCTLPRISAGTPVKFVTDRLAKTDW

TASDKAAQTREGYVEILNMADIPDNNGDKGLFKAIKHVNGVAPCTSGILNATLSLTGVGPGATNLNLPNN
TAADLTVPTGGLTGSWYVMNVAQTTTFSGATPAIVASGATRNVFSHQKTGTALLETADPLMVGGAIPAQH
YDVPDLSTPYEVAATTAAAQAEELTSAIARTSVINQYATDASISAKTDWVFSMPTRRYTIAANYAAGAVNQ
VVGNVTGTSLAGGTVTLPGSTPAYRFINNNVAVANNIFRTAGNTTVNSAGQICVEAQSQTFFDREERFQE
DGAIFSPGTISRTQLCGEVSVLAFSADNSALAASVARQNVAAPYTNGWGVVNFNAPTPLLGAAFLKLTNP
EAAPGTSGTYGITWPHAYRASAQ
>MBU0789994.1 surface layer protein NpdA [Gammaproteobacteria bacterium]
MRAAALGAALCAFHLAAPTATAGVISGTGAAPSRTTLGASNAVAFRVSETAPGHHLMFPYFTVQRGQMT
VLHLINTDFSNGKAVKLRFRGAGNGDSLLSLQVLMAPGDTWTAALTTGPDGRAQLATADNTCTYPALLKG
TAQPFLADRLNPAWNQETANNNTREGTVEALLMADIPSAKLYGAQADAHSNLYLATRLLTGQAPCTLSSL
DDALNNDISDETQASGLGFATPSGGLSATWYIIDVPGSTTLSGPATSVVAVDNAGASARGNFVVFPQTTLV
VDRPERYTADPLLVSSGLASRSKTVEGVTASPTASQVVQAQFHDLPDLSTPYYLSPSDTHARQTAADLTGRL
LTNTVRNQYARDASISAQTDWILSMPTKRYSVGYDYSQEATTALVYSVVPPVGGGQVQFYDSTSTLARSH
QACESYVGTAADREGVWTTGGPILIAPPPNVASCGAAAIVSFNAGASVLSSSVSRWDYGRGFTNGWMS
LAPEMRPGRPVIGAAVLKLTNPNATPGVAANYGITFPHILSRQP
>MBZ0134507.1 hypothetical protein [Rhodanobacter sp.]
MRLKRVTRGVAALVGTLACIGTAGAGVSFSNIQATQVPSTGSGLAGAYLPVADTLAVSPHHIGHLLVVPYF
STQDGNVSLLNVVNTDTVNGKVLRVRYRGAVNSDALFSFSLYLAPGDVWAGEVSQSGGQSRLQTNDAS
CTLPASINLPFSTDRVQGKGAQTLEGYVEIINMADIPPTEASATGNGALIASSNSLFKAVSHVSGTPDCASIA
GTEALNPTNPGAPFPNSWGDRGYNFPTGGLMAHWSIINLTKAGSFTGTATAIAARKSGSTGDRAVANLV
YSAPTDSPQYDGDDAAALAAGRHPSQLSADPLLAGGILADGSAAPPALTALNLDFPDLSTPYVTPASNLGN
AIRQAETISDLLATKAVINEYLTSPAVSFATDWTLSQPTRRYNVALDDKTNQPVFAKALDYTQGMPPRVPA
SFFAPAGTYYTPGNVRVDANGLLCVNASGAYAWDAAQRNNGPLFSPVPPNQRIDLCGEVSVVTFNNSQ
ALGAQLTLQNFVPKNASSEVFTDGWVRLNLIGKGLPIIGHAFAKALGSSSNLGGTWAHGTDKSGL
>MCG6966808.1 hypothetical protein [Chromatiaceae bacterium]
MKKRLLALGVATALGMSAGVASAMQTEPSGIGMYNILPYYSVQSGNNTLIQITNTDEVNGKAVKVRFRG
AEWSDDVFDFTLFLSPGDVWTGAVTVNGLVAHMDTVDNSCTLPANINQDFVTFRAALSEDSNAATREG
YVEVITMADIPPTVGGDPEEDLYDTILHHNGEAACDSGVLTALTEDSANVAADQGMENPTPALTSYAIVIN
VPKSKAFAQAAIAVNPDSVNNKIYYMQKNVEINPADLAELQALTADRIFAAQGAPDNGVGLQMYQFDLP
DLTTPVEGGTAIAQRDSLTDALAQNGDAVVEYVTDSAIDATTDVVFTQPTRRFFYNYIENADGAYELNGW
TFDIEGDAGSVYDSLDGVSNRIAVGNPTFYDREEGKLVGEDDIVISPTPPNVALTFSLKGEASVISINNGAIP
TGSLAASLTANNYETNLEAPDGWAILSTTTTSGTVGPLPIVGFTAINILNNAVGAAGTNYGMVLPLRQREY
VAP
>WP_029134493.1 hypothetical protein [Sedimenticola selenatireducens]
MNKKLLVASVAGVLGGMVLNAANAATVVSPSGIGQTVLVPYFTAQGGNATLINIVNTDSTNAKIVKVRFR
GAEFSDDVFDFTLFLSPNDVFTGAVSQNAGGGATFSTSDKSCTLPASVSQDFVTSRVASGAAGTLEGYVEI
FIMADLVNGAAPPPAVDIWDKVLHSAGVAPCGITNADITALGLDPSVGWPGDAPGPYDTAVAAAAPLGI
AAPTPTLMANSFIVDGPRAAGYGVEATALVEALGGPYHVTFSPQLATQVPAGRWDDWSSDQGLADTPA
AVGGDVARAAYYDLPDLSTQANAVVNGAPVAANHGTTNTAIADVSAAIASRYVANEFLTDSTVAAATDW
VFSMPTRRYYMQYVPAVAPATGFVFDGAGAVPIGPYFTGVTAANEQPLASFDVYDREEQTSAPASFVISP
GVPGVLNLTGEVGVIGFNGSATTSEVLSAKLTLQNVDTAGYADGWMAIDITGGVPVEPVIGFAAIRASNN
PAGGDSFNYGAAINHRFY
>WP_134356545.1 hypothetical protein [Nitrosococcus wardiae]
MKKTKLATAVSLALAGSAGALNTAQAVNINPDGLGEVLLYPYYTVRDDHDTLITVTNTTPDVKAVKVRFLD
GQNTKEVLDFNLYLSPFDVWTGALVATEEGTRLTTPDTSCTVPTIPEAGVEFRNFEFGDDEPEGVDTSLNR
TREGHLEIIEMGVVVDEGPDADFTPATAATHVNGQPADCAALEQSWANGVWSTSPNANVELNPGGLFG
HGLVINVSRGTAHGYKATAVDNFFFFEPFHTNPGSLLPGVESADISSDVFVSQQGATNVPTVVQSTWNRG

LDSTSSVLMHDSVMNHFVTDESIGAATDWVITFPTKRFHIAGDVATPPFTETFTVNGACEPVGLAIWDRE
EQTTGGGIDFSPQPAGGVNALCWETNVITFNNGSVLGSELELNVDTSSVGEDGWMRLTFNDEDHRLLS
NEGNEFFGLPVIGFAVQEYVNGVDQEGVLINYGGMFDHSFSRQISGSGAELQ
>WP_164210386.1 hypothetical protein [Wenzhouxiangella limi]
MKRNTLTTAVLAGLTGIAGMASVANAVNVNPDGLGQVLLFPYYSARGGNDTLISIVNTAERGKAVKVRFIE
ALNSREVLDFNLYMSPFDVWTASITATDGGGAKMVTRDTSCTVPYFVGDSEDGVGEQEFLDFEYTGART
NFDGGPQGIERTASGYIEVIEMAEMAVGEGGSYDPNEDGITPGIVEWASKHVNGVPRACSLLERFWTQN
DPVALWLQSANFGFTSGTGEEATFNGATGGLYGSGSIINVADGTMFSYNATAIDGFWAAGTGSHTNPGS
VLPGLNSGTNAESFVFDNGALENNQWDSTTPVLALNAALTLDQVLNEYVTDADINARTEWVMTFPTRR
FHVDALGKGLIDPGDDAIPPFTRTWFYTVDDGAVDRRFFACEDMSISFWDREEQTEGIDVGPPIVSPPPP
EQAPDLFQLCAEANVIRFASDDSLPDATEILGEPLRESPFRSLGYTNFELPFDSGWARFELGAIPSNSGVFED
NDGVRRSVSSTDGRQVFGLPVVGFGVTTYTNGDAGAGVLANYGGTYQHRGTRAFVDSGADRPE
>WP_197702487.1 hypothetical protein [Candidatus Nitrosoglobus terrae]
MRKTKLAMAVSLALAGTSGTFVANAQVPNAGINVNPDGLGQVLLFPYYTVRNNNNTLIAIGNSHPNEVK
AVKIRFHEGKNSKDTLDFHIYLSPNDMWTGSLVQSQNGVMLTTADKSCTVPPIPANGVEFRNTVYAIQQP
DGEDMTLDRTREGYIEIIEMGVVTSAAGPITSTNTNVTNLAQAATHVNGVPQDCAALNNAWGSGGLW
ANTNPQGSTGVTSPTGGLFGDLAIVNVPRGTDYTVAATALQNFALGTPGQLVGTLGNEDLHAAPGSDFP
NLGFDAGATSGAPVPILPADVMTFSNVFVDGGMVSSQWGNRIDAVSAVLMHASVINEYTTEPGLNANT
DWVLTFPTKNFYVCAVNPAPTANSPTPNTGNSDCAPGVVGSGVLAGATFGPPFSLPQGTGFWTGGAPQ
NVNMNIFDREETTTVQPIDFSPPSPTGANTLPWEVNTITFNQPSGMTTSTVLGSALVSNVSTGSVGVNG
WMNLDLTPGHSMSTNGLVVNNAGGGAATTTYIGLPVIGFGVEEYVNGSINGVLANFASVFSHRLCAATT
SGGEGNTDCGGNITLPPPATGTGTSTSTSTSTDTGTGTGTGTGTGTGTGTGTGTGTGTDTGTGTDT
GTGTDTGTGTDTGTGTDTGTGTDTGTGTDTGTGTGP
>WP_013034518.1 hypothetical protein [Nitrosococcus halophilus]
MKKTKLATAVSLALAGSAGVLNTAHAVNVNPDGLGEVLLYPYYTVRNGQNTLISLVNTRDEVKAVKVRFLE
GKNTREVLDFNLYLSPFDVWTGAVVRTDAGARLITNDNSCTVPTISEDGEDFRNFTYVGNQADGEDTSL
NRTREGHLEIIEMGVVTDTNPGDPTAFNPATAATHVSDVPADCSVLEQAWSGGVWATNPSTNIVSSPGG
LFGHGLIINVAQGAAYSYQATALDNFFIVPFHTDPGTLIPSLEDADTLSDVFISQQGTPAVVNSLWSRGLDS
ASSVLMHNSVMNHFVTDPDINAATDWVITFPTKRFHISVKDLDTGSAPAPTPPFTENFMEGGACEPVGL
AIWNREERPQTGGLDFSPQPPGGNALCWETNVISFNDKDVLGSELGLNVDTSSVGNDGWMRLTFTDP
DHQLVSEEGNVFIGLPVIGFAVQEYVNGVSNEVLTNYGGLFEHAFSRQISGVQTQ
>WP_036499593.1 hypothetical protein [Nitrosococcus oceani]
MRKTKLATAVSLAIAGSAGMLNTAQAVNVNPDGLGEVLLYPYYSVRNDQDTLVTVTNTTPDVKAVKVRFL
DGQNTKEVLDFNLYLSPHDVWTGVVTATENGARLTTPDSSCTVPTIPEEGVEFRNFEFDEPAEGVDTSLDR
TREGHLEIIEMGVVTDSADFAPATAATHNADGVPNDCSVLIQAWTSGVWTSNRNANIELNPGGLFGHGL
VMNVSQGAAYAYQATALDNFFPFFGLGDTFNFPSHEEPGSLLPGLESAALNSDVFVSQQGATNVPAVVSS
TWDREIDSTSSVLMHNAVMNYFVTNESIGAGTDWVITFPTKRFHIQAEIPTQPFTETFTADGACESVGLAI
WNREERAQTGGLDFSPQPPGGNALCWETNVITFNNSSVLGSALELNVDTSSVGPDGWMRLTFTDEDH
VLESEEGNVFSGLPVIGFATQEYVNGVDQEGVLINYGGMFNHAFSRQVSGGPL
>MCB1621121.1 hypothetical protein [Thiothrix sp.]
MFKQKKLALGIAAGILGLAGIMTSAQAVHVNPDGTGQVLIFPYYNAHSGYVTNINLVNSTDETKAVKIRFR
EGKNSNDVLDFNIYMSPQDVWTGSVAAATDNSGELVGSVSTRDRTCTLPQLASCTEDQCAVTIPFTGQK
VYTGIEAADTREGYVEVIEMGVVTDKTVQDGVLHHNGVPYDCSAVEEAWSKGTFTQGKGAQAAGMSA
PTGGLFGSSAVINVPKGSAFAIDPVAIDNYSTQAQHYLSDDPDNFLLPSLASGDITTSSILAKDANGDAELVE
TEWNTVPDTCLNDADPLTPQCGVNPYPIAHVLLASNIMNEYFLDPTFGYDGHTDWIITFPMKKHGIHKT
TDVALTMDGVYDREEGQPNVVIDKSFGFSPVLPGMEKEQSALTREVNVLTFTSSDPSYDASNTVMSSHT

DVKLSVGSFVHGWARMDFPGYNLADGWSDSVTYGKTGTSYGSSSAIYEGVPVIGFSAIEGNVSENPNAR
FGDALPHKIKR
>WP_028488679.1 hypothetical protein [Thiothrix lacustris]
MFKKNKVAMSVVTGILGVVGVISSAQAVHVNPDGTGQVLLFPYFNANDGYVTNVNLVNSTDQTKAVKI
RFREGRNSNDVLDFNIYMSPEDIWTGSIKPTKDSSGNLVGTLSTGDRTCTLPALASCDDGKCVAEQAFTG
QNVYKDVTAADTREGYIEVIEMGVVDEPTVKAGVLHSNGKPSNCKAVEDAWNKKTFMQGEGAAAKGL
SQPTGGLFGSSAIINIKRGSAFAVDPIAIDNYSTQAQHYLSDDSNTFLLPSLASGNVTSSSVMTKNSSGGSE
LVVTEWSKQADACLEDKDELTPQCGTNPYPMAHALLAPHLMNEYFLDPTDGYDGHTDWVVTLPMKKH
GINQATKDVVANFENGIFDREESRASWAAKTEFGFSPVLTGSKAESSLLGREVNIVSFKTTDPSFDNTCDVL
SSEARQLVSTGAFVSGWARLSFPGYSLSSGFKSATGYGLNAATYTATSNVYKGVPAIGAAFIEGNVSNNPN
ARFGDALPHKIQRD
>WP_151702573.1 hypothetical protein [Nitrincola alkalilacustris]
MKKKLLPLAVLAGLAGGMSTAQAVNVNPDGLGEVLLYPFYSVEGGNDTYINVVNTTNQVKAVKVRILEA
MNSQEVLDFNLYLSPNDHWSAVILDNPNGSGAILRTADTSCTAPAIPANGVAFREFEYAADSDNSIARTRE
GYVEIIEMGVLDAAVVPAQHAGAIHTGAGVPANCGAIISAWQAGGVWANAANTGINAPTGGLYGYGVLI
NVAEGTNATYDATALDAFAQGIALHTNTGSLLPSLNSAAPTSSVFDNGATVTSAWAGTAPAAGVNAVSSV
LMHDNIANDYVLEPTIGAGTDWVVTFPTKRFYVNGAGAPFAPFTNAWNPANSTACEVIGINYFDREERG
QTPSDIDFSPLPPGTPALSLCYEANVLTFNNSNVLNGSARVNRNLEIDGFNNGWLNINFQGTFNGIARTLT
ATSGEVYQGLPVIGFAVQKYVNGTLPGGVLSNYAGLVNHKATRLIN
>WP_036525993.1 hypothetical protein [Nitrosococcus oceani]
MRKTKLATAVSLAIAGSAGMLNTAQAVNVNPDGLGEVLLYPYYSVRNNQDTLVTVTNTTADVKAVKVRFL
DGQNTKEVLDFNLYLSPFDVWTGVVTATENGARLTTPDNSCTVPAIPEDGVEFRNFEFDEPAEGVDTSLD
RTREGHLEIIEMGVVTDSPSFAPAMAATHNGDGVPNDCSVLEQAWSGGGVWRGDGNVNIELNPGGLF
GHGLVMNVSQGAAYAYQATALDNFFFFDPDFGFPFHTDPGTLLPGLQTADTVSTVFVSQQGATNVPTVV
NSTWVNGLDATSSVLMHNAVMNYFVTNESIGAGTDWVITFPTKRFHIQTAIPTPPFTETFTADGACEPVG
LAIWNREERAQTGGLDFSPQPPGGNALCWETNVITFNNSSVLGSALELNVDTSSVGPDGWMRLSFVNSI
DDDHQLASLEGNTFFGLPAIGFATQEYVNGVDQAGVLINYGGMFDHAFSRQISGSGT
>WP_152809220.1 hypothetical protein [Ostreibacterium oceani]
MNKKLISAAVLLGMSGAATAVHVNPDGKGQVLLYPYYTVNGGKDTYFHVVNTTNEYKAVKVRINEGVNT
WEALDFNLYLSPYDVWAGGLTMGEDGVPELYTPDTSCTAPAINGRVDLRTFNIDSYAQDQEPAAFDDIDP
VERLAEGHIEMIEMGIIEDDADPATAALIKTYIKHVNGVPGDCEALVDLFRSGPWATNEAQFLHAPTGGLF
GTAEIIDVANGIDRGYSATAIADFWDLDAATDATNAHTFPGTSYPNLNGQTVQAGTLLVMGSVDADIAYT
DAAGAQAVVNDAGLGSTVEALSAALSVASLSNQFYVADSVNGATDWVVTFPTKHFFVNGDSTAADFKS
GQFGLDPTPGPLEADVFNGLAPFDNGFAVDIEMSLYDREEETVTADIDFSPSGTNTGRMLFEVNVLEFAA
DRSVLDSTLASQINVPAGWQAGWAQLDFSAQQVTGATLAGMPAIGFAAQAAQNGTLGGGSTLANYAA
LFDHVYGKALP
>WP_207251913.1 hypothetical protein [Thiothrix fructosivorans]
MFKKNKVAISVVTGILGLAGVMTSAQAVHVNQDGTGQVLLFPYYNANDGYVTNVNLVNSTDQTKAVKI
RFREGKSSNDVLDFNIYMSPEDIWTGSVKAGDDGKGNMVGTLSTSDRTCTLPALASCADGKCQAEVPFT
GHNLYKGVTAADTREGYIEVIEMGVVEDTAVKAGVLHKNGAPTDCKTIEAAWTPSTSGGKGTFSQGAGT
AAAGVSAPTGGLFGSSAIVNVGKGSAYALDPVAIDNYTTQAQHYLSHDPDNFLLPSLASGNVASSSVMTK
TSTGENEMVVTQWTAQKDACLEDKDELTPQCGTNPYPIAHVLLAPHLMNEYFLDPTDGYDGHTDWVV
TFPMKKHGINAATTDVVATFENGIFDREEGRAGWVASSGTSFGFSPPIGMGNVKVDDSSLLNREVNVISF
KSSDPSYDASRTLLSSDSKQSVSVGAFVSGWARLSFPGYSLSSGFQSAQGYSAKPSTYTADSNVYKGVPAT
GAAFLEGNVSANPNARFGDALPYKVQRD
>OQW99296.1 hypothetical protein BWK73_50720 [Thiothrix lacustris]

MFKKNKVAISVVTGILGLAGVMTSAQAVHVNQDGTGQVLLFPYYNANDGYVTNVNLVNSTDQTKAVKI
RFREGKSSNDVLDFNIYMSPEDIWTGSVKAGDDGKGNMVGTLSTSDRTCTLPALASCADGKCQAEVPFT
GHNLYKGVTAADTREGYIEVIEMGVVEDTAVKAGVLHKNGAPADCKTVEAAWTPSTSGGKGTFSQGAGT
AAAGVSAPTGGLFGSSAIVNVGKGSAYALDPVAIDNYTTQAQHYLSHDPDNFLLPSLASGNVASSSVMTK
TSTGESEMVVTQWTAQKDACLEDKDELTPQCGTNPYPIAHVLLAPHLMNEYFLDPTDGYDGHTDWVVT
FPMKKHGINAATTDVVATFENGIFDREEGRAGWVASSGTSFGFSPPIGMGNVKVDDSSLLNREVNVISFK
STDPSYDASRTLLSSDSKQSVSVGSFVSGWARLSFPGYSLSSGFQSAQGYSAKPSTYTADSNVYKGVPATG
AAFLEGNVSANPNARFGDALPYKVQRD
>WP_152808267.1 hypothetical protein [Ostreibacterium oceani]
MKKSLFALMAAAAFSAQSVEINPDGTGQVLLYPFYTVKSGFDTSINITNTTNTTKAVKVRFSEGKNTWEVL
DFNLYLSPYDVWTAVLTKNQAGGVRLVTRDTSCTVPAIPAEGILFRNALYAGNDAEGNGEAEADQSLTRLE
EGHLEVIEMGLVQNSASHPLATAIKHVNGVPNDCNAVTAAWDGGLWSADRNANMSAPTGGLYGSTQFI
NIPQGISTTVDATAIDNFWELSGNGNSANNRPGTSFPDLNGQVEEGLSLLKLGNKSSSVVANGGLVTSTW
NETIDAVSAVLMARSISNDYYVNAGLNGATDWVISFPTKHFYVNGGESFNGLGPNRPPFKSSFDADNDE
GACETVSLSYFDREEERNQVNTEVDFSPSVNPDTGPAICWETSRITFNNRSVFGASATLNNIEIPFSEGWA
EIGFNGRLVSNESHRYIGLPVIGFSAVSLQNGQLNGGSTLANYATSTPHKRTRQIN
>WP_013221243.1 hypothetical protein [Nitrosococcus watsonii]
MRKTKLATAVSMALAGSAGMLNTAQAVNVNPDGLGEVLLYPYYSVRNNQDTLIAVTNTTADVKAVKVRF
LDGKNTQEVLDFNLYLSPHDIWTGIVTATENGARLTTQDTSCTVPKIPEDGVEFRNFKFGQGEPEGIDVSL
DRTREGHLEVIEMGVVTDSSGFAPATAATHVNGEPADCSVLEQAWNGGDWAEDKDRNIVSNPGGLFG
HGLVMNVSQGAAYAYQATALDNFFFFEPGSPFNFPSHTNPGDTLPSLESAHPRSTVFVSQQGAANIPTVV
DSTWPFVDRRIDAASSVLMHNTAMNYFVTNESIGAGTDWVVTFPTKRFHHTVVDAPPLPPFIETLTPDG
ACEEVGLTIWDRDERTQQGGLDFSPKPPGGVNALCWETNVITFNNTSVLGSALELNVDTSSVGPDGWM
RLSFVNNINDAHQLVSEEGNVFYGLPVIGFATQEYVNGVDQAGVLINYGGMFDHAFSRLVSGSGAQ
>WP_254264629.1 hypothetical protein [Gilvimarinus sp. DA14]
MKKKLLPLAMLAGLAGAAGTAQAAYLNSDGLGQVLIFPYYTVNGENFTNINLVNTTGDAKAVKVRFLEGE
NSQEVLDFNLYLSPYDHWSGAVALSENADDGSLPWSTGDGEMPAKIITQDTSCTAPEISEDGEDFRNFAY
AVDNSGAQPVLSDDTNKSLSRTREGYVEVIEMGVLSDVADSDPADDTDDSFAVATAVTHVDGVPANCDL
VRNAWNGGVWENDPSLGVDAPAGGLYGYASIIDVAEGTNASYDAVAIDDFIDTQLNQATGSLLPSLAQAL
PISDVFYYDETAMAGSVATNNFTAGTADTVSSLFMRDTLSNDYVLDEVLNAETDWVITMPTKRFYVNGN
TVTNDNGTPGDTSDDFDEPVALQPFNAPWDGSVACEHIDIEYWDREEAYPVAPVIPGEIDFSPLPPQITET
PDGFQFCTEVSIMTFNGGSVLDSSENLTYGLNLEEGYEHGWAQIDLSNPYFPGESGTDREAREIAGDATTF
TGLPVVGFAVQKFENGTLEGGVLSNYAGVVKHKYTRMITTSAP
>WP_136680225.1 hypothetical protein [Neptunomonas sp. XY-337]
MTKKLLPLALAAATGLAATAAQAVYLNTDGEGQVLIYPYFTVEGGQNTLINVVNTTGETKAVKVRILEAM
NSQEVLDFNLYLSPEDHWSAVISANDNGGASILSNDTSCTVPAAISEGTAIEFRNFEYRSDQQNTGVDRTR
EGYVEIIEMGEVSDTDLEAAIEHQNGVPANCGEVVAAWQGSGPFTAGPTAAMNPPAGGLYGYGVLIDPA
EGTDATYDAVALEAFSALPVHTDPGSLDPSLDGAVPPQAEIANGNTYVTASYTDGIDAVSAVLMSRSIAND
YVLEPTIGAGTDWVVTFPTKRDYVAVDPATAPFTAPWDAENTQSCEEISITYYDREEGSQQPNALDFSPQP
PAAAPLSLCYEANVISFNNSDVLQASDRVAKNLALDSGFVNGWMTIDLGAQAGAPAGTRELADTTASPN
TIVLNGLPVVGFAVQQYVNNAVTNGVKANYAGLVSHKVTRELETR
>WP_197744519.1 hypothetical protein [Candidatus Nitrosacidococcus tergens]
MKKTKLATALALATVGAGAWQGAQAVNLNPDGSGDALYFPYYTVRTSDSGSAMTTSIVIVNTTNQTKEV
KVRFREGKNSWEVLDFDLYLSPQDVWTGALTMSPNGGGMLSTSDTSCTVPAIPQGGVEFIPSEFSNQSSS
VYFPDGGGEGLDRTTEGYVEILEMGVINDTSEAATGTNASLSSGQQFTPATWAKHNSAGTPNNCQALVN
AWTNGIWSTTGNVAGVSEPTGGLMGEANIIDVADAVNYGINAVAVDHTFQGVGTANHVPLGTDEPNLA
SASTISNVFTPGASSQVATDTWNGNLKFAKYLSTPTSPVLMNGCPTNGVDAVSATLMRSAIMNQYTIDTS

LNAGTDWVITQPTKWYYVPFQNPSSSGSGGSSGTTIDGDQNGNVNTFCTSAGVSNPSSAPTSAVAPYTV
VFNANGKGTAPEPINATIYGREEQVAIGQTSTIGFSPRPVSQGVSNALPYEVTVLSFNSQSNTSVLGSQLLN
VIQSSGNVQPSGWVNLDLTPGHTMKGTNTFLGLPVIGFAVERFQNGVVQAGVLNNFGAEFVHTYERCV
NGTITNGVCSTGTSGTGTGTGTGTGTGTGGNS
>WP_145955132.1 hypothetical protein [Candidatus Nitrosoglobus terrae]
MMVKIMEVILVAWVLVSTHGIFTLAGAVNLAPDGLGEVLLFPYYTVRNGNMTLLSIINTRQRVKAVKLRFL
EGKNGQPVLDFNLYLSPQDVWTGSLAQSATGTVLTTTDKSCTVPAIPPAGVEFSNTAYAMTSPDGEDTSL
DRTREGYFAAFDMGVVTDSALAAAATHIDGLPQNCAALQQAWTTGVWALTPSMGMGFDPGGLWGSL
QLVNVNQGTDYGASAIALGGFFSTAIHTAASAPLPNLSSANPETDILTQANPAGGVPSVVHSTWPQNSQG
GVDAVSAVLMRRAVINEYTIDPALAARTDWVVNFPTKRFYVPIHNSTNGGIPIVATPPFTQSFWTGGAPE
AVALSLRDREEQVNKGSAAMLPWAVNVMTFRDISVLGSALTLAIDTANMGNNGWMKLKFPVTPNPISS
RQNIHELIDNEGSAYLGLPVMGFAVEAYGNKDAHYRELLPHKFSRDIAMSLDEVIAKTIPPKVAMPKTSAS
KLSMEKAACQTAIEMEIELQTARGSATVNGALAMIACQAAAAIIAASPQVPIGAAAIEAAVKAAAIAAITH
Q
>WP_210230026.1 hypothetical protein [Candidatus Thiothrix anitrata]
MLSKNKIAISVAVGILGVAGVMTSAQAVHVNPDGTGQVLLFPYYNARDGYVTNINIVNSTDQTKAVKIRF
REGKNSQDVLDFNIYMSPEDVWTGSVQPGVGIDGKSKVAAISTSDRSCTLPILVDCKVGECVSKPQPFTGI
GVAPDDTREGYVEVIEMGVVTDKTVADGVLHTNGTPNNCKVLEAAWQYQDKSPLGILDKNDVPVGIFA
KGAGSADAEGISAPTGGLFGSSAIINIGEGSAFAVDPIAIDNYSTAPQHHRADHTEQYLLPSLASGNVTTSS
VMIHNAGGTQLVQTTWDTTAKDTCQDASGIPCGINPYPIAHVLLAPHLMNEYFLDPAFGYDGHTDWVV
SFPMKKHGIHAGAQDITATFENGIFDREEGRPAAVAGGDLDFGFSPPAGGGIDAVDQTSKLLREVNVLSFK
STAQSEAALSRTVMSSLEAQHISVGPFVYGWARLSFPGYDLSAGNPQGKGYGVTPANYTAGSNIYKGVPA
VGAAFIEGNVSQNANARFGDALPHKIQRD
>WP_236501031.1 hypothetical protein [Thiothrix winogradskyi]
MFKKNKVAVGVATGILGVVGVITSAQAVHVNPDGTGQVLLFPYFNGKGGYVTNINLVNSTDQTKAVKIRF
REGRNSNDILDFNIYMSPEDVWTGTVKNGVNNEGKAVGVLSTTDRTCTLPALASCDDGKCEATVPFSGF
GIYEGVTQEDTKEGYVEVIEMGVVSDTTVQNGVLHTNGKPNNCKTVEDAWNKGTFTQGDGAAAKGLS
APTGGLFGSSAVINIAKGSAFALDPIAIDNYSTQAQHYLSHDPDNFLLPSLASGDVTNSSMMVRSASGDG
EMVVTDWSLQKDACLEDKNELTPRCGINPYPIAHVLLAPHIMNEYFVDPTDGYDGHTDWVVTFPMKKH
GIHGNVAANFENGIFDREEMRAGWSKPNSGFGFSPVLPGKKPESSTLDREVNVISFKSTDPSYDDSRTVL
SSYANQSVTTGSFVSGWARMGFTGYDLKAGFKSAQGYGAKASSYNADSNIYKGVPAVGSAFLEGNISEN
PNARFGDALPHKIQR
>WP_041523952.1 hypothetical protein [Gilvimarinus agarilyticus]
MKKKLLPLAMLAGLAGASGAAQAAYLNSDGLGQVLIFPYYTVNGGNFTNINLVNTTGEAKAVKVRFLEGE
NSQEVLDFNLYLSAYDHWSGAVAMSENADDGSIPWSTGDGEFPARIITNDTSCTAPAIDADGEDFRNFAY
AVDNSGANPVDSSDTNKSLSRTREGYVEVIEMGEIGNVADPDGDPSTDDAFNPASAVTHVDGVPRSCDL
VRNAWNDGGVWDQDPTVGVVAPTGGLYGYASIIDVAEGTNASYDAIAVGDFSDTQLNQDTGSVFPSLT
QALPFSDVFYYDDVAGQGEIAANNFTANSPDTISSLFMRDTLSNDYVLDEAIAANTDWVVTMPTKRFYV
NGFTQTNDNGTPGDTSDDFEEPIAMAPFNSPWDGSTACEHIDIEYWDREEGYPTAPVVPGEIDFSPLPP
QVTVVPDGFNFCTEVSIMTFNGGSVLDSSANLTYGLNLEDGYEHGWARIDLTNAVPPGGNAADYDAREI
TGDVNTFTGLPVVGFAVQKYENGTLEGGVLSNYAGVVVHKYTRDIQ
>WP_261694749.1 hypothetical protein [Tahibacter amnicola]
MKRNNLTTAVVAGIAGLAGIASVANAVELNADGLGQVLIYPYYTVNAGNTTLISVVNTTAVPKAVKVRFLE
GYNSAEVLDFNLFLSQYDVWTASIFTLGDAEGGNLLTDDESCTIPQIKGGTSSSLGTLPDGRKYARFKASAY
LPDPGPDAITRTREGHAELIEMATLAPGAIATAITHEDGVPPGCGAGLSSLVNANLDELRSPTGGLFGSGSI
VNPAIGTIAGYNADAIDGFYTTTDPLQHIYSQPTDVTPNLASARSVEAVAGVARSYNFVSTAAGTGQLIVS
DFVAPIDAVSSIFTASAIHNEYVLEAAAGAESEWVITFPTKRFYVNTGAPTAAAIRPFVENFRAPGRSCVAIG

VSYFNREEQTVQSVDEFSPPDEVPGTNLCFESQVLSFQRSADFTANGNKSKILGSSLTANIDPRSSGFESG
WLNIDLDPETGSTIERHMLRESNPIAPAVEGNTFLGLPVTGFLAVNYINANNGATGILSNYAALYRHRATRT
CVLNDGNTPGACS
>MCF6202246.1 hypothetical protein [Methylococcaceae bacterium]
MFKKTKIAIATAAMLGSVAAIPVAEAVAVNPDGTGQVLIYPYYNTNAGFQTNFSFRNTKDEFKAVKVRLRE
SENSNDVLDFNVYMSPFDVFTFAVKGTASGPVLVTSDKTCTYPAVPATPVPLKGDVYKETTVDDAREGYVE
VIEMGVIDRTIDLDPVGGAAAVNISAGLDHGTTGVPADCSVITKALNEAKWTRGGAKSAPAAYHGGNAS
PAGFLTPTGGLQGSSILLDVANGAAFVADPVALVNYAFDDAGVATGAQYYLPDDANFFLFPSLASGNVMT
SEVIGDAGITMPTANWPLVIDDWGVRDPSAVLTNDTPGRASGLNPFPVSHALAATAINNSYFIDPGFDAA
TDWVVTFPMRKHGIFNNYTYDGGPVVANTHPVLNFVESAAKEDVKYVSSNAFWDREEQQPQAVSTSG
FSPVVGAASTDVILEREVNILTFAPAEGDAKAVLGSGFADPFTMEAGFISGWARLNLSGTYNLTTANARWA
TWTGTPVGPDDVKFSGVPTLGFAAIRGNTGIEGKDVGETVPHSFTRVRGN
>WP_093064250.1 hypothetical protein [Thiothrix caldifontis]
MFKKNKVAISVATGILGVVGVITSVQAVHINPDGTGQVLLFPYYNGTGGYVTNINLVNSTDQTKAVKIRFR
EGKTSHDVLDFNIYMSPEDVWTGTVKNGQDASGNWTGVLSTTDRTCTLPALASCDDGKCLAEQPFNGH
NIYQGVTGADTKEGYVEVIEMGVVDDITVKQGVLHNNGKPNNCKTVEEAWTKGTFTQGEGASAKGLSA
PTGGLFGSSAVINIAKGSAFALDPIAIDNYSTQAQHYLSHDPENFLLPSLASGNVASSSIMVRNASGEGEM
VVTDWNVQADACLEDKDELTPRCGTNPYPIAHVLLAPHVMNEYFVDPTDGYDGHTDWVITFPMKKHG
IHQDVVANFENGIFDREETKAGFSKPNSGFGFSPVLPGKKPDSSTLNREVNVISFKSTDPSYDDSRTVLSSY
AKQSVTTGAFVSGWARLGFTGYDLKAGFKAAQAYGSTAGTYNADSNIYKGVPAVGSAFLEGNISENPNAR
FGDALPHKVQR
>MBJ6609825.1 hypothetical protein [Candidatus Thiothrix moscowensis]
MFKKHKIALGVSAGILGVVGALTSAQAVHLNPDGTGQVLLFPYFNAQQGYQTNINLVNSTDQTKAVKIRF
REGKYSNDILDFNIYMSPEDVWVGTVKAGDDGKGNLIGHLRTTDKSCTLPMEAAADCDSTGADGKNRC
EAVVPFTGHNLYKNVTADDTREGYIEVIEMGVVEDAAVQTGVLHNKGKPNDCKTVEEAWKKGSFTQGA
GSAAKGLSAPTGGLFGSSAVLNVSAGAAFTLDPVAIDNYSTQAQHYLSHDPDKFLLPSLASGNVDSSSLM
VKKPSGEAELVVTTWNQTQDTCLNDGDALTPACGTNPYPIAHVLLAPHLMNEYYLDPSFGYDGHTDWV
VTFPMKKHGIHATKKDVLANFEKGIYDREEGRANWTATPGGQVTDSFGFSPPVGINATVDDSSMLLREV
NVISFKSTDLSYDASRTVLSSPSKQTLSVGPFISGWARLSFPGYDLSSGFKSAAAYSATPSAYAATSGIYKGVP
ATGASFIEGTTGKSGQRVGDALPHKIQRD
>WP_078922529.1 hypothetical protein [Thiothrix eikelboomii]
MFKKKKLALGIVAGLAGALGIVTSAQAVHVNPDGTGQVLIFPYFNAHSGYVTNINLVNSTDKTKAVKIRFR
EGDRSNDVLDFNIYMSPQDVWTGSVRAVEDKDKNLIGSISTSDRTCTLPQLAACKDDKCEVTIPFTGHQIY
TGVDAADTREGYVEVIEMGVVEDKTVQAGVLHKNGTPVNCTAVKDAWKNGIFQQGVGSKATGLSSPTG
GLFGSSAVLNIPQGVAYAIDPVAIDNYSTVAQHYLSDDPNNFLLPSLASGDVTTSTITVQDGSGENELVTTT
WGSVPDTCLNDKDALTPQCGTNPYPISHILLASNVMNEYFLDPSNGYDGHTDWVLTFPMKKHGIHNAD
TDVSILVDGMYDREETSATTTTDTDYGFSPVLPGKTKDETELKREVNVVTFLTSDPSYDDARTVFSSQTGQS
LSVGSFVHGWSSISFPKYDISKGYVSSVTYGKTASVYTAASGIYQGVPVAGFAAIEGNVSSNAGARFGDALP
HKINR
>WP_167202728.1 hypothetical protein [Pseudoteredinibacter isoporae]
MVQNHDGTGQLLMYPIYTANGGNDTYISVANTTADYKAVKVRFVDAMNSQDSLDFHLYLSPQDHWSG
VVTKEGDGAVLKTGDTSCTVPNILAASAVGQLGTTAPMRTFKYGTGGAEADAINNGLDRTLEGHVEIIEM
GVVPAASASNGVAQLAPGSGSATAGAAGDSALVLRSSIKHVNGVPGNCANLETAWRTGGAFFYTDQEN
TPGDAKAATHLQLDAPTGGLYGYGVIINVPDGTAAMFDAVAVDGFMDDGYHPTPGDDEPGVDDGVDN
ATVYIGDSTATNAATYQDFDGAAAAVLAANGVGTTPVAVTGLEHVDPQRGKTGSRFAGGGNSTVTATTA
GNDQEGSLQALSALLTRATVANDYVLEPGLNAATDWVITFPTKRDFVNLSAAAVAANPFGGAGATAGAA
TDAPAPFTSQWDHSKTEACEQIAISYWDREEGVNVITSGFDFSPAPVTTPDVLALCTESNILNFNSKGVL

GGSSRITKSITLANGFNNGWARVSFGVPGAGGTVTAPELSDGSEIVKGLPVIGFAIQKYVNNSATSAGALA
NYAGSIGHHFTNTNDN
>WP_190975830.1 hypothetical protein [Wenzhouxiangella sp. AB-CW3]
MKRNTLTTAVLAGLTGMAGMVSVSNAVNVNPDGLGQVLLYPYYTAHGDNDTLISIVNTTDEGKAVKIRFI
EALNSREVLDFNIYMSAYDVWAAAITRDESTGGGMIRFNDTTCTAPYLLETKGGEQEFLRGAYIGAADDG
GPQGHDRTLSGYVEVIEMGVLTGAAANAAEHVNGVPPNSGVEEKPCSLFTERWITSSAANGTGEWGPP
GNPGFESANYEIDPPSGGLFGSGAVLNVENGTMFSYNATAIDGFWAEQGEHTDPENLFPSLGSGTRFDS
TVFVNGDISDTNWAPADNIHALNHVLSYETLMNEYAIENAIGGSSEWVVTFPTKRFHTDAAPQGPIEGD
TEAVPPFTSVWTADSPIACEELNLSVWDREEQTPDVEGGIGVSPSTTIEDVFELCREANVIRFSNDESLPDS
SEIFGEPSSDVTDLAGYGYVNFNLPSDFQHGWARFDFGNFRSVSSMEGLQVVGLPVIGFWANTYTAGGV
AEGTLANYGGAFAHRGSRTDLVSADD
>WP_248210093.1 hypothetical protein [Lysobacter sp. CAU 1642]
MKKNALTTAVVAGVAGVAGLANVATAVNLNPDGLGQVLIYPYYTVNEGQASLISIVNTTNQAKAVKVRFV
ESLNSAEVLDFNLYLSRFDVWTAALTQDTNGTPVLTTADRSCTVPDVFANQPVAFRPFEFQENNFDIVGLI
DAVRVGGSGGSLVPLSPEERMSQGYLEVIEMGVLFDEGTASSSFRPATWATHGSSGVPSNCAGLVSAWD
SGGVWQSIDPNNQTGPRGVNSPTGGLFGSGVIVDVDFGRALTYNATAIDGFWRNTGLAVNTATTGGEA
DLHFRPGSTFPSLEFARTEADDSATANIFANGSVVSLNYTAGLNAVSAVLMSEGIFNEFNIEQGFLGASEW
VVTFPTKRAHTYQAAAAAGEAQRPFTDAADSEASSSLRDGLLFDTYGICETINTLFFDREERTRAPVGSDFS
PPRPGQARDALCWEANVVAFNQTVTADAPTAVLGTAGKQGAQGITVGTFSAGWVRMDFNAASRNPTS
GLYDSYSNFLVSNPNATTNRRTALVGLPVLGFWAADYQNTAAAAGVRANYSQLHDHRYFRNGFQLPAGT
TGPAYNAPLSVVVGGARLIGADGSCPQGQTCS
>PZQ16550.1 hypothetical protein DI564_07960 [Rhodanobacter denitrificans]
MKKNSLTTAVIAGIAGVAGFASLANAVDLNPDGLGQVLLYPYYTVEKGQQTYLSVVNTSSRGKAVKVRFLE
GYNSREVLDFNLWLSRYDVWTATIFALEDIGLESEGAAILTRDRSCTSPAFSEGDGTVGGAPYVAFRSYAYA
GDSGPQGISRTREGHFEIIEMATLTGSTEGAITHGSSGAPGGCGTVRGLSSANADMSAPSGGLFGGGAVA
NVGQGTYFSFNADALEGWSAEVRFTGTGSLLPSLAQAIDPSGATATAFVPLGTYVATSVYSAANAIDAVSA
TLTADAIYNEYIVEPSIGANSDWVVTFPTKRFYVDSTVSSGPVPIAPFVQAFTGGRSCVVVGINLYNREEGT
TTAAASDFSPTPPGDPPSSLCRETNVISFLDVNTAPTASAVLGSKLVSNVRPSVGNDGWLKLNLNPGSEPH
ALRASTNGNVFHGLPVTGFWATNLVNENVSDGVMSNYSGVYRHRASRSCTAGESTCS
>AKS40876.1 hypothetical protein WM2015_494 [Wenzhouxiangella marina]
MKRNTLTTAVLAGLTGMAGMVSVSNAVNVNPDGLGQVLMFPYYTARGGNDTLISIVNTTERGKAVKIRI
LEALNSREVLDFNIYMSEWDVWTGAITAASDGGGKLLTSDTTCTAPRIFAPSAGIGTDVNGDGVPDFGEI
DFLPFQYTLGNTDFGPQGEERNASGHIEFIEMGTFLPTPASAAAGTEPYVAWSAKHVGPAGAREPNRCAT
FTELWAPANPGAVWRLGDSNFGFEPVGDDTLATGGLFGAASIINVAEGTMFSYNATAVDAFWSTNPLAG
GALHTNPASLLPSLANGTNRTSNVFVNGVVQTNTWPGTVPVAPINATVMFETLMNEYNIEAGLGGRSE
WVLTYPTKRFHTDAQGPVGTVVPNSPIPPFTNTWHRTSSGALNLPCEALSFRVWDREETEPTPDIGEVIP
SPPPPTDAPQVFELCRETNVVRFTLDGDLPAETEILKEPIREGAFERLSYANFQLPDAFQAGWVRFDLTDV
PTGAIIAPGANDPRRTTPADNGDQFEGMPVIGFWVNTFTNGALPGGTLANYGGSFDHHGSRSIVLGSVV
IP
>WP_020561101.1 hypothetical protein [Thiofilum flexile]
MFKQKKLAFGVAAGFLVAAGIFTAVQAVYVNSDGTGQVLLFPYFNANPGYVTNINLVNSTNETKAVKIRFR
EGKNSNDILNFNIYMSPEDVWTGTIRKDANSDAGAFTTLDRTCTMPVQHAADCSSPNKDRCEVMKTFD
GKNIVYENKDTREGYVEVIEMGVVTDPKIQEAVKHVNGKPANCQAVSDAWLTTGAFASGTGAAAVGLK
APTGGLFGSSAVLNIAEGAAFAIDPVAIDNYSTQAQHYRPDLYEEYLLPSLASGNVTQSTITVPKSSGESDLV
VTNWKQQSQDPCTVEGTSCGTNPYPMAHVLAVPYIMNEYFLDPTDGYDGHTDWVVTFPMKKHGIHQ
ASTDVVAKIDGLYDREEARAWGLNATQDKYFGFVTDAKAGTFGPEASLTREVNVLSFVSTDPSYDLNRTV

FSSPALKAVSAGPFVHGWARITFPNYTLGGAFTSSNAYGANPYNYKADHFIYNGVPTLGFAAIEGNVSENA
HARFGDAIPHKTRRY
>WP_020208123.1 hypothetical protein [Gilvimarinus chinensis]
MKKKLLPLAMLAGLAGAAGTAQAVYVNTDGHGETLIYPFFSVEEGQNTYINVTNTTDQYKAVKVRFLEG
QNSAEVLDFNLYLSPEDVWTGAIVAIEGGGTKVITRDNSCTVPWIPRPNAEGEGSDGVEFRTYEYQGDG
GDQTVARTQEGYVELIEMGVITDPDVQAEIQHNAAGMPGDCDGLRARWMENGVSPSATSESFENPAD
VVGNGYWTATPNEGFAVQADQDTEYTLGGLYGYGVLINPAEGSGSAYNAVALDQFFGAAVVDGLYQPQ
HTNPGTTLPSLGSAYEIAQVIDGNAIVDEVMTTGWDAVSAVFMHDTLSNDFVLDAAVNADTDWVITMP
TKRFYVNQGLDSDDEPNDALPPFTNAWSGGTACEVVGIEAWDREEAVTELTGDIDFSPRPPAADGPEPSS
ICSEVNVLTFDDASAVYPSPRIQYGMPELGFENGWVRLDFSSDTAGERELESADGAIFEGLPVTGFAVQKFI
NGSMDGGTLANYAWLVQHNYTRSITPSSL
>MAT91730.1 hypothetical protein [Halioglobus sp.]
MKKILPVAVLAAMAGVNGAQAVHVNSDGLGQVLLFPYYTTTGGQDTLINIANTTADYKAVKVRILESLNS
REVLDFNLYLSPFDHWSAAISADPDGVGAAITSGDNSCTVPLAISNGDTIPFRNFEYDEDSIDGLERTKEGY
IEIIEMGIVEVGTQNWPQDIKHVNGVPGDCQDLDDSWNNGVWASDSQDGMDSATGGLYGYGVLIDVE
EGTDATYDAVALDNFFDTGVIFHTAPGSLDPGLDDGTPDYDVIIGNTVVSGSAAGRPGISAGLDATSAVLM
VDTISNDYVLDSDLDAGTDWVVTFPTKRDYVVPFPAIDPFTDTWDPLESESCEGFALAYVDREEGSPLAPP
TSNDFSPQPDPDEPELFTFCAEANVLSFNSSNVLEAPQDRNGTNFDLEEGFENGWARLDFKDTDANGSS
DRPSLPAGGTDFLGLPTIGFAVQKYVNGDLDGVLSNYAGSITHKGSRSVLASVSVP
>WP_271503438.1 hypothetical protein [Luminiphilus sp.]
MKKKVLPLAVGAATAVTMSAAHAAMYVNDKGMGETLIFPFYSAESGNNTAIHVVNTTAGTKAVKVRIM
EAENSQEVLDFNLYMSPADHFSFGIVADGDGAKIVTGDNSCTVPAIPADGQPFVSFKYDGTIGAGDSSEG
DDDGVGAFDNTGIERTRVGYVEVIEMGQLDPDAAPALDTTPASDTYTPINAVDAITHGADGVPANCDLL
VEAWSLIDDVPGTWYAEADDNVTQATAEFLTNWAGGGLYGYARVLNVAEGSAFGYDALAIASHVAAGAS
GSAMHYRPGSIYPNFGDDAMDTEATINVNGEAITLDFDGTVYPADSTNRVQALNSLIMATAIHNDFVTDS
SINAETDWITTFPTKHFHINGVTEGVEPFATLWNGQSACEDTNLAPIDREEQTPVIPDEPTTSAGPDFSPA
PPPGAGPAAPASTDVPLCYESTVVMMAETSAVKAENSVGLGVNASLHADDGWATMSFDPASLDDDIPF
SCDVDGEALENEVAEGATPCDRVIDAGTHTVTGLPVVGFAVQKYVNLEATPGGAGYYAMATAHKTSVTV
SEDN
>RZO79309.1 hypothetical protein EVA63_08995 [Halieaceae bacterium]
MKKNILSLAVASSVAGLAVTAQAAMYLNPEGTGEVLLFPYYNAQNGNETSMHIVNTTADAKALKVRFME
YVNSQEVLDFNLYLSAGDHFAFTIFQNPNGDGGAIITRDNSCTVPELGTGSGDFGGTQTDNADGTTTRIQ
PFVNYAYGVSGTGVTADNFSSITRSLAGHVEVIEMGVLDDTTPGSTTAFNPESWATHGSDGVPANCAGLV
AAWSVTAGANGAWLTDKTDEIEAPSGGLYGVANMLNNSDSAAYGMEAAAIADFWANNVTSGHEKPG
DALPSLAQGDTSSIVPNDGAAYTNAFASGINAVSSLFMAKSISNDVMANPALSGETDWVVTFPTRRYYV
NTSPAALPFTDPYVGSGTAGGPETSCETVAISQTDREESTTDAGSTGPAFSPAPPSQPGVTGPQLCYETNTI
AINGVSALNASVSTATVADVAKTLAWGEDQPEGWQTITFSANNYMDSTDGDAAKRRIQGLPVMGFAAF
EYTNATSNFGFVSDHKTSIAGSGINP
>WP_116367404.1 hypothetical protein [Parahaliea mediterranea]
MKASFKPLGLAAAVAAASAGYTGVAQATVASNQLGDLALIPYYTVENGFVTGVHITNTSEVTQVVKLRMR
RAMDSMDALDFNLILSPKDEWTGWIDATQLDEDSPTEIAFNTNDNSCTAPQSPNGRFVMPDDMTDST
TVDFRTGAEEGYIEVIAMGQTDGLQPIDTAALHGSDGVPFDCNAVASNFYRATAYDNAGTVTPDLKQNG
VVATNTTHQCKGDTTTGLAPENCGNGAVVPNTFVDSENALKVSYFVRDAEGGLEMGSNAVHIQDFNTV
PTLTNQQTILAGTFDPMGYLFPDLDGGSPYVPAGDVVNRGLFDTVVRPDLGAQMIVNDWSTNPASNVR
TDWVVTFPGQYTMLNLFDYIRALPDVTATTDSTGVCTFASLCDYRDIPVNVELTVYDREEQSFTPAEGGLV
VSPSVSATPEGFVLRNEVNVVQWASAGVTPDPVLDSNYAVTNTVQLANAVNGWAELDVTADPAKGGA
QSVFQYGTDPNSATFDPVTDVAQVPMIGFTAWERSFPSNPDANYGRIIDHSFVVSS

>WP_163495757.1 hypothetical protein [Kineobactrum salinum]
MNANFKPLGLAAAVAAVTAGYAGITTAQEPSINGLGNVGLVPYYTVRDGFGTGVHITNTSEATQVVKVRL
RRGTDSMDALDFNLILSPKDVWTGFLSTDGDNIVFTTQDESCTAPGRVNGQFTMPPIYRENAEEGYIEIIG
MGQPVSESTPIAVAAKHTSEGVPADCAGVRSNFFANGDAGGPSQGVIDYDETYQISTVTGVPSGVTIYEA
PENALKVSFFIRDSESGIEFGNEAFHLQNFLAEPSMTNQQTGLFSGDLEGFDYPDLNGGWVGVDRGKFN
ALRTAFGSASVINDWSANADLNVGTDWVITFPGQYTMLSLPHYFGSLFNEAVTCSRGDPATAGGGNDDL
ETAACDFRDIPATATFDVYDREEQQITQEEGGLVVSPQPPGQVTRTELPYEVNVVRWGVDPVLNSQNSDI
SVDVPDSPFGWAELAVTSMDANLAVCDATALSAGLTLGDTNTVTEDSVAAAAAAQDCTDLSTVNLIPKIG
FVAWERGFPANPDANYGRIVEHSYGVAS
>WP_035515409.1 hypothetical protein [Pseudohaliea rubra]
MKSNFKPLGIAAAVAVASAGYANVASAQTVANNALGDLALVPYYTVNGDWLTGVHIVNTSAQTQVVKFR
FRRATDSMDALDFNIVMSPEDVYAGYLSDDDGAISWQADDTTCTVPATTGGKLTMPEIYRDDAETGYVEI
IAMGAPEFETSPIAEAAKHKAGVPADCAAVRSNFFADGSSTSGSETRGVIDFETTYGVDADATDTVDFGG
ESTYTDSGNVLKVSYFIRDNATGVEFGDNAVHIQDFLENPSITNQQYGVFSGDLNGFDFPDLDGGVPTAS
PAERGKFVALRSGSVLGVSKLINEWTANTANGAALDWVVTMPGQYTMLDLPNYIEEELDADGAAVLDE
DCKRGELNKACDYRDIPVTATIVPYNREELTSTPEEGGLTVSPAPPGEVQRLVLPNETNVITFGNKDQNGVL
GVIGDVDITADLEQPYGWLSLEVASATTGDTAVCDWDVNTRVTNGEYSAAAGADLTMTCSSVGGNVPM
IGFAAWARQVAANPDASYGRIVAHSFESAAP
>WP_027949665.1 hypothetical protein [Haliea salexigens]
MNAKFKPLGLVAAVAAATAGYNGVANAQLELAASSDLGNLALIPYYTVRDGNVTGVHLTNTSDSTQVVK
VRLRRGTDSMDALDFNLILSPKDVWTGFLAAEGENVVFNTTDSSCTAPATVNGKFTMPGIYRADADEGYI
EIIGMGQPLTETAPIAVSAKHGAEGVPASCTFVRDNFFANGSPGTTRGVVDGATSVQRVPVGTEGATTLA
LNEYEAPDNALKVSYFVRDAGSGREFGNSATHIANFLVEPSITNQQFGLFSGDLLGFDYPDLNGGTGAGID
RDRFKELRAVLGGASVINDWSANADLNVGTDWVITFPGQYTMLDLPHYFASLLTAGEADPDDRIPCNAG
LPLPDGVFAGLTGACDHRDIPAVATFDVYDREEQQRSQPDGELVVSPALPGQVVQTLLPFEVNVVQWGA
EPVLGSENSDIVVAVPDSPFGWAELNVTSSNSKVQAVCEASAESANFNIGEGFDAIVGAVTAYGCNSTDV
NADEIPKIGFVAWERSFPANPDSNYGRIVEHSYGGVGS
